# Efficacy and Safety of [^177^Lu]Lu-DOTA-TATE in Adults with Inoperable or Metastatic Somatostatin Receptor-Positive Pheochromocytomas/Paragangliomas, Bronchial and Unknown Origin Neuroendocrine Tumors, and Medullary Thyroid Carcinoma: A Systematic Literature Review

**DOI:** 10.3390/biomedicines11041024

**Published:** 2023-03-27

**Authors:** Marianna Hertelendi, Oulaya Belguenani, Azzeddine Cherfi, Ilya Folitar, Gabor Kollar, Berna Degirmenci Polack

**Affiliations:** 1 Advanced Accelerator Applications International SA, 4 Rue de la Tour de l’Ile, 1204 Geneva, Switzerland; 2 Novartis Pharma AG, 4002 Basel, Switzerland; 3 Novartis Pharmaceuticals, One Health Plaza, East Hanover, NJ 07936-1080, USA

**Keywords:** pheochromocytomas, paragangliomas, bronchial neuroendocrine tumors, unknown origin neuroendocrine tumors, medullary thyroid carcinoma, [^177^Lu]Lu-DOTA-TATE

## Abstract

Background: We have performed a systematic review to evaluate the efficacy and safety of [^177^Lu]Lu-DOTA-TATE, a radioligand therapy, in advanced somatostatin receptor-positive pheochromocytoma/paraganglioma (PPGL), thymic neuroendocrine tumor (NET), bronchial NET, unknown primary NET, or medullary thyroid carcinoma (MTC). Methods: Studies identified in PubMed from inception to 13 May 2021 must have assessed [^177^Lu]Lu-DOTA-TATE as a single agent and reported outcome data for the specific NET types of interest. Results: Two independent reviewers performed the screening and data extraction, resulting in 16 publications: PPGL (*n* = 7), bronchial NETs (*n* = 6; one also included NETs of unknown origin), and MTC (*n* = 3). Overall, [^177^Lu]Lu-DOTA-TATE offers encouraging antitumor activity (overall tumor response rates and disease control rates) across NET types. Safety was favorable with most adverse events mild to moderate in severity, transient, and consistent with those seen in patients with gastroenteropancreatic (GEP)-NETs. Conclusions: [^177^Lu]Lu-DOTA-TATE has been used effectively in clinical practice to treat NETs of non-GEP origin.

## 1. Introduction

Neuroendocrine neoplasms (NENs) are rare tumors that are derived from sensory and secretory neuroendocrine cells and can occur at almost any anatomical site [1,2]. Neuroendocrine tumors (NETs) are a subset of well-differentiated NENs that widely express the somatostatin receptor (SSTR), particularly Subtypes 2 and 5 [1,2,3]. The most common location of NETs is the gastrointestinal tract, including the pancreas [4]. Many NETs have malignant potential and commonly metastasize before diagnosis, complicating management and limiting the potential for curative surgery [3].

[^177^Lu]Lu-DOTA-TATE is the first radiolabeled somatostatin analog (SSA) approved for the treatment of SSTR-positive gastroenteropancreatic neuroendocrine tumors (GEP-NETs) in adults [5,6]. This approval was supported by the randomized Phase III NETTER-1 trial, in which 231 patients with advanced, progressive midgut NETs were treated with four cycles of 7.4 GBq [^177^Lu]Lu-DOTA-TATE plus octreotide long-acting release (LAR) or high-dose octreotide LAR [7,8]. [^177^Lu]Lu-DOTA-TATE was associated with a significantly longer progression-free survival (PFS) compared with high-dose octreotide LAR (hazard ratio 0.18; 95% confidence interval [CI] 0.11–0.29; *p* < 0.0001). The response rates for the [^177^Lu]Lu-DOTA-TATE and high-dose octreotide LAR groups were 18% and 3%, respectively (*p* < 0.001), and after a median follow-up of over 6 years the long-term safety profile for [^177^Lu]Lu-DOTA-TATE was favorable [7,8]. Creatinine clearance analyses over time were similar for both treatment groups, indicating no long-term renal toxicity after [^177^Lu]Lu-DOTA-TATE treatment. Two randomized patients treated with [^177^Lu]Lu-DOTA-TATE developed myelodysplastic syndrome (MDS) and no cases of acute myeloid leukemia were observed [7].

Although most published evidence and experience with [^177^Lu]Lu-DOTA-TATE is in GEP-NETs, [^177^Lu]Lu-DOTA-TATE may be of benefit to patients with SSTR-expressing NETs that arise in other locations [9]. Well-differentiated bronchial NETs, the second most common type of NET, are classified as typical (low-grade) or atypical (intermediate-grade) carcinoids, with the same terminology used for the much less common thymic NETs [1,2]. Up to 70% of bronchial NETs express SSTRs [10]. For some patients (9–14%) with metastasized NETs, the primary tumor is unknown [11,12]. The single-arm ERASMUS Phase I/II study evaluated [^177^Lu]Lu-DOTA-TATE in patients with a variety of NET types, including midgut, hindgut, pancreatic, bronchial, and unknown origin [13]. A retrospective analysis of Dutch patients with bronchial and GEP-NETs enrolled in ERASMUS and treated with [^177^Lu]Lu-DOTA-TATE (*n* = 443) reported an overall response rate (ORR) of 39% and a median PFS of 29 months [13].

Other NETs include pheochromocytoma/paraganglioma (PPGL) and medullary thyroid carcinoma (MTC). PPGL arise from the chromaffin cells of the adrenal medulla (pheochromocytoma [PCC]) and from sympathetic or parasympathetic ganglia (paraganglioma [PGL]) [2,14]. MTC, which is usually well differentiated, comprises the vast majority of thyroid NENs [2]. The remaining NETs, such as pituitary, head and neck, thymus, breast, and genitourinary system, are rarely encountered in clinical practice [1,2,15].

There is currently a high unmet medical need for bronchial NETs, thymic NETs, NETs of unknown origin, and PPGL, with limited approved therapeutic treatment options [16,17,18,19]. The National Comprehensive Cancer Network^®^ (NCCN) includes treatment with [^177^Lu]Lu-DOTA-TATE as an option for patients with SSTR-positive bronchial or thymic NETs who have progressed on standard-of-care regimens and as a primary treatment for SSTR-positive PPGL with distant metastases. As for NETs of unknown origin, NCCN recommends that they are treated similar to GEP-NETs [19]. Guidelines from the European Society for Medical Oncology (ESMO) also include peptide receptor radionuclide therapy (PRRT) as a potential therapy for patients with metastatic bronchial or thymic NETs (third-line) [16], advanced PPGL (after progression on standard of care or for those with high tumor burden) [17], and in select cases of MTC, such as when multikinase inhibitor therapy is contraindicated [18]. To further understand evolving clinical practice and describe the use of [^177^Lu]Lu-DOTA-TATE as a treatment for NETs of non-GEP origin and unknown origin, we performed a systematic literature review to identify and summarize published evidence for the efficacy and safety of [^177^Lu]Lu-DOTA-TATE in adult patients with inoperable or advanced SSTR-positive PPGL, thymic NET, bronchial NET, unknown primary NET, or MTC (collectively known as non-GEP-NETs for the purpose of this review).

## 2. Materials and Methods

### 2.1. Search Strategy

PubMed was searched for studies published up to 13 May 2021. No geographic, language, or age restrictions were applied in the search, but only English-language publications reporting studies in adults were selected for inclusion in the systematic review. The search and report were completed according to the PRISMA (Preferred Reporting Items for Systematic reviews and Meta-Analyses) 2020 statement [20]. The review protocol was registered with INPLASY (INPLASY202330030).

The search strategy included both Medical Subject Headings terms and free-text terms, and included variants of the terminology for the NETs, such as *cancer*, *carcinoma*, *carcinoid*, *tumor*, *tumour*, and *neoplasm*, as well as variants for LUTATHERA^®^, such as *[^177^Lu]Lu-DOTA-TATE*, *lutetium Lu ^177^ dotatate*, *lutetium (^177^Lu) oxodotreotide*, *lutetium oxodotreotide Lu-^177^*, *(^177^Lu-DOTAOTyr3)octreotate*, *DOTATATE-^177^Lu*, *^177^Lu-DOTATATE*, and *(^177^lutetium-DOTA(O)Tyr3)octreotate*. The complete search strategy is given in Supplementary Materials S1.

### 2.2. Study Selection Process

The titles and abstracts of records identified on PubMed were screened initially to exclude any duplicate records and those that were clearly not relevant (Supplementary Materials S2). A more detailed screen of the remaining records was then performed, using the inclusion and exclusion criteria below, to select records for the further evaluation of the full text to establish eligibility. The selection process was performed by two reviewers independently and any differences were resolved by consensus. In addition, the reference lists of reviews were examined to identify additional studies that had not been detected by the initial search strategy.

### 2.3. Inclusion and Exclusion Criteria

This systematic review considered studies that included adults (as defined by the authors) with any of the following inoperable or metastatic SSTR-positive NETs: PPGL, thymic NET, bronchial NET, NET of unknown primary origin, or MTC. Studies that included multiple NET types were only included if the results and baseline characteristics were provided for individual NETs. Studies that included both pediatric and adult patients were retained, if it was possible to extract data for adults only.

Articles not written in English were excluded. Studies reporting the efficacy or safety of [^177^Lu]Lu-DOTA-TATE in combination with other anticancer treatments, including other radioligand therapies, were excluded (except for SSAs, amino acids, and antiemetic agents, per the treatment regimen) [5]. In addition, studies were excluded where relevant outcome data (response rates, survival time, or safety) were not available for the specific NET types of interest or the specific radioligand treatment of interest. Individual case reports were excluded, as were case series that only reported relevant tumor types in a single patient.

### 2.4. Types of Intervention

Studies that evaluated the efficacy and safety of any [^177^Lu]Lu-DOTA-TATE treatment administered as a single agent only were included. 

### 2.5. Types of Outcome

The following efficacy or safety outcomes were analyzed: PFS, time to tumor progression (TTP), disease control rate (DCR), response rates (as reported in each study), overall survival (OS, as reported in each study), mortality, and adverse events (AEs) by organ and type.

### 2.6. Types of Study

Randomized controlled trials, non-randomized controlled trials, quasi-experimental studies, prospective and retrospective cohort studies, and case series (if the patients were analyzed or analyzable as a group) were included. 

### 2.7. Data Extraction and Synthesis

Qualitative and quantitative data were extracted from the studies by two reviewers independently, and descriptive analyses performed. Efficacy data are presented by NET subtype and safety data are summarized for all NETs analyzed. If not reported, ORR was assessed by integrating the number of patients with complete and partial radiologic response by the total number of patients treated.

## 3. Results

**Figure 1:**
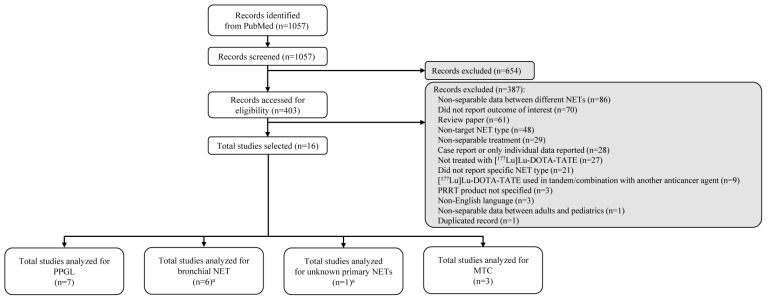
Summary of literature search. ^a^ One publication reported data for bronchial and unknown primary NETs. MTC—medullary thyroid carcinoma; NET—neuroendocrine tumor; PPGL—pheochromocytoma/paraganglioma; PRRT—peptide receptor radionuclide therapy.

The search identified 1057 records, of which 654 were eliminated in the initial screen for not meeting the eligibility criteria (Figure 1). During the second screen, 387 of the 403 remaining records were excluded for the reasons shown in Figure 1. A total of 16 publications were included: seven on PPGL, six on bronchial NETs (one of which also included NETs of unknown primary origin), and three on MTC. No eligible publications for thymic NETs were found. Although one publication on PPGL did not explicitly state that patients had SSTR-positive lesions [21], it is a requirement for treatment with [^177^Lu]Lu-DOTA-TATE; therefore, it was assumed that patients had SSTR-positive lesions. The key study design features and patient characteristics are summarized in Table 1 [21,22,23,24,25,26,27,28,29,30,31,32,33,34,35,36], and [^177^Lu]Lu-DOTA-TATE efficacy data by NET subtype are presented in Table 2 [21,22,23,24,25,26,27,28,29,30,31,32,33,34,35,36]. Safety data are summarized in Table 3 [21,23,24,25,26,27,28,31,32,33,34,35,36].

### 3.1. PPGL

For PPGL, one prospective cohort [22], one retrospective cohort [23], and five retrospective case series [21,24,25,26,27] were identified, and overall [^177^Lu]Lu-DOTA-TATE was assessed in a total of 97 patients (range 4–30 per study).

More patients had a PGL diagnosis (*n* = 81 [84%]) than PCC (*n* = 15 [15%]), with one patient diagnosed with PGL and PCC. The age range of treated adults was 22–84 years, and most patients had received prior surgery and had metastatic disease at baseline (Table 1). In general, four cycles of [^177^Lu]Lu-DOTA-TATE treatment (5.55–7.4 GBq per cycle) were administered, but some patients received less, and one study recorded more, with extra cycles given as salvage therapy (overall, 1–11 cycles were administered; Table 1). Some of the primary reasons for not receiving the recommended four cycles of therapy included toxicity and disease progression.

Five of the seven studies assessed the radiologic response using RECIST 1.1 (Response Evaluation Criteria In Solid Tumors), one used SWOG (Southwest Oncology Group) criteria, and one did not specify (Table 2). Partial response (PR) was recorded in six of the seven studies, ranging from 7% (1/14) to 25% (1/4), but no complete response (CR) was reported. ORRs calculated from response data ranged from 0% to 25%. Three studies included minor response (MR) in the response criteria, and this ranged from 8% (1/12) to 29% (4/14). DCRs ranged from 67% to 100%. Overall, out of the 97 patients with PPGL treated with [^177^Lu]Lu-DOTA-TATE, 13 achieved a PR, 7 had MR, 64 had stable disease (SD), and 12 had progressive disease (PD) (Table 2). Response was unknown for one patient.

Two studies evaluated PGL and PCC separately [24,25]. Best response to [^177^Lu]Lu-DOTA-TATE was evaluated in 37 patients with PGL in total and was PR (*n* = 7), MR (*n* = 4), SD (*n* = 24), and PD (*n* = 2). The best responses in five patients with PCC were PR (*n* = 1), SD (*n* = 1), and PD (*n* = 3). SD was observed in one patient with PCC plus concomitant pancreatic NET (pNET), and in one patient with PGL plus PCC and concomitant pNET (Table 2).

Median PFS was reported in three studies: 21.6 (range 6.7–138) months, 30 months, and not reached (NR) after a 40-month follow-up [23,24,26]. Median OS was assessed in four studies, with three reporting that the median was NR (with median follow-ups of 40, 52.5, and a range from 26 to 84 months), and one reporting a median OS (range) of 49.6 (8.2–139) months (Table 2) [21,23,24,26].

### 3.2. Bronchial NETs

The search identified three prospective cohort studies [28,29,30] and three retrospective cohort studies [31,32,33] that enrolled a total of 137 patients (range 6–48 per study) with bronchial NETs (Table 1). All patients were treated with [^177^Lu]Lu-DOTA-TATE and the efficacy data are summarized in Table 2.

Most of the patients had metastatic disease at baseline with liver and bone identified as common metastatic sites. Four of the six studies recorded a carcinoid subtype, and between 31% and 77% of patients were diagnosed with atypical carcinoid (AC) tumors. The age range of treated adults was 37–79 years, and most patients had received prior surgery. Patients received between 1 and 8 cycles of [^177^Lu]Lu-DOTA-TATE (3.7–7.8 GBq per cycle; Table 1). Some studies stratified patients according to risk factors for kidney and bone marrow toxicity and reduced the dose of [^177^Lu]Lu-DOTA-TATE per cycle; however, the cumulative administered activity was similar across all the studies.

Four of the six studies assessed radiologic response using RECIST, and two used SWOG or modified SWOG criteria (Table 2). Of the patients assessed for tumor response, most studies reported PR (12–56%) or MR (11–17%) as best response following [^177^Lu]Lu-DOTA-TATE treatment, while one patient achieved a CR. SD was observed in 22–83% of patients. ORRs calculated from the response data ranged from 13% to 56%, and DCRs of between 62% and 100% were observed (Table 2). One study analyzed the outcome by the histopathological subtype and reported a DCR of 80% for typical carcinoid (TC) tumors (*n* = 15) and 47% for AC tumors (*n* = 19) [28]. Overall, five studies reported analyzable response data for 119 patients treated with [^177^Lu]Lu-DOTA-TATE, with one achieving CR, 22 PR, 9 MR, and 54 SD (Table 2).

Five of the six studies reported a median PFS of between 18 and 31 months, and one reported a TTP of 31 months (Table 2). Two studies recorded a similar median OS of 42 and 48.6 months [28,33], and the OS was NR in two studies [30,31]. One study analyzed survival outcome by subtype; the median PFS for patients with TC (*n* = 15) and AC (*n* = 19) were 20.1 (95% CI 11.8–26.8) and 15.7 (95% CI 10.6–25.9) months, respectively, and the median OSs were 48.6 (95% CI 26.0 months–NR) and 37 (95% CI 18.7–68.9) months, respectively [28].

### 3.3. NETs of Unknown Primary Origin

Eight patients, aged between 54 and 80 years old, with metastatic NETs of unknown primary origin were included in a Swedish prospective study [30]. Seven of the eight patients had extensive disease and most (87.5%) had received prior chemotherapy. Patients received between two to seven cycles of 7.4 GBq [^177^Lu]Lu-DOTA-TATE until the absorbed dose to the kidneys reached 23 Gy or until there were other reasons for stopping (Table 1). The best response by RECIST 1.1 was PR (38%), SD (50%), and PD (13%) (Table 2), giving an ORR of 38% and a DCR of 88%. The median PFS from treatment initiation was 17.5 (95% CI 7–34) months and the median OS was 43 months (95% CI 15 months–NR) (Table 2) [30].

### 3.4. MTC

The study and patient characteristics for the three studies that analyzed the data of 62 patients (7–43 per study) with MTC are summarized in Table 1 and the results in Table 2. One of the three studies was a prospective, single-arm interventional study [34], and the other two were retrospective, single-center case series [35,36].

Overall, patients were aged between 19 and 80 years and all patients had metastases at baseline, with approximately one in three patients having extensive metastatic disease (Table 1). In general, three or four cycles of [^177^Lu]Lu-DOTA-TATE treatment (5.55–7.4 GBq per cycle) were administered (Table 1). The intended cumulative activity was up to 29.6 GBq. The average cumulative activity was lower at 18.5 GBq for one study, which administered an average of three cycles of the lower dose of [^177^Lu]Lu-DOTA-TATE (5.55 GBq per cycle).

Tumor response was assessed using RECIST 1.1 in all studies. PR was recorded in two of the three studies (5% [2/43] and 43% [3/7]), but no CR was observed. DCRs ranged between 40% and 86%. Overall, out of the 60 patients with MTC that were treated with [^177^Lu]Lu-DOTA-TATE, five achieved a PR, 32 had SD, and 23 had PD (Table 2). The median PFS was measured in two studies and was 24 (95% CI 15.1–32.9) months and 0.7 (range 0.3–12.0) years. The relatively short PFS observed in the latter study (0.7 years, ~8.4 months) was attributed to many of the patients having poor prognostic features including paraneoplastic endocrine syndrome, loss of tumor marker expression, and old age (median 62 years) [36]. The respective OS data were 26 (95% CI 16.6–35.3) months and 1.14 (range 0.4–12.0) years [35,36].

### 3.5. Safety of [^177^Lu]Lu-DOTA-TATE

Three studies reported safety on populations that included patients with NETs or SSTR-positive tumors that are not the primary focus of this review. Specific data for PPGL, bronchial NETs or NETs with unknown primary origin could not be delineated, and for this reason, these three studies have been excluded from the safety analysis [22,29,30]. Safety data from the remaining 13 studies for patients treated with [^177^Lu]Lu-DOTA-TATE (*n* = 269) are summarized in Table 3. One study reported that persistent thrombocytopenia limited the number of cycles of [^177^Lu]Lu-DOTA-TATE administration in three patients, so they received only two or three cycles. When reported, AEs were generally mild and resolved without sequelae. One study described acute toxicity of nausea (34%), pain (23%), and vomiting (13%) after the administration of [^177^Lu]Lu-DOTA-TATE.

Of the 11 studies that reported on hematologic toxicity, most events were mild; only three studies, encompassing 70 patients, recorded 14 events of Grades ≥3. Thrombocytopenia, leukopenia, and anemia were the most frequently cited events (Table 3). Eight studies included data on renal toxicity, recording either no nephrotoxicity or no high-grade nephrotoxicity.

Few high-grade AEs were reported. In a retrospective case series (*n* = 30), two patients experienced reversible cardiac AEs after their first [^177^Lu]Lu-DOTA-TATE cycle. One patient with PGL and lung and bone metastases experienced cardiac failure, possibly due to chronic catecholamine release. Another patient with PCC developed pleural effusion and delirium that may have resulted from cardiac failure or catecholamine release. Both patients made full recoveries and successfully received further cycles of [^177^Lu]Lu-DOTA-TATE treatment [24]. In a small retrospective case series (*n* = 5), one patient with PGL that had metastasized to the lungs, bone, and mediastinal and supraclavicular lymph nodes received three cycles of [^177^Lu]Lu-DOTA-TATE and stopped further treatment after experiencing suspected pneumonitis considered plausibly associated with treatment [21]. In a different retrospective study, one patient with bronchial NET and mediastinal invasion developed Grade 3 radiation pericarditis following two cycles of [^177^Lu]Lu-DOTA-TATE. After a successful pericardiocentesis procedure; this patient went on to receive a further two cycles of [^177^Lu]Lu-DOTA-TATE [32]. One patient with MTC developed Grade 3 hemoptysis, which was attributed to the progression of pulmonary metastases [36].

Overall, secondary hematologic malignancies were reported in one patient who was treated for PPGL. This individual developed MDS, which was considered to be possibly related to the [^177^Lu]Lu-DOTA-TATE treatment (cumulative dose 44.4 GBq), given that they had not received prior chemotherapy and did not present with bone metastases [24].

## 4. Discussion

Sixteen studies involving 304 patients were identified and provided data for the efficacy analysis of the [^177^Lu]Lu-DOTA-TATE treatment in SSTR-positive PPGL (seven studies, 97 patients), bronchial NETs (six studies, 137 patients), NETs of unknown primary origin (one study, eight patients), and MTC (three studies, 60 patients). One publication reported data for bronchial and unknown primary NETs. The safety analysis included 269 patients; three studies did not report safety data by a NET subtype and so were excluded from the safety analysis. The dosing of [^177^Lu]Lu-DOTA-TATE was similar for all indications with most studies recording an average of four cycles and administering 7.4 GBq per cycle. 

Most of the studies assessed radiologic response using the RECIST 1.1 criteria. Based on the SWOG criteria, a CR was achieved by one patient with bronchial NET. ORRs, calculated using response data as measured by RECIST 1.1, varied from 0% (PPGL study with six patients and an MTC study with 10 patients) to 43% (MTC study with seven patients) but the majority of studies (9/11) had ORR rates of up to 27%. This is broadly in agreement with the ORR of 18% (one CR and 17 PR) observed with [^177^Lu]Lu-DOTA-TATE treatment in patients with advanced midgut NETs enrolled in the NETTER-1 study (101 evaluable patients) [8]. The highest calculated ORR, as measured by the modified SWOG criteria, was 56% (bronchial NETs study with nine patients). Calculated ORRs assessed using RECIST 1.1 for bronchial NETs were 17% and 27%, lower than that reported in the ERASMUS study (30%) [37]. In this study, calculated DCRs (RECIST 1.1) with [^177^Lu]Lu-DOTA-TATE were slightly higher for PPGL, bronchial NETs, and NETs of unknown origin (67–100%, 68–100%, and 88%, respectively) compared with MTC (40–86%). Recent meta-analyses of [^177^Lu]Lu-DOTA-TATE in advanced NETs of mixed origins reported ORRs of between 25% and 33% and DCRs of between 74% and 83% [38,39,40], similar to those identified for each NET subtype analyzed in this study, suggesting that NET origin does not dramatically affect the efficacy of [^177^Lu]Lu-DOTA-TATE. A meta-analysis of radioligand therapy ([^177^Lu]Lu-DOTA-TATE, [90Y]Y-DOTATOC, or [90Y]Y-DOTA-TATE) in PPGL (*n* = 201) calculated an ORR of 25% and a DCR of 84%, which are within the ranges observed with [^177^Lu]Lu-DOTA-TATE in our study (7–25% and 67–100%, respectively) [14].

Survival outcomes were not consistently reported in the studies analyzed. Median PFS, when reported, ranged from 0.7 years (8.4 months; MTC study) to 31 months (bronchial NETs), with no obvious pattern according to NET subtype. The latter is comparable to those observed in the ERASMUS clinical trial setting when the overall median PFS was 29 months, with 20 months recorded for bronchial NETs and 29 months for NETs of unknown origin [13]. The patient population in the MTC study that reported a relative short PFS of 0.7 years (8.4 months) included a number of factors associated with a poor prognosis (paraneoplastic endocrine syndrome, loss of tumor marker expression, and older age) that the authors believed may have contributed to the poor survival outcome [36]. The follow-up periods for many studies were not long enough to measure median OS, but for those studies that did report data, the values were comparable for three NET subtypes (49.6 months for PPGL, 42–48.6 months for bronchial NETs, 43 months for NETs of unknown origin) but shorter for MTC (26 months and 1.14 years [13.7 months]). NETTER-1 reported a median OS value of 48.0 months for patients with midgut NETs, which is consistent with those recorded for PPGL, bronchial NETs, and NETs of unknown origin in this analysis [7].

The survival benefits observed with [^177^Lu]Lu-DOTA-TATE in non-GEP-NETs are encouraging when compared with the current standard of care for these indications. In PPGL, [131I]meta-iodobenzylguanidine (MIBG) and systemic chemotherapy are used to treat progressive disease [17]. In a Phase II study, the median OS with [131I]-MIBG was 36.7 months, and a retrospective analysis of patients receiving systemic chemotherapy reported a median OS of 6.4 years (76.8 months) for responders and 3.7 years (44.4 months) for non-responders [41,42]. Everolimus is often used as first-line therapy to treat patients with bronchial NETs who have progressed on SSAs [16]. In subgroup analyses of patients with progressive bronchial NETs and NETs of unknown origin from the RADIANT-4 study, the median PFS observed with everolimus were 9.2 and 13.6 months, respectively. OS data for RADIANT-4 were immature [43,44]. Recurrent MTC has a significant impact on survival rate, and current systemic treatments have limited effect on response and OS [18,45]. The tyrosine kinase inhibitors vandetanib and cabozantinib are considered first-line systemic options to treat progressive metastatic MTC [18,46]. The Phase III ZETA and EXAM studies in advanced MTC reported a median PFS of 30.5 months for vandetanib (predicted) and 11.2 months for cabozantinib, respectively [47,48]. The OS for cabozantinib in the EXAM study was 26.6 months [49]. The comparable survival outcomes of [^177^Lu]Lu-DOTA-TATE and standard therapies for advanced non-GEP-NETs highlight the potential clinical importance of [^177^Lu]Lu-DOTA-TATE as a treatment option for these patients with a poor prognosis.

In this review, safety data were not reported consistently by the studies. In some studies where efficacy was separated by NET subtype, the safety data were presented for the whole population, making it difficult to draw conclusions by NET subtype. However, in general, the mean cumulative dose of [^177^Lu]Lu-DOTA-TATE received by patients was similar and most AEs were of mild or moderate severity. In general, renal and bone marrow toxicities, when observed, were not clinically significant and the safety profiles observed were consistent with data from the NETTER-1 and ERASMUS trials [7,8,13]. A systematic review of nephrotoxicity after PRRT in different types of NETs concluded that there was a greater risk of long-term kidney damage with 90Y-labeled SSA analogs compared with those labeled with ^177^Lu [50]. Overall, in our analysis, nephrotoxicity severity, when reported, was low, but the studies varied considerably in the reporting of results and in the length of follow-up. Similarly, the reporting of secondary hematologic malignancies was infrequent, but the follow-up periods could have been too short to identify cases. A recent evaluation of treatment-related myeloid neoplasms in patients with NETs after PRRT concluded that the risk was low, but that patients should be monitored closely [51].

The initial search strategy for this review included thymic NETs. Only one retrospective case series assessing [^177^Lu]Lu-DOTA-TATE in 27 patients with mediastinal NETs was identified, but it failed to meet the inclusion criteria for our review because pediatric cases were included and efficacy data for thymic and mediastinal tumors were presented together [52]. The scarcity of data indicates that further studies in thymic NETs may be warranted to understand the potential clinical benefit of [^177^Lu]Lu-DOTA-TATE in these patients.

For this review, the search strategy did not have any language or time limits and was conducted by two independent reviewers to avoid selection bias. Many of the included studies were retrospective in design, with inherent possible selection and detection biases regarding the cases included in the analyses. The studies did report similar cumulative doses, and most studies used the RECIST 1.1 criteria to assess response. Limitations of this systematic literature review include the single database search, absence of data from randomized clinical trials, heterogeneous study designs, limited patient numbers per study, different follow-up periods, and the inconsistent reporting of data across the studies regarding baseline characteristics, treatment, treatment outcomes, and safety. Despite the heterogenous nature of the studies, the results indicate that the efficacy of [^177^Lu]Lu-DOTA-TATE in adult patients with metastatic, progressive, SSTR-positive PPGL, bronchial NETs, NETs of unknown primary origin, and MTC was consistent with the NETTER-1 and ERASMUS studies. These data support the inclusion of [^177^Lu]Lu-DOTA-TATE as a treatment option for non-GEP-NETs, as outlined in NCCN and ESMO guidelines [16,17,18,19].

## 5. Conclusions

This systematic literature review has shown that [^177^Lu]Lu-DOTA-TATE is used in clinical practice for the treatment of patients with PPGL, bronchial NETs, NETs of unknown primary origin, and MTC. When reported, the cumulative administered dose in the studies was mostly similar to that approved for [^177^Lu]Lu-DOTA-TATE in GEP-NET patients (i.e., 29.6 GBq). The results of this review support that [^177^Lu]Lu-DOTA-TATE offers encouraging antitumor activity in terms of objective responses, PFS, and OS outcomes in patients with poor prognosis. In addition, treatment with [^177^Lu]Lu-DOTA-TATE in patients with these tumor types showed a favorable safety profile, consistent with the safety profile reported for GEP-NET patients.

## Figures and Tables

**Table 1:** Study, patient, and treatment characteristics of included studies.

Study ID	Design and Country	Study Timeline	No. of Patients	Age, Years and Sex	Previous Treatment	NET Grade	NET Stage/Disease State at Baseline	SSTR Assessment Method and Grade	Per Cycle Activity of [^177^Lu]Lu-DOTA-TATE, GBq ^a^ (No. of Cycles)	Cumulative Administered Activity, GBq
PPGL										
Van Essen 2006 [22]	Prospective study Netherlands	Not specified	N = 12 PGL: *n* = 12	Mean 39.7 (range 22–55) M: *n* = 6 F: *n* = 6	Surgery: *n* = 9 Radiotherapy: *n* = 7 Chemotherapy: *n* = 4 None: *n* = 2	Not specified	Metastatic: *n* = 9 PD: *n* = 4 SD: *n* = 5 Unknown: *n* = 3	SSTR scintigraphy (OctreoScan): Grade 2: *n* = 1 Grade 3: *n* = 7 Grade 4: *n* = 4	7.4 (max. 4 cycles)	Range 14.8–29.6
Vyakaranam 2019 [23]	Retrospective cohort analysis (chart review) Sweden	2005–2018	N = 22 PCC: *n* = 9 PGL: *n* = 13	Median 60 (range 24–80) M: *n* = 13 F: *n* = 9	Surgery: *n* = 16 Radiotherapy: *n* = 14 [^131^I]I-MIBG: *n* = 6 Chemotherapy: *n* = 1	Median Ki-67, 11% (range 1–30) *n* = 18	Metastatic: *n* = 20 PD: *n* = 9	SSTR scintigraphy Krenning score ≥ 3	7.4 (range 3–11 cycles including salvage therapy)	Median 29.6 Range 22.2–81.4 (including salvage therapy)
Zandee 2019 [24]	Retrospective case series Netherlands	From Jan 2000	N = 30 PCC: *n* = 3 PGL: *n* = 27	Median 47 (range 29–74) M: *n* = 10 F: *n* = 20	Surgery: *n* = 19 Radiotherapy: *n* = 6 Chemotherapy: *n* = 5 [^131^I]I-MIBG: *n* = 3 SSA: *n* = 2	Not specified	Metastatic: *n* = 17 Localized: *n* = 13 ^b^ PD: *n* = 20 SD: *n* = 7 Unknown: *n* = 3	SSTR scintigraphy: Grade 2: *n* = 11 Grade 3: *n* = 13 Grade 4: *n* = 6	7.4 (range 1–4 cycles)	Range 14.8–29.6
Jaiswal 2020 [25]	Retrospective case series India	Jan 2010–Dec 2019	N = 14 PCC: *n* = 3 PGL: *n* = 10 PCC + PGL: n= 1	Range 18–59 M: *n* = 6 ^c^ F: *n* = 8 ^c^	Surgery: *n* = 9 EBRT: *n* = 3	Not specified	Progressive, metastasis: *n* = 6 Progressive, inoperable: *n* = 1 Inoperable + metastasis: *n* = 4 Inoperable: *n* = 3	PET/CT with ^68^Ga-labeled analog Krenning score ≥2: Score 2: *n* = 1 Score 3: *n* = 2 Score 4: *n* = 10 unknown: *n* = 1	5.55–7.4 (range 1–6 cycles)	Range 6–40
Parghane 2021 [26]	Retrospective case series India	2012–2019	N = 9 PGL: *n* = 9	Median 49 (range 33–61) M: *n* = 5 ^d^ F: *n* = 5	Surgery: *n* = 6 EBRT: *n* = 6 Chemotherapy: *n* = 1	Median Ki-67 19% (range 1–40): *n* = 5	Metastatic: *n* = 9 PD: *n* = 7	PET/CT with [^68^Ga]Ga-DOTA-TATE Krenning score ≥ 2	5.55–7.4 (range 1–6 cycles)	Average 24.42 Range 7.4–37
Roll 2020 [27]	Retrospective case series Germany	May 2014–Oct 2016	N = 6 PGL: *n* = 6 ^e^	Range 50–84 M: *n* = 2 ^e^ F: *n* = 4 ^e^	Surgery: *n* = 5 Embolization: *n* = 4 Fractionated photon irradiation: *n* = 1	Not specified	Metastatic: *n* = 0	PET/CT with [^68^Ga]Ga-DOTA-TATE Uptake higher than liver uptake on initial treatment	Mean 7.2 ± 0.4 (range 3–5 cycles)	Not specified
Pinato 2016 [21]	Retrospective case series UK	2008–2014	N = 4 PGL: *n* = 4	Range 29–47 M: *n* = 3 F: *n* = 1	Surgery: *n* = 3 Etoposide/cisplatin: *n* = 1 EBRT: *n* = 1 MIBG: *n* = 1	Not specified	Metastatic: *n* = 4 PD: *n* = 4	PET/CT with [^68^Ga]Ga-DOTA-TATE	6.6–7.5 (1 patient unknown dose) (range 1–4 cycles)	Not specified
Bronchial NETs										
Ianniello 2016 [28]	Prospective cohort study Italy	Apr 2008–Mar 2014	N = 34	Range 40–79 M: *n* = 17 F: *n* = 17	SSA: *n* = 29 Surgery: *n* = 22 Chemotherapy: *n* = 13 PRRT: *n* = 9 Other treatments: *n* = 9 None: *n* = 2	Typical: *n* = 15 Atypical: *n* = 19	Metastatic: *n* = 34 PD: *n* = 34	SSTR scintigraphy (OctreoScan) Krenning score ≥2: score 2: *n* = 14 score 3: *n* = 20	3.7 or 5.5 according to kidney and bone marrow toxicity risk factors (4 or 5 cycles)	Median 21.5 Range 12.9–27.8
Van Essen 2007 [29]	Prospective cohort study Netherlands	Not specified	N = 9	Median 62 (range 37–75) M: *n* = 6 F: *n* = 3	Surgery: *n* = 8 Radiotherapy: *n* = 2 Chemotherapy: *n* = 1	Typical: *n* = 4 Atypical: *n* = 5	Metastatic: *n* = 9 PD: *n* = 2 SD: *n* = 2	SSTR scintigraphy (OctreoScan): Grade 3: *n* = 8 Grade 4: *n* = 1	7.4 (3 or 4 cycles)	Intended cumulative dose: 22.2–29.6
Garske-Román 2018 [30]	Prospective cohort study Sweden	Enrollment Sep 2010–Feb 2014; Sweden/Oslo patients’ survival data from health registries accessed until May 2016	N = 6	Median 69 (range 41–75) M: *n* = 5 F: *n* = 1	Surgery: 33.3% Chemotherapy: 66.7% SSA: 20% Radiotherapy: 16.7%	Grade 1: *n* = 1 Grade 2: *n* = 5	Metastatic: *n* = 6 PD: *n* = 4 Extensive disease: *n* = 2	SSTR scintigraphy Krenning score ≥ 3	7.4 (5–8 cycles)	Not specified
Mariniello 2016 [31]	Retrospective cohort analysis Italy	Treated from 1997 to 2012 and followed until Oct 2014	N = 48	Mean (standard deviation): 61.5 (14.3) M: *n* = 32 F: *n* = 16	Surgery: *n* = 34 Chemotherapy: *n* = 18	Typical: *n* = 22, Atypical: *n* = 15 Not specified: *n* = 11	Advanced (unresectable/metastatic; stage IIIb/IV) PD: *n* = 39 Stage IV disease: *n* = 44	PET/CT with ^68^Ga-labeled analog or conventional OctreoScan	Planned cumulative dose of 27.75 (administered across 5 cycles) or 29.6 (8 cycles); 22.2 (6 cycles) if risk factors for delayed toxicity	Mean (standard deviation): 20.87 (7.78)
Mirvis 2020 [32]	Retrospective cohort analysis UK	2009–2020	N = 18	Not specified for [^177^Lu]Lu-DOTA-TATE-only patients	Not specified for [^177^Lu]Lu-DOTA-TATE-only patients	Moderate to well differentiated: *n* = 18	Advanced metastatic	[^111^In]In-DTPA- octreotide or ^68^Ga-SSA PET/CT Krenning score ≥ 2	~7.4 (range 2–4 cycles)	Median 29.8 Range 13.00–30.34
Sabet 2017 [33]	Retrospective cohort analysis Germany/ Austria	Not specified	N = 22	Mean 63 (range 42–74) M: *n* = 16 F: *n* = 6	Biotherapy: *n* = 16 Surgery: *n* = 14 Chemotherapy: *n* = 7 Locoregional treatment: *n* = 1	Ki-67 index: ≤2%: *n* = 9 3–20%: *n* = 13 Typical: *n* = 5 Atypical: *n* = 17	Metastatic, unresectable stage IV disease: *n* = 22 PD: *n* = 17	SSTR scintigraphy (e.g., OctreoScan) or PET imaging with ^68^Ga-SSA Uptake ≥ liver uptake	Mean 7.8 ± 0.68 (range 1–4 cycles)	Mean 27.2 ± 5.9
NETs of unknown origin										
Garske-Román 2018 [30]	Prospective cohort study Sweden	Enrollment Sep 2010–Feb 2014; Sweden/Oslo patients’ survival data from health registries accessed until May 2016	N = 8	Median 65 (range 54–80) M: *n* = 4 F: *n* = 4	Surgery (any type—not primary): 12.5% SSA: 62.5% Radiotherapy: 37.5% Chemotherapy: 87.5%	Grade 1: *n* = 1 Grade 2: *n* = 7	Metastatic: *n* = 8 PD: *n* = 6 Extensive disease: *n* = 7	SSTR scintigraphy Krenning score ≥ 3	7.4 (2–7 cycles)	Not specified for NET type
MTC										
Vaisman 2015 [34]	Prospective study Brazil	Jan 2011–Jul 2013	N = 9	Median 35.8 (range 20–54) M: *n* = 3 F: *n* = 6	Not reported	Not specified	Progressive MTC: *n* = 9	^111^In-DTPA- octreotide scintigraphy Any uptake	7.4 (up to 4 cycles)	Intended 29.6
Parghane 2020 [35]	Retrospective case series India	Jan 2012–Jul 2018	N = 43	Median 48 (range 25–80) M: *n* = 35 F: *n* = 8	Total thyroidectomy: *n* = 43 EBRT: *n* = 12 Chemotherapy (sorafenib): *n* = 1	Not specified	Progressive ^c^, metastatic MTC: ≥2 organ involvement: *n* = 34 Widespread metastatic disease: *n* = 17	PET/CT with [^68^Ga]Ga-DOTA-TATE Krenning score ≥ 2	5.55 Average 3 cycles (range 1–6)	Average 18.5 Range 5.55–33.3
Beukhof 2019 [36]	Retrospective case report or case series Netherlands	2000–2017	N = 10	Median 62 (range 19–75) M: *n* = 4 F: *n* = 6	Not specified	Not specified	Metastatic MTC: *n* = 10 PD: *n* = 8	^111^In-DTPA- octreotide scintigraphy and retrospective IHC	Not specified Average 4 cycles	Up to 27.8–29.6

^a^ Where activity was reported in mCi, it has been converted to GBq (1 mCi = 0.037 GBq). ^b^ Of these patients, nine had a PGL with multiple localization. ^c^ One male patient and one female patient had concomitant pNET. ^d^ One patient died before treatment started. ^e^ Includes one patient aged <18 years (this patient was excluded wherever possible). Abbreviations: CT—computed tomography; EBRT—external beam radiotherapy; F—female; IHC—immunohistochemistry; M—male; MIBG—meta-iodo-benzyl-guanidine; MTC—medullary thyroid carcinoma; NET— neuroendocrine tumor; PCC—pheochromocytoma; PD—progressive disease; PET—positron emission tomography; PGL—paraganglioma; pNET—pancreatic NET; PPGL—pheochromocytoma/paraganglioma; PRRT—peptide receptor radionuclide therapy; SD—stable disease; SSA—somatostatin analog; SSTR—somatostatin receptor.

**Table 2:** Study evaluation and outcomes of included studies.

Study ID	No. of Patients Treated	Follow-Up, Months	Response Criteria	Imaging Method for Response Evaluation and Time Points	Tumor Response: n (%)	ORR ^a^	DCR ^b^	PFS/OS
PPGL								
van Essen 2006 [22]	12	Median 13 (range 4–30)	SWOG	CT or MRI Measured at 6–8 weeks, 3 mo and 6 mo after last treatment, within every 6 mo thereafter	PR: 1 (8) MR: 1 (8) SD: 6 (50) PD: 3 (25) No data: 1 (8)	8%	67%	TTP ^c^: Median could not be determined (11 and 15 mo in 2 patients) OS: Not reported
Vyakaranam 2019 [23]	22	Median 32 (range 8–139)	RECIST 1.1	CT/MRI Measured before every second treatment cycle, 3 mo after last treatment, at least every 6 mo thereafter	PR: 2 (9) SD: 20 (91)	9%	100%	Median PFS: 21.6 mo (range 6.7–138) Median OS: 49.6 mo (range 8.2–139)
Zandee 2019 [24]	30	Median 52.5 ^d^ (range 7–155)	RECIST 1.1	Radiographic tumor assessment	*All (N = 30)* PR: 7 (23) SD: 20 (67) PD: 3 (10) *pPGL (n = 17): * PR: 2 (12) SD: 14 (82) PD: 1 (6) *sPGL (n = 10)* PR: 4 (40) SD: 5 (50) PD: 1 (10) *PCC (n = 3): * PR: 1 (33) SD: 1 (33) PD: 1 (33)	23% 12% 40% 33%	90% 94% 90% 67%	Median PFS: 30 mo ^d^ Median OS: NR ^d^ Median PFS: 91 mo ^d^ Median OS: NR ^d^ Median PFS: 18 mo ^d^ Median OS: 59 mo ^d^ Median PFS: 10 mo ^d^ Median OS: 17 mo ^d^
Jaiswal 2020 [25]	14	Range 11–62	RECIST 1.1 with MR	CeCT	*All (N = 14)* PR: 1 (7) MR: 4 (29) SD: 7 (50) ^e^ PD: 2 (14) *PGL (n = 10): * PR: 1 (10) MR: 4 (40) SD: 5 (50) *PCC (n = 2):* PD: 2 (100) *PCC + pNET (n = 1):* SD: 1 (100) *PCC + pNET + PGL (n = 1):* SD: 1 (100)	7% 10% 0% 0% 0%	86% 100% 0% 100% 100%	Median PFS: NR
Parghane 2021 [26]	9	Median 40	RECIST 1.1 with MR	CeCT or diagnostic CT part of PET-CT scan Measured before each PRRT cycle (at 10–12 weeks) and then every 6 mo after completing cycles	CR: 0 PR: 1 (11) MR: 2 (22) SD: 3 (33) PD: 3 (33)	11%	67%	Median PFS: NR Median OS: NR PFS rate: 63% (95% CI 30–96) Estimated OS rate: 65% (95% CI 32–97) at 40 mo
Roll 2020 [27]	6	Median 39 (range 16–64)	RECIST 1.1	[^68^Ga]Ga-DOTA-TATE PET and CeCT or MRI Measured 3 mo after the last treatment cycle	SD: 6 (100)	0%	100%	Not reported
Pinato 2016 [21]	4	Range 26–84	Not specified	CT and PET Measured following each cycle	PR: 1 (25) SD: 2 (50) PD: 1 (25)	25%	75%	Median OS: NR (range 26–84 mo) Mean (standard deviation) OS: 53 (22.7) mo Mean (standard deviation) PFS: 36.4 (27.4) mo (range 1–78)
Bronchial NETs								
Ianniello 2016 [28]	34	Median 29 (range 7–69)	SWOG	Multiphase CT and/or MRI Measured at 3, 6, 12, 18, and 24 mo after treatment and every 6–12 mo thereafter	*All (N = 34)* CR: 1 (3) PR: 4 (12) SD: 16 (47) *Typical (n = 15)* CR: 1 (7) PR: 4 (27) SD: 7 (47) *Atypical (n = 19)* SD: 9 (47)	15% 33% 0%	62% 80% 47%	Median PFS: 18.5 mo (95% CI 12.9–26.4) Median OS: 48.6 mo (95% CI 26.4–68.9) Median PFS: 20.1 mo (95% CI 11.8–26.8) Median OS: 48.6 mo (95% CI 26.0–NR) Median PFS: 15.7 mo (95% CI 10.6–25.9) Median OS: 37 mo (95% CI 18.7–68.9)
van Essen 2007 [29]	9	Median 36 (range 23–76)	Modified SWOG	CT or MRI Measured at 6–8 weeks, 3 mo, and 6 mo after last treatment, and every 6 mo thereafter	PR: 5 (56) MR: 1 (11) SD: 2 (22) PD: 1 (11)	56%	89%	Median TTP ^b^: 31 mo
Garske-Román 2018 [30]	6	Not specified	RECIST 1.1	Radiological assessment Scintigraphy or ultrasonography used in clinically clear cases of progression when CT data were not available	PR: 1 (17) SD: 5 (83)	17%	100%	Median PFS: 18 mo (95% CI 12–43) Median OS: NR (19 mo–NR)
Mariniello 2016 [31]	48	Median 45.1 (range 3–191)	RECIST	CT, MRI Measured at 6–8 weeks after the second cycle and every 6 or 12 mo thereafter	PR: 6 (13) MR: 8 (17) SD: 22 (46)	13%	75%	Median PFS: 31.0 mo (IQR 21.0–49.1) PFS at 3 y after the start of PRRT: 39.8% (95% CI 0.25–0.54) 5-y OS: 61.4% (95% CI 41.5–77.0) Median OS: NR at 110 mo
Mirvis 2020 [32]	18	Not reported	RECIST 1.1	CT	Not reported	NA	NA	Median PFS: 18 mo
Sabet 2017 [33]	22	Median 54 (range 5–75)	RECIST 1.1	CT or MRI Measured at 3 mo after termination of PRRT and every 6 months thereafter	PR: 6 (27) SD: 9 (41) PD: 7 (32)	27%	68%	Median PFS: 27 mo (95% CI 9–45) Median OS: 42 mo (95% CI 25–59)
NETs of unknown origin								
Garske-Román 2018 [30]	8	Not specified	RECIST 1.1	Radiological assessment Scintigraphy or ultrasonography used in clinically clear cases of progression when CT data were not available	PR: 3 (38) SD: 4 (50) PD: 1 (13)	38%	88%	Median PFS: 17.5 mo (95% CI 7–34) Median OS: 43 mo (95% CI 15–NR)
MTC								
Vaisman 2015 [34]	7 ^f^	Range 8–12 months	RECIST 1.1	CT scans of the neck and chest and MRI of the liver and known bone metastasis Measured at 8–12 months after finishing 4th cycle	PR: 3 (43) SD: 3 (43) PD: 1 (14)	43%	86%	Not reported
Parghane 2020 [35]	43	Median (range) 20 (8–78)	RECIST 1.1	CeCT or CT part of PET-CT scan	PR: 2 (5) SD: 25 (58) PD: 16 (37)	5%	63%	Median PFS: 24 mo (95% CI 15.1–32.9) Median OS: 26 mo (95% CI 16.6–35.3)
Beukhof 2019 [36]	10	Not specified	RECIST 1.1	Not specified Measured at 3 months after completing treatment	SD: 4 (40) PD: 6 (60)	0%	40%	Median PFS: 0.7 y (range 0.3–12.0) Median OS: 1.14 y (range 0.4–12.0) Median OS in SD patients: 1.8 y (range 0.8–12.0)

^a^ Calculated from data presented (CR + PR/N). ^b^ Calculated from data presented (CR + PR + MR + SD/N). ^c^ Time to progression used instead of PFS. ^d^ In patients with baseline PD. ^e^ Two patients with SD also had pNET. ^f^ Of the nine patients eligible for treatment, one died before starting treatment and one refused to participate, leaving seven evaluable patients. Abbreviations: CeCT—contrast-enhanced computed tomography; CI—confidence interval; CR—complete response; CT—computed tomography; DCR—disease control rate; IQR—interquartile range; mo—months; MR—minor response; MRI—magnetic resonance imaging, MTC—medullary thyroid carcinoma, NA—not available, NET—neuroendocrine tumor, NR—not reached, ORR—objective response rate, OS—overall survival; PCC—pheochromocytoma; PD—progressive disease; PET—positron emission tomography; PFS—progression-free survival; PGL—paraganglioma; pNET—pancreatic NET; PPGL—pheochromocytoma/paraganglioma; pPGL—parasympathetic paraganglioma; PR—partial response; PRRT—peptide receptor radionuclide therapy; RECIST—response evaluation criteria in solid tumors; SD—stable disease; sPGL—sympathetic paraganglioma; SWOG—Southwest Oncology Group; TTP—time to tumor progression; y—years.

**Table 3:** [^177^Lu]Lu-DOTA-TATE safety summary (all patients).

Study ID	N	Indication	Acute Toxicity Per Treatment, %	AEs, n (%)	Renal Toxicity, n (%)	Hematologic Toxicity, n (%)	Additional Comments
Vyakaranam 2019 [23]	22	PPGL	Not reported	Not reported	None	None: 6 (27) ^a^ Any grade 1/2: 16 (73) ^a^	
Zandee 2019 [24]	30	PPGL	Nausea: 34% Pain: 23% Vomiting: 13%	Cardiac failure: 1 (3) Pleural effusion and delirium: 1 (3)	Not reported	Anemia grade 3: 2 (7) ^b^ Thrombocytopenia grade 3: 4 (13) ^b^ Thrombocytopenia grade 4: 1 (3) ^b^ Leukopenia grade 3: 3 (10) ^b^	MDS: 1 (considered to be related to treatment) Persistent thrombocytopenia limited treatment in 3 patients
Jaiswal 2020 [25]	15 ^c^	PPGL	Not reported	Nausea/vomiting: 3 (20) Weight loss: 2 (13)	None	Thrombocytopenia grade 2: 1 (7) ^d^ Anemia + thrombocytopenia grade 2: 1 (7) ^d^	
Parghane 2021 [26]	9	PGL	Not reported	Nausea/vomiting: 2 (22)	None	Anemia grade 1: 1 (11) Thrombocytopenia: 0 Leukopenia: 0	
Roll 2020 [27]	7 ^e^	PGL	Not reported	Not reported	Not reported	None: 4 (57) Leukopenia grade 1: 1 (14) Anemia grade 1: 1 (14) Leukopenia grade 2 + anemia grade 1: 1 (14)	
Pinato 2016 [21]	5 ^f^	PGL	None	Suspected pneumonitis: 1 (20) Reactionary swelling of metastases: 1 (20)	Not reported	Not reported	
Ianniello 2016 [28]	34	Bronchial NETs	No grade ≥3 ^b^	Not reported	Not reported	Any grade ≥3: 0 ^b^	
Mariniello 2016 [31]	47 ^g^	Bronchial NETs	Not reported	Not reported	Serum creatinine increase grade 0: 34 (74) ^a^ Serum creatinine increase grade 1: 11 (23) ^a^ Serum creatinine increase grade 2: 1 (2) ^a^	Anemia grade 0: 12 (26) ^a^ Anemia grade 1: 32 (68) ^a^ Anemia grade 2: 3 (6) ^a^ Leukopenia Grade 0: 28 (60) ^a^ Leukopenia grade 1: 14 (30) ^a^ Leukopenia grade 2: 5 (11) ^a^ Thrombocytopenia grade 0: 28 (60) ^a^ Thrombocytopenia grade 1: 18 (38) ^a^ Thrombocytopenia grade 2: 1 (2) ^a^	MDS: 0 AML: 0
Mirvis 2020 [32]	18	Bronchial NETs	Not reported	Radiation pericarditis grade 3: 1 (6)	None	Thrombocytopenia grade 3: 1 (6) ^a^	MDS: 0 Leukemia: 0
Sabet 2017 [33]	22	Bronchial NETs	No serious events	Not reported	Any grade ≥3: 0 ^b^	Any grade 3: 3 (14) ^b^	
Vaisman 2015 [34]	7 ^h^	MTC	Not reported	Sexual dysfunction: 1 (14) Hair loss: 2 (29) Hypersensitivity: 1 (14) Any grade ≥3: 0 ^b^	None	None	
Parghane 2020 [35]	43	MTC	Not reported	Nausea grade 1: 1 (2) Any grade ≥3: 0	None	Any grade 1: 1 (2)	
Beukhof 2019 [36]	10	MTC	Not reported	Diarrhea Grade 2: 1 (10) ^a^ Hemoptysis Grade 3: 1 (10) ^a^	Not reported	Not reported	

Toxicity was reported using the Common Terminology Criteria for Adverse Events (CTCAE) Version 5.0 unless otherwise stated. ^a^ CTCAE version 4.0. ^b^ CTCAE version 3.0. ^c^ One adolescent patient of 14 years with PCC + pNET was included in the safety data. ^d^ Criteria used for grading AEs not specified. ^e^ One adolescent patient of 14 years with PGL was included in the safety data. ^f^ One adolescent patient of 16 years with PGL was included in the safety data. ^g^ One patient was lost to follow-up prior to toxicity assessments. ^h^ One patient died before treatment and one refused to participate in the study. Abbreviations: AE—adverse event; AML—acute myeloid leukemia; MDS—myelodysplastic syndrome; MTC—medullary thyroid carcinoma; NET—neuroendocrine tumor; PCC—pheochromocytoma; PGL—paraganglioma; pNET—pancreatic NET; PPGL—pheochromocytoma/paraganglioma.

## Data Availability

Not applicable.

## Supplementary Materials

The following supporting information can be downloaded at: https://www.mdpi.com/article/10.3390/biomedicines11041024/s1, Supplementary Materials S1: PubMed search strategy; Supplementary Materials S2: Excluded records.
