# Supplementary material for: Efficacy and Safety of [177Lu]Lu-DOTA-TATE in Adults with Inoperable or Metastatic Somatostatin Receptor-Positive Pheochromocytomas/Paragangliomas, Bronchial and Unknown Origin Neuroendocrine Tumors, and Medullary Thyroid Carcinoma: A Systematic Literature Review
Source: Biomedicines. 2023 Mar 27;11(4):1024. doi: 10.3390/biomedicines11041024 (PMC10135775; doi:10.3390/biomedicines11041024)
Supplement: Supplementary file 1 [file biomedicines-11-01024-s001.zip › biomedicines-2271409-supplementary.pdf]

Supplementary Materials

Efficacy and Safety of [^{177}Lu]Lu-DOTA-TATE in Adults with Inoperable or Metastatic Somatostatin Receptor-Positive Pheochromocytomas/Paragangliomas, Bronchial and Unknown Origin Neuroendocrine Tumors, and Medullary Thyroid Carcinoma: A Systematic Literature Review

Marianna Hertelendi, Oulaya Belguenani, Azzeddine Cherfi, Ilya Folitar, Gabor Kollar and Berna Degirmenci Polack

Supplementary Materials S1: PubMed search strategy 2
Supplementary Materials S2: Excluded records 5
Excluded records from the first screen (654 excluded of 1057 screened) 5
Excluded records following assessment for eligibility (385 excluded of 403 assessed), by exclusion reason 34
Non-separable data between different NETs (n=85) 34
Did not report outcome of interest (n=70) 38
Review paper (n=61) 41
Non-target NET type (n=48) 43
Non-separable treatment (n=29) 45
Case report or only individual data reported (n=28) 47
Not treated with [^{177}Lu]Lu-DOTA-TATE (n=27) 48
Did not report specific NET type (n=21) 49
[^{177}Lu]Lu-DOTA-TATE used in tandem/combination with another anticancer agent (n=9) 50
PRRT product not specified (n=3) 51
Non-English text (n=3) 51
Non-separable data between adults and pediatrics (n=1) 51
Duplicated record (n=1) 51

Supplementary Materials S1: PubMed search strategy

PubMed search date: May 13, 2021

Search for NETs: Number of hits=203,645

(("medulla oblongata"[MeSH Terms] OR ("medulla"[All Fields] AND "oblongata"[All Fields]) OR "medulla oblongata"[All Fields] OR "medullary"[All Fields] OR "adrenal medulla"[MeSH Terms] OR ("adrenal"[All Fields] AND "medulla"[All Fields]) OR "adrenal medulla"[All Fields]) AND ("thyroid neoplasms"[MeSH Terms] OR ("thyroid"[All Fields] AND "neoplasms"[All Fields]) OR "thyroid neoplasms"[All Fields] OR ("thyroid"[All Fields] AND "cancer"[All Fields]) OR "thyroid cancer"[All Fields])) OR (("medulla oblongata"[MeSH Terms] OR ("medulla"[All Fields] AND "oblongata"[All Fields]) OR "medulla oblongata"[All Fields] OR "medullary"[All Fields] OR "adrenal medulla"[MeSH Terms] OR ("adrenal"[All Fields] AND "medulla"[All Fields]) OR "adrenal medulla"[All Fields]) AND ("thyroid neoplasms"[MeSH Terms] OR ("thyroid"[All Fields] AND "neoplasms"[All Fields]) OR "thyroid neoplasms"[All Fields] OR ("thyroid"[All Fields] AND "carcinoma"[All Fields]) OR "thyroid carcinoma"[All Fields])) OR (("medulla oblongata"[MeSH Terms] OR ("medulla"[All Fields] AND "oblongata"[All Fields]) OR "medulla oblongata"[All Fields] OR "medullary"[All Fields] OR "adrenal medulla"[MeSH Terms] OR ("adrenal"[All Fields] AND "medulla"[All Fields]) OR "adrenal medulla"[All Fields]) AND ("thyroid neoplasms"[MeSH Terms] OR ("thyroid"[All Fields] AND "neoplasms"[All Fields]) OR "thyroid neoplasms"[All Fields] OR ("thyroid"[All Fields] AND "tumor"[All Fields]) OR "thyroid tumor"[All Fields])) OR (("medulla oblongata"[MeSH Terms] OR ("medulla"[All Fields] AND "oblongata"[All Fields]) OR "medulla oblongata"[All Fields] OR "medullary"[All Fields] OR "adrenal medulla"[MeSH Terms] OR ("adrenal"[All Fields] AND "medulla"[All Fields]) OR "adrenal medulla"[All Fields]) AND ("thyroid neoplasms"[MeSH Terms] OR ("thyroid"[All Fields] AND "neoplasms"[All Fields]) OR "thyroid neoplasms"[All Fields] OR ("thyroid"[All Fields] AND "tumour"[All Fields]) OR "thyroid tumour"[All Fields])) OR ("bronchial carcinoid"[All Fields] OR "bronchial neuroendocrine tumour"[All Fields] OR "bronchial neuroendocrine tumor"[All Fields] OR (("bronchial"[All Fields] OR "bronchiale"[All Fields] OR "bronchials"[All Fields]) AND ("neurology"[MeSH Terms] OR "neurology"[All Fields] OR "neuro"[All Fields] OR "neuros"[All Fields]) AND ("endocrine gland neoplasms"[MeSH Terms] OR ("endocrine"[All Fields] AND "gland"[All Fields] AND "neoplasms"[All Fields]) OR "endocrine gland neoplasms"[All Fields] OR ("endocrine"[All Fields] AND "tumour"[All Fields]) OR "endocrine tumour"[All Fields])) OR (("bronchial"[All Fields] OR "bronchiale"[All Fields] OR "bronchials"[All Fields]) AND ("neurology"[MeSH Terms] OR "neurology"[All Fields] OR "neuro"[All Fields] OR "neuros"[All Fields]) AND ("endocrine gland neoplasms"[MeSH Terms] OR ("endocrine"[All Fields] AND "gland"[All Fields] AND "neoplasms"[All Fields]) OR "endocrine gland neoplasms"[All Fields] OR ("endocrine"[All Fields] AND "tumor"[All Fields]) OR "endocrine tumor"[All Fields])) OR "pulmonary neuroendocrine tumor"[All Fields] OR "pulmonary neuroendocrine tumour"[All Fields] OR (("lung"[MeSH Terms] OR "lung"[All Fields] OR "pulmonary"[All Fields]) AND ("neurology"[MeSH Terms] OR "neurology"[All Fields] OR "neuro"[All Fields] OR "neuros"[All Fields]) AND ("endocrine gland neoplasms"[MeSH Terms] OR ("endocrine"[All Fields] AND "gland"[All Fields] AND "neoplasms"[All Fields]) OR "endocrine gland neoplasms"[All Fields] OR ("endocrine"[All Fields] AND "tumor"[All Fields]) OR "endocrine tumor"[All Fields])) OR (("lung"[MeSH Terms] OR "lung"[All Fields] OR "pulmonary"[All Fields]) AND ("neurology"[MeSH Terms] OR "neurology"[All Fields] OR "neuro"[All Fields] OR "neuros"[All Fields]) AND ("endocrine gland neoplasms"[MeSH Terms] OR ("endocrine"[All Fields] AND "gland"[All Fields] AND "neoplasms"[All Fields]) OR "endocrine gland neoplasms"[All Fields] OR ("endocrine"[All Fields] AND "tumour"[All Fields]) OR "endocrine tumour"[All Fields])) OR "pulmonary neuroendocrine carcinoma"[All Fields] OR (("lung"[MeSH Terms] OR "lung"[All Fields] OR "pulmonary"[All Fields]) AND ("neurology"[MeSH Terms] OR "neurology"[All Fields] OR "neuro"[All Fields] OR "neuros"[All Fields]) AND ("endocrinal"[All Fields] OR "endocrine system"[MeSH Terms] OR ("endocrine"[All Fields] AND "system"[All Fields]) OR "endocrine system"[All Fields] OR "endocrine"[All Fields] OR "endocrines"[All Fields] OR "endocrinic"[All Fields] OR "endocrinous"[All Fields]) AND ("carcinoma"[MeSH Terms] OR "carcinoma"[All Fields] OR "carcinomas"[All Fields] OR "carcinoma s"[All Fields])) OR "bronchopulmonary carcinoid"[All Fields] OR "broncho pulmonary carcinoid"[All Fields] OR "bronchopulmonary cancer"[All Fields] OR "broncho pulmonary cancer"[All Fields] OR "bronchopulmonary tumor"[All Fields] OR ("bronchopulmonary"[All Fields] AND ("cysts"[MeSH Terms] OR "cysts"[All Fields] OR "cyst"[All Fields] OR "neurofibroma"[MeSH Terms] OR "neurofibroma"[All Fields] OR "neurofibromas"[All Fields] OR "tumor s"[All Fields] OR "tumoral"[All Fields] OR "tumorous"[All Fields] OR "tumour"[All Fields] OR "neoplasms"[MeSH Terms] OR "neoplasms"[All Fields] OR "tumor"[All Fields] OR "tumour s"[All Fields] OR "tumoural"[All Fields] OR "tumourous"[All Fields] OR "tumours"[All Fields] OR "tumors"[All Fields])) OR ("broncho"[All Fields] AND ("lung"[MeSH Terms] OR "lung"[All Fields] OR "pulmonary"[All Fields]) AND ("cysts"[MeSH Terms] OR "cysts"[All Fields] OR "cyst"[All Fields] OR "neurofibroma"[MeSH Terms] OR

"neurofibroma"[All Fields] OR "neurofibromas"[All Fields] OR "tumor s"[All Fields] OR "tumoral"[All Fields] OR "tumorous"[All Fields] OR "tumour"[All Fields] OR "neoplasms"[MeSH Terms] OR "neoplasms"[All Fields] OR "tumor"[All Fields] OR "tumour s"[All Fields] OR "tumoural"[All Fields] OR "tumourous"[All Fields] OR "tumours"[All Fields] OR "tumors"[All Fields])) OR ("broncho"[All Fields] AND ("lung"[MeSH Terms] OR "lung"[All Fields] OR "pulmonary"[All Fields]) AND ("cysts"[MeSH Terms] OR "cysts"[All Fields] OR "cyst"[All Fields] OR "neurofibroma"[MeSH Terms] OR "neurofibroma"[All Fields] OR "neurofibromas"[All Fields] OR "tumor s"[All Fields] OR "tumoral"[All Fields] OR "tumorous"[All Fields] OR "tumour"[All Fields] OR "neoplasms"[MeSH Terms] OR "neoplasms"[All Fields] OR "tumor"[All Fields] OR "tumour s"[All Fields] OR "tumoural"[All Fields] OR "tumourous"[All Fields] OR "tumours"[All Fields] OR "tumors"[All Fields]))) OR ("thymic neuroendocrine tumor"[All Fields] OR "thymic neuroendocrine tumour"[All Fields] OR "thymic neuroendocrine carcinoma"[All Fields] OR (("thymically"[All Fields] OR "thymus gland"[MeSH Terms] OR ("thymus"[All Fields] AND "gland"[All Fields]) OR "thymus gland"[All Fields] OR "thymic"[All Fields]) AND ("carcinoma, neuroendocrine"[MeSH Terms] OR ("carcinoma"[All Fields] AND "neuroendocrine"[All Fields]) OR "neuroendocrine carcinoma"[All Fields] OR ("neuroendocrine"[All Fields] AND "cancer"[All Fields]) OR "neuroendocrine cancer"[All Fields]))) OR ("paraganglioma"[MeSH Terms] OR "paraganglioma"[All Fields] OR "paragangliomas"[All Fields] OR ("phaeochromocytoma"[All Fields] OR "pheochromocytoma"[MeSH Terms] OR "pheochromocytoma"[All Fields] OR "phaeochromocytomas"[All Fields] OR "pheochromocytomas"[All Fields])) OR ("unknown primary"[All Fields] AND ((("neurology"[MeSH Terms] OR "neurology"[All Fields] OR "neuro"[All Fields] OR "neuros"[All Fields]) AND ("endocrine gland neoplasms"[MeSH Terms] OR ("endocrine"[All Fields] AND "gland"[All Fields] AND "neoplasms"[All Fields]) OR "endocrine gland neoplasms"[All Fields] OR ("endocrine"[All Fields] AND "tumour"[All Fields]) OR "endocrine tumour"[All Fields])) OR ("neuroendocrine tumour"[All Fields] OR "neuroendocrine tumors"[MeSH Terms] OR ("neuroendocrine"[All Fields] AND "tumors"[All Fields]) OR "neuroendocrine tumors"[All Fields] OR ("neuroendocrine"[All Fields] AND "tumor"[All Fields]) OR "neuroendocrine tumor"[All Fields]))) OR ((("neurology"[MeSH Terms] OR "neurology"[All Fields] OR "neuro"[All Fields] OR "neuros"[All Fields]) AND ("endocrine gland neoplasms"[MeSH Terms] OR ("endocrine"[All Fields] AND "gland"[All Fields] AND "neoplasms"[All Fields]) OR "endocrine gland neoplasms"[All Fields] OR ("endocrine"[All Fields] AND "tumour"[All Fields]) OR "endocrine tumour"[All Fields])) OR ("neuroendocrine tumour"[All Fields] OR "neuroendocrine tumors"[MeSH Terms] OR ("neuroendocrine"[All Fields] AND "tumors"[All Fields]) OR "neuroendocrine tumors"[All Fields] OR ("neuroendocrine"[All Fields] AND "tumor"[All Fields]) OR "neuroendocrine tumor"[All Fields]))

Search for [^{177}Lu]Lu-DOTA-TATE: Number of hits=2932

"lutetium Lu 177 dotatate"[All Fields] OR ("Peptide receptor radionuclide therapy"[All Fields] OR "Peptide receptor radionuclide treatment"[All Fields]) OR ("lutetium Lu 177 dotatate"[Supplementary Concept] OR "lutetium Lu 177 dotatate"[All Fields] OR "lutathera"[All Fields] OR "lutetium"[MeSH Terms] OR "lutetium"[All Fields]) OR ("lutetium Lu 177 dotatate"[Supplementary Concept] OR "lutetium Lu 177 dotatate"[All Fields]) OR ("lutetium Lu 177 dotatate"[Supplementary Concept] OR "lutetium Lu 177 dotatate"[All Fields]) OR "177lu dotaotyr3 octreotate"[All Fields] OR ("lutetium Lu 177 dotatate"[Supplementary Concept] OR "lutetium Lu 177 dotatate"[All Fields] OR "dotatate 177lu"[All Fields]) OR ("lutetium Lu 177 dotatate"[Supplementary Concept] OR "lutetium Lu 177 dotatate"[All Fields] OR "177lu dotatate"[All Fields]) OR ((("177lutetium-DOTA"[All Fields] AND "O"[All Fields]) AND "Tyr3"[All Fields]) AND "octreotate"[All Fields]) OR "177Lu-DOTA-TATE"[All Fields]

Combined search: Number of hits=1057

Supplementary Materials S2: Excluded records

Excluded records from the first screen (654 excluded of 1057 screened)

General reasons for exclusion during first screen:

- Duplicated records
- Pediatric patients only
- Non-target NET type
- Non-English text
- No outcome data of interest
- Preclinical study
- Not treated with [^{177}Lu]Lu-DOTA-TATE
- [^{177}Lu]Lu-DOTA-TATE used in tandem/combination with another anticancer agent

1. NETTER-1 Phase III in patients with midgut neuroendocrine tumors treated with 177Lu-DOTATATE: efficacy and safety results. *Clin Adv Hematol Oncol* 2016;**14**(5 Suppl 7):8-9.
2. Lutetium lu 177 dotatate (Lutathera) for gastroenteropancreatic neuroendocrine tumors. *Med Lett Drugs Ther* 2018;**60**(1555):e152-e3.
3. Neuroendocrine neoplasia within the German NET Registry [article in German]. *Z Gastroenterol* 2018;**56**(10):1237-46.
4. Abbott A, Sakellis CG, Andersen E, et al. Guidance on (177)Lu-DOTATATE peptide receptor radionuclide therapy from the experience of a single nuclear medicine division. *J Nucl Med Technol* 2018;**46**(3):237-44.
5. Abdülrezzak Ü, Kula M, Tutuş A, Buyukkaya F, Karaca H. PET/CT and Bremsstrahlung imaging after 90Y DOTANOC therapy for rectal net with liver metastases. *Clin Nucl Med* 2015;**40**(10):799-801.
6. Abell SK, Teng J, Dowling A, Hofman MS, MacIsaac RJ, Sachithanandan N. Prolonged life-threatening hypoglycaemia following dose escalation of octreotide LAR in a patient with malignant polysecreting pancreatic neuroendocrine tumour. *Endocrinol Diabetes Metab Case Rep* 2015;**2015**:140097.
7. Adnan A, Basu S. Rare-site primary soft-tissue neuroendocrine tumor with metastases and near-complete resolution with (177)Lu-DOTATATE: documenting a promising clinical application of peptide receptor radionuclide therapy. *J Nucl Med Technol* 2020;**48**(1):36-9.
8. Ahlstedt J, Tran TA, Strand F, et al. Biodistribution and pharmacokinetics of recombinant α1-microglobulin and its potential use in radioprotection of kidneys. *Am J Nucl Med Mol Imaging* 2015;**5**(4):333-47.
9. Ahlstedt J, Tran TA, Strand SE, Gram M, Åkerström B. Human anti-oxidation protein A1M – a potential kidney protection agent in peptide receptor radionuclide therapy. *Int J Mol Sci* 2015;**16**(12):30309-20.
10. Albertelli M, Dotto A, Di Dato C, et al. PRRT: identikit of the perfect patient. *Rev Endocr Metab Disord* 2021;**22**(3):563-79.
11. Albrecht J, Exner S, Grötzinger C, et al. Multimodal imaging of 2-cycle PRRT with (177)Lu-DOTA-JR11 and (177)Lu-DOTATOC in an orthotopic neuroendocrine xenograft tumor mouse model. *J Nucl Med* 2021;**62**(3):393-8.
12. Alexander N, Vali R, Ahmadzadehfar H, Shammas A, Baruchel S. Review: The role of radiolabeled DOTA-conjugated peptides for imaging and treatment of childhood neuroblastoma. *Curr Radiopharm* 2018;**11**(1):14-21.
13. Alexandraki KI, Kaltsas G. Gastroenteropancreatic neuroendocrine tumors: new insights in the diagnosis and therapy. *Endocrine* 2012;**41**(1):40-52.
14. Aloj L, Aurilio M, Rinaldi V, et al. Comparison of the binding and internalization properties of 12 DOTA-coupled and ^{111}In-labelled CCK2/gastrin receptor binding peptides: a collaborative project under COST Action BM0607. *Eur J Nucl Med Mol Imaging* 2011;**38**(8):1417-25.
15. Aloj L, Giger O, Mendichovszky IA, et al. The role of [(68) Ga]Ga-DOTATATE PET/CT in wild-type KIT/PDGFRA gastrointestinal stromal tumours (GIST). *EJNMMI Res* 2021;**11**(1):5.
16. Alsadik S, Yusuf S, Al-Nahhas A. Peptide receptor radionuclide therapy for pancreatic neuroendocrine tumours. *Curr Radiopharm* 2019;**12**(2):126-34.
17. Alshaikh OM, Asa SL, Mete O, Ezzat S. An institutional experience of tumor progression to pituitary carcinoma in a 15-year cohort of 1055 consecutive pituitary neuroendocrine tumors. *Endocr Pathol* 2019;**30**(2):118-27.
18. Alshaikh OM, Yoon JY, Chan BA, et al. Pancreatic neuroendocrine tumor producing insulin and vasopressin. *Endocr Pathol* 2018;**29**(1):15-20.

19. Ambrosini V, Campana D, Bodei L, et al. 68Ga-DOTANOC PET/CT clinical impact in patients with neuroendocrine tumors. *J Nucl Med* 2010;**51**(5):669-73.

20. Ambrosini V, Campana D, Polverari G, et al. Prognostic value of 68Ga-DOTANOC PET/CT SUVmax in patients with neuroendocrine tumors of the pancreas. *J Nucl Med* 2015;**56**(12):1843-8.

21. Andersson CK, Shubbar E, Schüler E, Åkerström B, Gram M, Forssell-Aronsson EB. Recombinant α(1)-microglobulin is a potential kidney protector in (177)Lu-octreotate treatment of neuroendocrine tumors. *J Nucl Med* 2019;**60**(11):1600-4.

22. Andreasi V, Partelli S, Muffatti F, Manzoni MF, Capurso G, Falconi M. Update on gastroenteropancreatic neuroendocrine tumors. *Dig Liver Dis* 2021;**53**(2):171-82.

23. Apostolidis L, Nientiedt C, Winkler EC, et al. Clinical characteristics, treatment outcomes and potential novel therapeutic options for patients with neuroendocrine carcinoma of the prostate. *Oncotarget* 2019;**10**(1):17-29.

24. Arora G, Shukla J, Ghosh S, Maulik SK, Malhotra A, Bandopadhyaya G. PLGA nanoparticles for peptide receptor radionuclide therapy of neuroendocrine tumors: a novel approach towards reduction of renal radiation dose. *PLoS One* 2012;**7**(3):e34019.

25. Artiko V, Afgan A, Petrović J, et al. Evaluation of neuroendocrine tumors with 99mTc-EDDA/HYNIC TOC. *Nucl Med Rev Cent East Eur* 2016;**19**(2):99-103.

26. Arveschoug AK, Hjorthaug K, Rehling M, Højgaard L, Mortensen J, Oturai PS. Peptide receptor radionuclide therapy of neuroendocrine tumors [article in Danish]. *Ugeskr Laeger* 2009;**171**(13):1073.

27. Arveschoug AK, Kramer SM, Iversen P, Frøkiær J, Grønbæk H. Monitoring kidney function in neuroendocrine tumor patients treated with (90)Y-DOTATOC: associations with risk factors. *Curr Radiopharm* 2015;**8**(1):49-55.

28. Ashton CE, Jeans SP, Valle JW. Combination therapy of 177Lu and 90Y Dotatate for treatment of neuroendocrine cancer. *Nucl Med Commun* 2014;**35**(6):585-7.

29. Assadi M, Pirayesh E, Rekabpour SJ, et al. 177Lu-PSMA and 177Lu-DOTATATE therapy in a patient with metastatic castration-resistant prostate cancer and neuroendocrine differentiation. *Clin Nucl Med* 2019;**44**(12):978-80.

30. Ataeinia B, Loberg C, Kravets H, et al. Successful palliative peptide receptor radionuclide therapy for impending compression of vena cava due to unresectable liver metastasis of neuroendocrine tumor. *EXCLI J* 2019;**18**:273-6.

31. Auernhammer CJ, Göke B. Therapeutic strategies for advanced neuroendocrine carcinomas of jejunum/ileum and pancreatic origin. *Gut* 2011;**60**(7):1009-21.

32. Auernhammer CJ, Spitzweg C, Angele MK, et al. Advanced neuroendocrine tumours of the small intestine and pancreas: clinical developments, controversies, and future strategies. *Lancet Diabetes Endocrinol* 2018;**6**(5):404-15.

33. Auernhammer CJ, Spitzweg C, Heinemann V, Göke B. Medicinal therapy of metastasized neuroendocrine tumors of the gastroenteropancreatic system [article in German]. *Internist (Berl)* 2012; Feb 2. doi: 10.1007/s00108-011-2919-z.

34. Baechler S, Hobbs RF, Boubaker A, et al. Three-dimensional radiobiological dosimetry of kidneys for treatment planning in peptide receptor radionuclide therapy. *Med Phys* 2012;**39**(10):6118-28.

35. Bailey DL, Hennessy TM, Willowson KP, et al. In vivo measurement and characterization of a novel formulation of [(177)Lu]-DOTA-Octreotate. *Asia Ocean J Nucl Med Biol* 2016;**4**(1):30-7.

36. Bandara N, Jacobson O, Mpoy C, Chen X, Rogers BE. Novel structural modification based on evans blue dye to improve pharmacokinetics of a somastostatin-receptor-based theranostic agent. *Bioconjug Chem* 2018;**29**(7):2448-54.

37. Barber TW, Hofman MS, Thomson BN, Hicks RJ. The potential for induction peptide receptor chemoradionuclide therapy to render inoperable pancreatic and duodenal neuroendocrine tumours resectable. *Eur J Surg Oncol* 2012;**38**(1):64-71.

38. Barbier CE, Garske-Román U, Sandström M, Nyman R, Granberg D. Selective internal radiation therapy in patients with progressive neuroendocrine liver metastases. *Eur J Nucl Med Mol Imaging* 2016;**43**(8):1425-31.

39. Barriuso J, Custodio A, Afonso R, et al. Prognostic and predictive biomarkers for somatostatin analogs, peptide receptor radionuclide therapy and serotonin pathway targets in neuroendocrine tumours. *Cancer Treat Rev* 2018;**70**:209-22.

40. Basu S. Should grade of tracer uptake on somatostatin receptor-targeted imaging be the major determinant and break the barrier of histopathologic criteria for determining the suitability of Peptide receptor radionuclide therapy? *J Nucl Med* 2013;**54**(11):2018-9.

41. Basu S, Abhyankar A. The use of 99mTc-HYNIC-TOC and 18F-FDG PET/CT in the evaluation of duodenal neuroendocrine tumor with atypical and extensive metastasis responding dramatically to a single fraction of PRRT with 177Lu-DOTATATE. *J Nucl Med Technol* 2014;**42**(4):296-8.

42. Basu S, Chakraborty S, Parghane RV, et al. One decade of 'bench-to-bedside' peptide receptor radionuclide therapy with indigenous [(177)Lu]Lu-DOTATATE obtained through 'direct' neutron activation route: lessons learnt including practice evolution in an Indian setting. *Am J Nucl Med Mol Imaging* 2020;**10**(4):178-211.

43. Basu S, Fargose P. 177Lu-DOTATATE PRRT in recurrent skull-base phosphaturic mesenchymal tumor causing osteomalacia: a potential application of PRRT beyond neuroendocrine tumors. *J Nucl Med Technol* 2016;**44**(4):248-50.

44. Basu S, Ostwal V. The case for combined chemotherapy-peptide receptor radionuclide therapy (chemo-PRRT) strategy in metastatic neuroendocrine tumor: predicting and looking at the possible case scenarios. *Eur J Nucl Med Mol Imaging* 2016;**43**(13):2453-5.

45. Basu S, Ostwal V, Ranade R, Panda D. Supportive measures and finer practice points in 177Lu-DOTATATE PRRT for NET: aiming for optimal disease management. *J Nucl Med* 2014;**55**(11):1916-7.

46. Basu S, Parghane RV, Ostwal V, Shrikhande SV. Neoadjuvant strategies for advanced pancreatic neuroendocrine tumors: should combined chemotherapy and peptide receptor radionuclide therapy be the preferred regimen for maximizing outcome? *Nucl Med Commun* 2018;**39**(1):94-5.

47. Basu S, Ranade R. Favorable response of metastatic merkel cell carcinoma to targeted 177Lu-DOTATATE therapy: will PRRT evolve to become an important approach in receptor-positive cases? *J Nucl Med Technol* 2016;**44**(2):85-7.

48. Beaino W, Nedrow JR, Anderson CJ. Evaluation of (68)Ga- and (177)Lu-DOTA-PEG4-LLP2A for VLA-4-targeted PET imaging and treatment of metastatic melanoma. *Mol Pharm* 2015;**12**(6):1929-38.

49. Beauregard JM, Hofman MS, Kong G, Hicks RJ. The tumour sink effect on the biodistribution of 68Ga-DOTA-octreotate: implications for peptide receptor radionuclide therapy. *Eur J Nucl Med Mol Imaging* 2012;**39**(1):50-6.

50. Béhé M, Kluge G, Becker W, Gotthardt M, Behr TM. Use of polyglutamic acids to reduce uptake of radiometal-labeled minigastrin in the kidneys. *J Nucl Med* 2005;**46**(6):1012-5.

51. Behnammanesh H, Erfani M, Hajiramezanali M, et al. Preclinical study of a new (177)Lu-labeled somatostatin receptor antagonist in HT-29 human colorectal cancer cells. *Asia Ocean J Nucl Med Biol* 2020;**8**(2):109-15.

52. Behnammanesh H, Jokar S, Erfani M, et al. Design, preparation and biological evaluation of a (177)Lu-labeled somatostatin receptor antagonist for targeted therapy of neuroendocrine tumors. *Bioorg Chem* 2020;**94**:103381.

53. Berardi R, Rinaldi S, Torniai M, et al. Gastrointestinal neuroendocrine tumors: searching the optimal treatment strategy – A literature review. *Crit Rev Oncol Hematol* 2016;**98**:264-74.

54. Bergsma H, van Lom K, Raaijmakers M, et al. Persistent hematologic dysfunction after peptide receptor radionuclide therapy with (177)Lu-DOTATATE: incidence, course, and predicting factors in patients with gastroentero-pancreatic neuroendocrine tumors. *J Nucl Med* 2018;**59**(3):452-8.

55. Bergsma H, van Vliet EI, Teunissen JJ, et al. Peptide receptor radionuclide therapy (PRRT) for GEP-NETs. *Best Pract Res Clin Gastroenterol* 2012;**26**(6):867-81.

56. Bernhardt P, Oddstig J, Kölby L, Nilsson O, Ahlman H, Forssell-Aronsson E. Effects of treatment with (177)Lu-DOTA-Tyr(3)-octreotate on uptake of subsequent injection in carcinoid-bearing nude mice. *Cancer Biother Radiopharm* 2007;**22**(5):644-53.

57. Bertani E, Fazio N, Radice D, et al. Resection of the primary tumor followed by peptide receptor radionuclide therapy as upfront strategy for the treatment of G1-G2 pancreatic neuroendocrine tumors with unresectable liver metastases. *Ann Surg Oncol* 2016;**23**(Suppl 5):981-9.

58. Beykan S, Dam JS, Eberlein U, et al. (177)Lu-OPS201 targeting somatostatin receptors: in vivo biodistribution and dosimetry in a pig model. *EJNMMI Res* 2016;**6**(1):50.

59. Beykan S, Fani M, Jensen SB, et al. In vivo biokinetics of (177)Lu-OPS201 in mice and pigs as a model for predicting human dosimetry. *Contrast Media Mol Imaging* 2019;**2019**:6438196.

60. Binderup T, Knigge U, Johnbeck CB, et al. (18)F-FDG-PET is superior to WHO grading as prognostic tool in neuroendocrine neoplasms and useful in guiding peptide receptor radionuclide therapy: a prospective 10-year follow-up study of 166 patients. *J Nucl Med* 2021;**62**(6):808-15.

61. Blaickner M, Baum RP. Relevance of PET for pretherapeutic prediction of doses in peptide receptor radionuclide therapy. *PET Clin* 2014;**9**(1):99-112.

62. Blažević A, Brabander T, Zandee WT, et al. Evolution of the mesenteric mass in small intestinal neuroendocrine tumours. *Cancers (Basel)* 2021;**13**(3):443.

63. Bober B, Saracyn M, Kołodziej M, et al. Carcinoid heart disease: how to diagnose and treat in 2020? *Clin Med Insights Cardiol* 2020;**14**:1179546820968101.

64. Bober B, Zaleska M, Kołodziej M, Kowalski Ł, Saracyn M. Treatment of neuroendocrine tumors with radio-labeled somatostatin analogues [article in Polish]. *Wiad Lek* 2018;**71**(9):1770-3.

65. Bodei L, Cremonesi M, Grana CM, et al. Yttrium-labelled peptides for therapy of NET. *Eur J Nucl Med Mol Imaging* 2012;**39**(Suppl 1): S93-102.

66. Bodei L, Cremonesi M, Paganelli G. Yttrium-based therapy for neuroendocrine tumors. *PET Clin* 2014;**9**(1):71-82.

67. Bodei L, Kwekkeboom DJ, Kidd M, Modlin IM, Krenning EP. Radiolabeled somatostatin analogue therapy of gastroenteropancreatic cancer. *Semin Nucl Med* 2016;**46**(3):225-38.

68. Bodelier AG, Haak HR. Gastroenteropancreatic neuroendocrine tumours (carcinoid tumours): definition, clinical aspects, diagnosis and therapy [article in Dutch]. *Ned Tijdschr Geneeskd* 2006;**150**(34):1868-72.

69. Bolzati C, Duatti A. The emerging value of 64Cu for molecular imaging and therapy. *Q J Nucl Med Mol Imaging* 2020;**64**(4):329-37.

70. Bordeianu C, Parat A, Piant S, et al. Evaluation of the active targeting of melanin granules after intravenous injection of dendronized nanoparticles. *Mol Pharm* 2018;**15**(2):536-47.

71. Borgna F, Barritt P, Grundler PV, et al. Simultaneous visualization of (161)Tb- and (177)Lu-labeled somatostatin analogues using dual-isotope SPECT imaging. *Pharmaceutics* 2021;**13**(4):536.

72. Boucher JE, Sommers R. Targeted therapy: new radiolabeled somatostatin analogs to treat gastroenteropancreatic neuroendocrine tumors. *Clin J Oncol Nurs* 2018;**22**(5):565-8.

73. Braat A, Bruijnen RCG, van Rooij R, et al. Additional holmium-166 radioembolisation after lutetium-177-dotatate in patients with neuroendocrine tumour liver metastases (HEPAR PLuS): a single-centre, single-arm, open-label, phase 2 study. *Lancet Oncol* 2020;**21**(4):561-70.

74. Braat A, Kwekkeboom DJ, Kam BLR, et al. Additional hepatic (166)Ho-radioembolization in patients with neuroendocrine tumours treated with (177)Lu-DOTATATE; a single center, interventional, non-randomized, non-comparative, open label, phase II study (HEPAR PLUS trial). *BMC Gastroenterol* 2018;**18**(1):84.

75. Braat A, Snijders TJ, Seute T, Vonken EPA. Will (177)Lu-DOTATATE treatment become more effective in salvage meningioma patients, when boosting somatostatin receptor saturation? a promising case on intra-arterial administration. *Cardiovasc Intervent Radiol* 2019;**42**(11):1649-52.

76. Brabander T, Kwekkeboom DJ, Feelders RA, Brouwers AH, Teunissen JJ. Nuclear medicine imaging of neuroendocrine tumors. *Front Horm Res* 2015;**44**:73-87.

77. Brabander T, van der Zwan WA, Teunissen JJM, et al. Pitfalls in the response evaluation after peptide receptor radionuclide therapy with [(177)Lu-DOTA(0),Tyr(3)]octreotate. *Endocr Relat Cancer* 2017;**24**(5):243-51.

78. Breeman WA, Chan HS, de Zanger RM, Konijnenberg MK, de Blois E. Overview of development and formulation of ^{177}Lu-DOTA-TATE for PRRT. *Curr Radiopharm* 2016;**9**(1):8-18.

79. Briest F, Koziolek EJ, Albrecht J, et al. Does the proteasome inhibitor bortezomib sensitize to DNA-damaging therapy in gastroenteropancreatic neuroendocrine neoplasms? - A preclinical assessment in vitro and in vivo. *Neoplasia* 2021;**23**(1):80-98.

80. Briganti V, Cuccurullo V, Berti V, et al. (99m)Tc-EDDA/HYNIC-TOC is a new opportunity in neuroendocrine tumors of the lung (and in other malignant and benign pulmonary diseases). *Curr Radiopharm* 2020;**13**(3):166-76.

81. Briganti V, Cuccurullo V, Di Stasio GD, Mansi L. Gamma emitters in pancreatic endocrine tumors imaging in the PET era: is there a clinical space for 99mTc-peptides? *Curr Radiopharm* 2019;**12**(2):156-70.

82. Brown E, Watkin D, Evans J, Yip V, Cuthbertson DJ. Multidisciplinary management of refractory insulinomas. *Clin Endocrinol (Oxf)* 2018;**88**(5):615-24.

83. Burki TK. 177Lu-Dotatate for midgut neuroendocrine tumours. *Lancet Oncol* 2017;**18**(2):e74.

84. Bushnell DL, Madsen MT, O'Cdorisio T, et al. Feasibility and advantage of adding (131)I-MIBG to (90)Y-DOTATOC for treatment of patients with advanced stage neuroendocrine tumors. *EJNMMI Res* 2014;**4**(1):38.

85. Buzzoni R, Carnaghi C, Strosberg J, et al. Impact of prior therapies on everolimus activity: an exploratory analysis of RADIANT-4. *Onco Targets Ther* 2017;**10**:5013-30.

86. Calais PJ, Turner JH. Radiation safety of outpatient 177Lu-octreotate radiopeptide therapy of neuroendocrine tumors. *Ann Nucl Med* 2014;**28**(6):531-9.

87. Camacho X, Calzada V, Fernandez M, et al. 177Lu-DOTA-bevacizumab: radioimmunotherapy agent for melanoma. *Curr Radiopharm* 2017;**10**(1):21-8.

88. Campana D, Capurso G, Partelli S, et al. Radiolabelled somatostatin analogue treatment in gastroenteropancreatic neuroendocrine tumours: factors associated with response and suggestions for therapeutic sequence. *Eur J Nucl Med Mol Imaging* 2013;**40**(8):1197-205.

89. Carlsen EA, Fazio N, Granberg D, et al. Peptide receptor radionuclide therapy in gastroenteropancreatic NEN G3: a multicenter cohort study. *Endocr Relat Cancer* 2019;**26**(2):227-39.

90. Castellano D, Grande E, Valle J, et al. Expert consensus for the management of advanced or metastatic pancreatic neuroendocrine and carcinoid tumors. *Cancer Chemother Pharmacol* 2015;**75**(6):1099-114.

91. Cescato R, Waser B, Fani M, Reubi JC. Evaluation of 177Lu-DOTA-sst2 antagonist versus 177Lu-DOTA-sst2 agonist binding in human cancers in vitro. *J Nucl Med* 2011;**52**(12):1886-90.

92. Chakraborty S, Sarma HD, Vimalnath KV, Pillai MR. Tracer level radiochemistry to clinical dose preparation of (177)Lu-labeled cyclic RGD peptide dimer. *Nucl Med Biol* 2013;**40**(7):946-54.

93. Chalkia MT, Stefanoyiannis AP, Chatziioannou SN, Round WH, Efstathopoulos EP, Nikiforidis GC. Patient-specific dosimetry in peptide receptor radionuclide therapy: a clinical review. *Australas Phys Eng Sci Med* 2015;**38**(1):7-22.

94. Chan HS, Konijnenberg MW, Daniels T, et al. Improved safety and efficacy of (213)Bi-DOTATATE-targeted alpha therapy of somatostatin receptor-expressing neuroendocrine tumors in mice pre-treated with L-lysine. *EJNMMI Res* 2016;**6**(1):83.

95. Chan TG, O'Neill E, Habjan C, Cornelissen B. Combination strategies to improve targeted radionuclide therapy. *J Nucl Med* 2020;**61**(11):1544-52.

96. Chaushev B, Bochev P, Klisarova A, et al. Cervix carcinoma and incidental finding of medullary thyroid carcinoma by 18F-FDG PET/CT – clinical case. *Nucl Med Rev Cent East Eur* 2014;**17**(2):97-100.

97. Chen L, Chen J, Zhou Z. Interpretation of the latest guidelines in the treatment of gastrointestinal neuroendocrine neoplasms [article in Chinese]. *Zhonghua Wei Chang Wai Ke Za Zhi* 2016;**19**(11):1201-4.

98. Chevalier E, Boursier C, Claudin M, Marie PY, Imbert L. Feasibility of 177Lu therapy monitoring using fast whole-body SPECT recordings provided by a high-speed 360° CZT camera. *Clin Nucl Med* 2020;**45**(11):e493-e4.

99. Chicheportiche A, Artoul F, Schwartz A, et al. Reducing the number of CTs performed to monitor personalized dosimetry during peptide receptor radionuclide therapy (PRRT). *EJNMMI Phys* 2018;**5**(1):10.

100. Chicheportiche A, Ben-Haim S, Grozinsky-Glasberg S, et al. Dosimetry after peptide receptor radionuclide therapy: impact of reduced number of post-treatment studies on absorbed dose calculation and on patient management. *EJNMMI Phys* 2020;**7**(1):5.

101. Cho CM. Recent updates in the management of advanced pancreatic neuroendocrine tumors. *Korean J Gastroenterol* 2019;**73**(3):124-31.

102. Choi J, Beaino W, Fecek RJ, et al. Combined VLA-4-targeted radionuclide therapy and immunotherapy in a mouse model of melanoma. *J Nucl Med* 2018;**59**(12):1843-9.

103. Chougnet CN, Leboulleux S, Caramella C, et al. Frequency and characterization of gastro-entero-pancreatic neuroendocrine tumor patients with high-grade of uptake at somatostatin receptor scintigraphy. *Endocr Relat Cancer* 2013;**20**(2):229-39.

104. Cidon EU. New therapeutic approaches to metastatic gastroenteropancreatic neuroendocrine tumors: a glimpse into the future. *World J Gastrointest Oncol* 2017;**9**(1):4-20.

105. Cieciera M, Kratochwil C, Moltz J, et al. Semi-automatic 3D-volumetry of liver metastases from neuroendocrine tumors to improve combination therapy with 177Lu-DOTATOC and 90Y-DOTATOC. *Diagn Interv Radiol* 2016;**22**(3):201-6.

106. Cives M, Pelle E, Strosberg J. Emerging treatment options for gastroenteropancreatic neuroendocrine tumors. *J Clin Med* 2020;**9**(11):3655.

107. Cives M, Strosberg JR. Gastroenteropancreatic neuroendocrine tumors. *CA Cancer J Clin* 2018;**68**(6):471-87.

108. Claringbold PG, Price RA, Turner JH. Phase I-II study of radiopeptide 177Lu-octreotate in combination with capecitabine and temozolomide in advanced low-grade neuroendocrine tumors. *Cancer Biother Radiopharm* 2012;**27**(9):561-9.

109. Claringbold PG, Turner JH. Neuroendocrine tumor therapy with lutetium-177-octreotate and everolimus (NET-TLE): a phase I study. *Cancer Biother Radiopharm* 2015;**30**(6):261-9.

110. Cloyd JM, Konda B, Shah MH, Pawlik TM. The emerging role of targeted therapies for advanced well-differentiated gastroenteropancreatic neuroendocrine tumors. *Expert Rev Clin Pharmacol* 2019;**12**(2):101-8.

111. Costa R, Costa R, Bacchi CE, Almeida Filho P. Metastatic insulinoma managed with radiolabeled somatostatin analog. *Case Rep Endocrinol* 2013;**2013**:252159.

112. Cuccurullo V, Di Stasio GD, Mansi L. Physiopathological premises to nuclear medicine imaging of pancreatic neuroendocrine tumours. *Curr Radiopharm* 2019;**12**(2):98-106.

113. Cullinane C, Jeffery CM, Roselt PD, et al. Peptide receptor radionuclide therapy with (67)Cu-CuSarTATE is highly efficacious against a somatostatin-positive neuroendocrine tumor model. *J Nucl Med* 2020;**61**(12):1800-5.

114. Cullinane C, Waldeck K, Kirby L, et al. Enhancing the anti-tumour activity of (177)Lu-DOTA-octreotate radionuclide therapy in somatostatin receptor-2 expressing tumour models by targeting PARP. *Sci Rep* 2020;**10**(1):10196.

115. da Silva TN, van Velthuysen MLF, van Eijck CHJ, Teunissen JJ, Hofland J, de Herder WW. Successful neoadjuvant peptide receptor radionuclide therapy for an inoperable pancreatic neuroendocrine tumour. *Endocrinol Diabetes Metab Case Rep* 2018;**2018**:18-0015.

116. Dalm SU, Martens JW, Sieuwerts AM, et al. In vitro and in vivo application of radiolabeled gastrin-releasing peptide receptor ligands in breast cancer. *J Nucl Med* 2015;**56**(5):752-7.

117. Dalm SU, Nonnekens J, Doeswijk GN, et al. Comparison of the therapeutic response to treatment with a 177Lu-labeled somatostatin receptor agonist and antagonist in preclinical models. *J Nucl Med* 2016;**57**(2):260-5.

118. Dalmo J, Spetz J, Montelius M, et al. Priming increases the anti-tumor effect and therapeutic window of (177)Lu-octreotate in nude mice bearing human small intestine neuroendocrine tumor GOT1. *EJNMMI Res* 2017;**7**(1):6.

119. Das S, Al-Toubah T, El-Haddad G, Strosberg J. (177)Lu-DOTATATE for the treatment of gastroenteropancreatic neuroendocrine tumors. *Expert Rev Gastroenterol Hepatol* 2019;**13**(11):1023-31.

120. Das S, Pineda G, Goff L, Sobel R, Berlin J, Fisher G. The eye of the beholder: orbital metastases from midgut neuroendocrine tumors, a two institution experience. *Cancer Imaging* 2018;**18**(1):47.

121. Das T, Banerjee S, Shinto A, Kamaleshwaran KK, Sarma HD. Preparation of therapeutic dose of 177Lu-DOTA-TATE using a novel single vial freeze-dried kit: a comparison with 'in-situ' preparation at hospital radiopharmacy. *Curr Radiopharm* 2014;**7**(1):12-9.

122. Das T, Chakraborty S, Kallur KG, Venkatesh M, Banerjee S. Preparation of patient doses of (177)Lu-DOTA-TATE using indigenously produced (177)Lu: the Indian experience. *Cancer Biother Radiopharm* 2011;**26**(3):395-400.

123. Davis AB, Pietryka MH, Passalaqua S. Technical aspects and administration methods of (177)Lu-DOTATATE for nuclear medicine technologists. *J Nucl Med Technol* 2019;**47**(4):288-91.

124. de Araújo EB, Caldeira Filho JS, Nagamati LT, et al. A comparative study of 131I and 177Lu labeled somatostatin analogues for therapy of neuroendocrine tumours. *Appl Radiat Isot* 2009;**67**(2):227-33.

125. De Araújo EB, Pujatti PB, Mengatti J. Radiolabeling of substance P with lutetium-177 and biodistribution study in rat pancreatic tumor xenografted nude mice. *Cell Mol Biol (Noisy-le-grand)* 2010;**56**(2):12-7.

126. De Both A, De Man M, Troisi R, Van Vlierberghe H, Hoorens A, Geboes K. Treatment of a mixed acinar-endocrine carcinoma with uptake on (68)gallium-DOTATOC positron emission tomography-computed tomography: a case report. *Oncol Lett* 2017;**14**(1):547-52.

127. De Jong M, Breeman WA, Bernard HF, et al. Therapy of neuroendocrine tumors with radiolabeled somatostatin-analogues. *Q J Nucl Med* 1999;**43**(4):356-66.

128. de Mestier L, Deguelte-Lardière S, Brixi H, Kianmanesh R, Cadiot G.Digestive neuroendocrine tumors [article in French]. *Rev Med Interne* 2016;**37**(8):551-60.

129. de Mestier L, Lepage C, Baudin E, et al. Digestive neuroendocrine neoplasms (NEN): French Intergroup clinical practice guidelines for diagnosis, treatment and follow-up (SNFGE, GTE, RENATEN, TENPATH, FFCD, GERCOR, UNICANCER, SFCD, SFED, SFRO, SFR). *Dig Liver Dis* 2020;**52**(5):473-92.

130. Del Prete M, Arsenault F, Saighi N, et al. Accuracy and reproducibility of simplified QSPECT dosimetry for personalized (177)Lu-octreotate PRRT. *EJNMMI Phys* 2018;**5**(1):25.

131. Del Prete M, Buteau FA, Beauregard JM. Personalized (177)Lu-octreotate peptide receptor radionuclide therapy of neuroendocrine tumours: a simulation study. *Eur J Nucl Med Mol Imaging* 2017;**44**(9):1490-500.

132. Delker A, Ilhan H, Zach C, et al. The influence of early measurements onto the estimated kidney dose in [(177)Lu][DOTA(0),Tyr(3)]octreotate peptide receptor radiotherapy of neuroendocrine tumors. *Mol Imaging Biol* 2015;**17**(5):726-34.

133. Delpassand ES, Samarghandi A, Mourtada JS, et al. Long-term survival, toxicity profile, and role of F-18 FDG PET/CT scan in patients with progressive neuroendocrine tumors following peptide receptor radionuclide therapy with high activity in-111 pentetreotide. *Theranostics* 2012;**2**(5):472-80.

134. Delpassand ES, Samarghandi A, Zamanian S, et al. Peptide receptor radionuclide therapy with 177Lu-DOTATATE for patients with somatostatin receptor-expressing neuroendocrine tumors: the first US phase 2 experience. *Pancreas* 2014;**43**(4):518-25.

135. Derlin T. Neuroendocrine tumor therapy (NETTER-1) trial: (177)Lu-DOTA-TATE for neuroendocrine tumors [article in German]. *Radiologe* 2017;**57**(5):343-5.

136. Deroose CM, Hindié E, Kebebew E, et al. Molecular imaging of gastroenteropancreatic neuroendocrine tumors: current status and future directions. *J Nucl Med* 2016;**57**(12):1949-56.

137. Desai GS, Pande P, Chhabra V, Shah RC, Jagannath P. Multimodality management, recurrence patterns, and long-term outcome of gastroenteropancreatic neuroendocrine neoplasms: progress over 17 years. *Indian J Gastroenterol* 2019;**38**(5):399-410.

138. Desai KK, Khan MS, Toumpanakis C, Caplin ME. Management of gastroentero-pancreatic neuroendocrine tumors (GEP-NETs). *Minerva Gastroenterol Dietol* 2009;**55**(4):425-43.

139. Diakatou E, Alexandraki KI, Tsolakis AV, et al. Somatostatin and dopamine receptor expression in neuroendocrine neoplasms: correlation of immunohistochemical findings with somatostatin receptor scintigraphy visual scores. *Clin Endocrinol (Oxf)* 2015;**83**(3):420-8.

140. Dierickx LO, Séverine B, Fatima M, Amel B, Rosine G. Successful and safe treatment with 177Lu-DOTATATE (Lutathera) of progressive metastatic pancreatic neuroendocrine tumor under hemodialysis. *Clin Nucl Med* 2020;**45**(9):e400-e2.

141. Dieudonné A, Hobbs RF, Lebtahi R, et al. Study of the impact of tissue density heterogeneities on 3-dimensional abdominal dosimetry: comparison between dose kernel convolution and direct Monte Carlo methods. *J Nucl Med* 2013;**54**(2):236-43.

142. Dobson R, Vinjamuri S, Hsuan J, et al. Treatment of orbital metastases from a primary midgut neuroendocrine tumor with peptide-receptor radiolabeled therapy using 177 lutetium-DOTATATE. *J Clin Oncol* 2013;**31**(17):e272-5.

143. Dumlu EG, Karakoç D, Özdemir A. Nonfunctional pancreatic neuroendocrine tumors: advances in diagnosis, management, and controversies. *Int Surg* 2015;**100**(6):1089-97.

144. Elf AK, Bernhardt P, Hofving T, et al. NAMPT inhibitor GMX1778 enhances the efficacy of 177Lu-DOTATATE treatment of neuroendocrine tumors. *J Nucl Med* 2017;**58**(2):288-92.

145. Elf AK, Johanson V, Marin I, et al. Evaluation of SSTR2 expression in SI-NETs and relation to overall survival after PRRT. *Cancers (Basel)* 2021;**13**(9):2035.

146. Ergül N, Çermik TF, Dursun N. Is it possible NET-redifferentiation after chemotherapy? (68)Ga DOTA-peptide imaging as a game-changer in therapy management. *Rev Esp Med Nucl Imagen Mol* 2020;**39**(1):31-4.

147. Exner S, Prasad V, Wiedenmann B, Grötzinger C. Octreotide does not inhibit proliferation in five neuroendocrine tumor cell lines. *Front Endocrinol (Lausanne)* 2018;**9**:146.

148. Ezziddin S, Attassi M, Yong-Hing CJ, et al. Predictors of long-term outcome in patients with well-differentiated gastroenteropancreatic neuroendocrine tumors after peptide receptor radionuclide therapy with 177Lu-octreotate. *J Nucl Med* 2014;**55**(2):183-90.

149. Ezziddin S, Khalaf F, Vanezi M, et al. Outcome of peptide receptor radionuclide therapy with 177Lu-octreotate in advanced grade 1/2 pancreatic neuroendocrine tumours. *Eur J Nucl Med Mol Imaging* 2014;**41**(5):925-33.

150. Ezziddin S, Lauschke H, Schaefers M, et al. Neoadjuvant downsizing by internal radiation: a case for preoperative peptide receptor radionuclide therapy in patients with pancreatic neuroendocrine tumors. *Clin Nucl Med* 2012;**37**(1):102-4.

151. Ezziddin S, Meyer C, Kahancova S, et al. 90Y Radioembolization after radiation exposure from peptide receptor radionuclide therapy. *J Nucl Med* 2012;**53**(11):1663-9.

152. Ezziddin S, Opitz M, Attassi M, et al. Impact of the Ki-67 proliferation index on response to peptide receptor radionuclide therapy. *Eur J Nucl Med Mol Imaging* 2011;**38**(3):459-66.

153. Ezziddin S, Reichmann K, Yong-Hing C, et al. Early prediction of tumour response to PRRT. The sequential change of tumour-absorbed doses during treatment with 177Lu-octreotate. *Nuklearmedizin* 2013;**52**(5):170-7.

154. Ezziddin S, Sabet A, Heinemann F, et al. Response and long-term control of bone metastases after peptide receptor radionuclide therapy with (177)Lu-octreotate. *J Nucl Med* 2011;**52**(8):1197-203.

155. Fabbri C, Bartolomei M, Mattone V, et al. (90)Y-PET/CT imaging quantification for dosimetry in peptide receptor radionuclide therapy: analysis and corrections of the impairing factors. *Cancer Biother Radiopharm* 2015;**30**(5):200-10.

156. Falconi M, Fazio N, Ferone D, Versari A. Use of octreotide long acting repeatable (LAR) as second-line therapy in advanced neuroendocrine tumors in different clinical settings: an Italian Delphi survey. *Expert Opin Pharmacother* 2020;**21**(18):2317-24.

157. Falkmer UG, Gustafsson T, Wenzel R, et al. Malignant presacral ghrelinoma with long-standing hyperghrelinaemia. *Ups J Med Sci* 2015;**120**(4):299-304.

158. Fani M, Mueller A, Tamma ML, et al. Radiolabeled bicyclic somatostatin-based analogs: a novel class of potential radiotracers for SPECT/PET of neuroendocrine tumors. *J Nucl Med* 2010;**51**(11):1771-9.

159. Fani M, Nicolas GP, Wild D. Somatostatin receptor antagonists for imaging and therapy. *J Nucl Med* 2017;**58**(Suppl 2):61s-6s.

160. Farooqui ZA, Chauhan A. Neuroendocrine tumors in pediatrics. *Glob Pediatr Health* 2019;**6**:2333794x19862712.

161. Fazio N, Cella CA, Del Re M, et al. Pharmacodynamics, clinical findings and approval status of current and emerging tyrosine-kinase inhibitors for pancreatic neuroendocrine tumors. *Expert Opin Drug Metab Toxicol* 2019;**15**(12):993-1004.

162. Fazio N, Cinieri S, Lorizzo K, et al. Biological targeted therapies in patients with advanced enteropancreatic neuroendocrine carcinomas. *Cancer Treat Rev* 2010;**36**(Suppl 3):S87-94.

163. Fazio N, Milione M. Heterogeneity of grade 3 gastroenteropancreatic neuroendocrine carcinomas: New insights and treatment implications. *Cancer Treat Rev* 2016;**50**:61-7.

164. Feijtel D, Doeswijk GN, Verkaik NS, et al. Inter and intra-tumor somatostatin receptor 2 heterogeneity influences peptide receptor radionuclide therapy response. *Theranostics* 2021;**11**(2):491-505.

165. Fendler WP, Barrio M, Spick C, et al. 68Ga-DOTATATE PET/CT interobserver agreement for neuroendocrine tumor assessment: results of a prospective study on 50 patients. *J Nucl Med* 2017;**58**(2):307-11.

166. Fidelman N. Holmium-166 radioembolisation after peptide receptor radionuclide therapy: much needed data to help inform future research. *Lancet Oncol* 2020;**21**(4):478-9.

167. Filippi L, Cianni R, Schillaci O, Bagni O. Molecular and metabolic imaging of hepatic neuroendocrine tumors following radioembolization with 90Y-microspheres. *Curr Med Imaging* 2020;**16**(5):545-52.

168. Filippi L, Ciorra A, Sardella B, Schillaci O, Bagni O. Sequential use of (90)Y microspheres radioembolization and (177)Lu-Dotatate in pluri-metastatic neuroendocrine tumors: a case report. *Nucl Med Mol Imaging* 2014;**48**(4):321-5.

169. Fischbach J, Gut P, Matysiak-Grześ M, et al. Combined octreotide and peptide receptor radionuclide therapy ((90)Y-DOTA-TATE) in case of malignant insulinoma. *Neuro Endocrinol Lett* 2012;**33**(3):273-8.

170. Fitschen J, Knoop BO, Behrendt R, Knapp WH, Geworski L. External radiation exposure and effective half-life in Lu-177-Dota-Tate therapy [article in German]. *Z Med Phys* 2011;**21**(4):266-73.

171. Forrer F, Krenning EP, Kooij PP, et al. Bone marrow dosimetry in peptide receptor radionuclide therapy with [177Lu-DOTA(0),Tyr(3)]octreotate. *Eur J Nucl Med Mol Imaging* 2009;**36**(7):1138-46.

172. Foster JH, Sher A, Seghers V, et al. Peptide receptor radionuclide therapy for treatment of metastatic neuroendocrine tumors in children. *Pediatr Blood Cancer* 2021;**68**(7):e29056.

173. Freedman N, Sandström M, Kuten J, et al. Personalized radiation dosimetry for PRRT-how many scans are really required? *EJNMMI Phys* 2020;**7**(1):26.

174. Frilling A, Clift AK. Combining radiolabelled therapies for neuroendocrine neoplasms. *Nat Rev Endocrinol* 2020;**16**(7):347-8.

175. Frilling A, Giele H, Vrakas G, et al. Modified liver-free multivisceral transplantation for a metastatic small bowel neuroendocrine tumor: a case report. *Transplant Proc* 2015;**47**(3):858-62.

176. Frilling A, Weber F, Saner F, et al. Treatment with (90)Y- and (177)Lu-DOTATOC in patients with metastatic neuroendocrine tumors. *Surgery* 2006;**140**(6):968-76.

177. Fröss-Baron K, Garske-Roman U, Welin S, et al. 177Lu-DOTATATE therapy of advanced pancreatic neuroendocrine tumors heavily pretreated with chemotherapy: analysis of outcome, safety, and their determinants. *Neuroendocrinology* 2021;**111**(4):330-43.

178. Gabriel M, Oberauer A, Dobrozemsky G, et al. 68Ga-DOTA-Tyr3-octreotide PET for assessing response to somatostatin-receptor-mediated radionuclide therapy. *J Nucl Med* 2009;**50**(9):1427-34.

179. Gajate P, Alonso-Gordoa T, Martínez-Sáez O, Molina-Cerrillo J, Grande E. Prognostic and predictive role of the PI3K-AKT-mTOR pathway in neuroendocrine neoplasms. *Clin Transl Oncol* 2018;**20**(5):561-9.

180. Garcia-Carbonero R, P JI-F, Teulé A, Barriuso J, Sevilla I. SEOM clinical guidelines for the diagnosis and treatment of gastroenteropancreatic neuroendocrine neoplasms (GEP-NENs) 2014. *Clin Transl Oncol* 2014;**16**(12):1025-34.

181. García-Carbonero R, Salazar R, Sevilla I, Isla D. SEOM clinical guidelines for the diagnosis and treatment of gastroenteropancreatic neuroendocrine tumours (GEP NETS). *Clin Transl Oncol* 2011;**13**(8):545-51.

182. Giesel FL, Stefanova M, Schwartz LH, et al. Impact of peptide receptor radionuclide therapy on the 68Ga-DOTATOC-PET/CT uptake in normal tissue. *Q J Nucl Med Mol Imaging* 2013;**57**(2):171-6.

183. Giovacchini G, Giovannini E, Riondato M, Ciarmiello A. Radiopharmaceuticals for the diagnosis and therapy of neuroendocrine differentiated prostate cancer. *Curr Radiopharm* 2017;**10**(1):6-15.

184. Goetz TI, Lang EW, Prante O, et al. Three-dimensional Monte Carlo-based voxel-wise tumor dosimetry in patients with neuroendocrine tumors who underwent (177)Lu-DOTATOC therapy. *Ann Nucl Med* 2020;**34**(4):244-53.

185. Goffredo V, Paradiso A, Ranieri G, Gadaleta CD. Yttrium-90 (90Y) in the principal radionuclide therapies: an efficacy correlation between peptide receptor radionuclide therapy, radioimmunotherapy and transarterial radioembolization therapy. Ten years of experience (1999-2009). *Crit Rev Oncol Hematol* 2011;**80**(3):393-410.

186. Goglia U, Ferone D, Sidoti M, et al. Treatment of a pituitary metastasis from a neuroendocrine tumour: case report and literature review. *Pituitary* 2008;**11**(1):93-102.

187. Gosewisch A, Delker A, Tattenberg S, et al. Patient-specific image-based bone marrow dosimetry in Lu-177-[DOTA(0),Tyr(3)]-octreotate and Lu-177-DKFZ-PSMA-617 therapy: investigation of a new hybrid image approach. *EJNMMI Res* 2018;**8**(1):76.

188. Gradiz R, Silva HC, Carvalho L, Botelho MF, Mota-Pinto A. MIA PaCa-2 and PANC-1 - pancreas ductal adenocarcinoma cell lines with neuroendocrine differentiation and somatostatin receptors. *Sci Rep* 2016;**6**:21648.

189. Graf F, Fahrer J, Maus S, et al. DNA double strand breaks as predictor of efficacy of the alpha-particle emitter Ac-225 and the electron emitter Lu-177 for somatostatin receptor targeted radiotherapy. *PLoS One* 2014;**9**(2):e88239.

190. Grassi E, Fioroni F, Berenato S, et al. Effect of image registration on 3D absorbed dose calculations in (177)Lu-DOTATOC peptide receptor radionuclide therapy. *Phys Med* 2018;**45**:177-85.

191. Grozinsky-Glasberg S, Barak D, Fraenkel M, et al. Peptide receptor radioligand therapy is an effective treatment for the long-term stabilization of malignant gastrinomas. *Cancer* 2011;**117**(7):1377-85.

192. Grünwald F, Ezziddin S. 131I-metaiodobenzylguanidine therapy of neuroblastoma and other neuroendocrine tumors. *Semin Nucl Med* 2010;**40**(2):153-63.

193. Grzmil M, Seebacher J, Hess D, et al. Inhibition of MNK pathways enhances cancer cell response to chemotherapy with temozolomide and targeted radionuclide therapy. *Cell Signal* 2016;**28**(9):1412-21.

194. Guo H, Miao Y. Melanoma targeting property of a Lu-177-labeled lactam bridge-cyclized alpha-MSH peptide. *Bioorg Med Chem Lett* 2013;**23**(8):2319-23.

195. Haddad T, Fard-Esfahani A, Vali R. A review of pediatric neuroendocrine tumors, their detection, and treatment by radioisotopes. *Nucl Med Commun* 2021;**42**(1):21-31.

196. Haemels M, Delaunoit T, Van Laere K, Van Cutsem E, Verslype C, Deroose CM. Peptide receptor radionuclide therapy controls inappropriate calcitriol secretion in a pancreatic neuro-endocrine tumor: a case report. *BMC Gastroenterol* 2020;**20**(1):324.

197. Hagendijk ME, van der Schans S, Boersma C, Postma MJ, van der Pol S. Economic evaluation of orphan drug lutetium-octreotate vs. octreotide long-acting release for patients with an advanced midgut neuroendocrine tumour in the Netherlands. *Eur J Health Econ* 2021;**22**(6):991-9.

198. Hagmarker L, Svensson J, Rydén T, Gjertsson P, Bernhardt P. Segmentation of whole-body images into two compartments in model for bone marrow dosimetry increases the correlation with hematological response in (177)Lu-DOTATATE treatments. *Cancer Biother Radiopharm* 2017;**32**(9):335-43.

199. Halperin DM, Dasari A, Yao JC. [177Lu-DOTA0,Tyr3]-octreotate in the treatment of midgut neuroendocrine tumors. *Future Oncol* 2016;**12**(3):313-21.

200. Hanin FX, Pauwels S, Bol A, et al. Effect of interferon-alpha treatment on [68Ga-DOTA,Tyr3,Thre8]octreotide uptake in CA20948 tumors: a small-animal PET study. *J Nucl Med* 2011;**52**(4):580-5.

201. Hänscheid H, Lapa C, Buck AK, Lassmann M, Werner RA. Absorbed dose estimates from a single measurement one to three days after the administration of 177Lu-DOTATATE/-TOC. *Nuklearmedizin* 2017;**56**(6):219-24.

202. Hänscheid H, Lapa C, Buck AK, Lassmann M, Werner RA. Dose mapping after endoradiotherapy with (177)Lu-DOTATATE/DOTATOC by a single measurement after 4 days. *J Nucl Med* 2018;**59**(1):75-81.

203. Hardiansyah D, Attarwala AA, Kletting P, Mottaghy FM, Glatting G. Prediction of time-integrated activity coefficients in PRRT using simulated dynamic PET and a pharmacokinetic model. *Phys Med* 2017;**42**:298-304.

204. Hardiansyah D, Guo W, Attarwala AA, Kletting P, Mottaghy FM, Glatting G. Treatment planning in PRRT based on simulated PET data and a PBPK model. Determination of accuracy using a PET noise model. *Nuklearmedizin* 2017;**56**(1):23-30.

205. Hardiansyah D, Maass C, Attarwala AA, et al. The role of patient-based treatment planning in peptide receptor radionuclide therapy. *Eur J Nucl Med Mol Imaging* 2016;**43**(5):871-80.

206. Hasan OK, Ravi Kumar AS, Kong G, et al. Efficacy of peptide receptor radionuclide therapy for esthesioneuroblastoma. *J Nucl Med* 2020;**61**(9):1326-30.

207. Hatipoglu E, Kepicoglu H, Rusen E, Kabasakal L, Gundogdu S, Kadioglu P. Von Hippel Lindau disease with metastatic pancreatic neuroendocrine tumor causing ectopic Cushing's syndrome. *Neuro Endocrinol Lett* 2013;**34**(1):9-13.

208. Hauck L, Bitzer M, Malek N, Plentz RR. Subgroup analysis of patients with G2 gastroenteropancreatic neuroendocrine tumors. *Scand J Gastroenterol* 2016;**51**(1):55-9.

209. Hauser H, Gerson DS, Reidy-Lagunes D, Raj N. Systemic therapies for metastatic pancreatic neuroendocrine tumors. *Curr Treat Options Oncol* 2019;**20**(12):87.

210. Hendlisz A, Flamen P, Van den Eynde M, et al. Locoregional and radioisotopic targeted treatment of neuroendocrine tumours. *Acta Gastroenterol Belg* 2009;**72**(1):44-8.

211. Hicks RJ, Jackson P, Kong G, et al. (64)Cu-SARTATE PET imaging of patients with neuroendocrine tumors demonstrates high tumor uptake and retention, potentially allowing prospective dosimetry for peptide receptor radionuclide therapy. *J Nucl Med* 2019;**60**(6):777-85.

212. Hindié E. The NETPET Score: combining FDG and somatostatin receptor imaging for optimal management of patients with metastatic well-differentiated neuroendocrine tumors. *Theranostics* 2017;**7**(5):1159-63.

213. Hirmas N, Jadaan R, Al-Ibraheem A. Peptide receptor radionuclide therapy and the treatment of gastroentero-pancreatic neuroendocrine tumors: current findings and future perspectives. *Nucl Med Mol Imaging* 2018;**52**(3):190-9.

214. Hofland LJ, Capello A, Krenning EP, de Jong M, van Hagen MP. Induction of apoptosis with hybrids of Arg-Gly-Asp molecules and peptides and antimitotic effects of hybrids of cytostatic drugs and peptides. *J Nucl Med* 2005;**46**(Suppl 1):191s-8s.

215. Hofman MS, Lau WF, Hicks RJ. Somatostatin receptor imaging with 68Ga DOTATATE PET/CT: clinical utility, normal patterns, pearls, and pitfalls in interpretation. *Radiographics* 2015;**35**(2):500-16.

216. Hofman MS, Michael M, Hicks RJ. 177Lu-Dotatate for midgut neuroendocrine tumors. *N Engl J Med* 2017;**376**(14):1390-1.

217. Hofving T, Sandblom V, Arvidsson Y, et al. 177Lu-octreotate therapy for neuroendocrine tumours is enhanced by Hsp90 inhibition. *Endocr Relat Cancer* 2019;**26**(4):437-49.

218. Hope TA, Abbott A, Colucci K, et al. NANETS/SNMMI procedure standard for somatostatin receptor-based peptide receptor radionuclide therapy with (177)Lu-DOTATATE. *J Nucl Med* 2019;**60**(7):937-43.

219. Hope TA, Bodei L, Chan JA, et al. NANETS/SNMMI consensus statement on patient selection and appropriate use of (177)Lu-DOTATATE peptide receptor radionuclide therapy. *J Nucl Med* 2020;**61**(2):222-7.

220. Hope TA, Calais J, Zhang L, Dieckmann W, Millo C. (111)In-pentetreotide scintigraphy versus (68)Ga-DOTATATE PET: impact on Krenning scores and effect of tumor burden. *J Nucl Med* 2019;**60**(9):1266-9.

221. Hörsch D, Bert T, Schrader J, et al. Pancreatic neuroendocrine neoplasms. *Minerva Gastroenterol Dietol* 2012;**58**(4):401-26.

222. Hosono M, Ikebuchi H, Nakamura Y, et al. Manual on the proper use of lutetium-177-labeled somatostatin analogue (Lu-177-DOTA-TATE) injectable in radionuclide therapy (2nd ed.). *Ann Nucl Med* 2018;**32**(3):217-35.

223. Hou X, Zhao W, Beauregard JM, Celler A. Personalized kidney dosimetry in (177)Lu-octreotate treatment of neuroendocrine tumours: a comparison of kidney dosimetry estimates based on a whole organ and small volume segmentations. *Phys Med Biol* 2019;**64**(17):175004.

224. Huizing DMV, Aalbersberg EA, van der Hiel B, Stokkel MPM, Versleijen MWJ. Discordant uptake between diagnostic 68Ga-HA-DOTATATE PET/CT and posttherapy 177Lu-HA-DOTATATE SPECT/CT in patients with neuroendocrine tumors. *Clin Nucl Med* 2021;**46**(9):e475-7.

225. Ilan E, Sandström M, Wassberg C, et al. Dose response of pancreatic neuroendocrine tumors treated with peptide receptor radionuclide therapy using 177Lu-DOTATATE. *J Nucl Med* 2015;**56**(2):177-82.

226. Ilhan H, Wang H, Gildehaus FJ, et al. Nephroprotective effects of enalapril after [177Lu]-DOTATATE therapy using serial renal scintigraphies in a murine model of radiation-induced nephropathy. *EJNMMI Res* 2016;**6**(1):64.

227. Ilie MD, Lasolle H, Raverot G. Emerging and novel treatments for pituitary tumors. *J Clin Med* 2019;**8**(8):1107.

228. Iliuta IA, Beauregard JM, Couture F, Douville P, Mac-Way F. Reversal of severe and refractory humoral hypercalcemia with 177Lu-octreotate peptide receptor radionuclide therapy for neuroendocrine tumor of the pancreas. *Clin Nucl Med* 2015;**40**(9):e448-50.

229. Inno A, Bogina G, Turazza M, et al. neuroendocrine carcinoma of the breast: current evidence and future perspectives. *Oncologist* 2016;**21**(1):28-32.

230. Ito T, Jensen RT. Molecular imaging in neuroendocrine tumors: recent advances, controversies, unresolved issues, and roles in management. *Curr Opin Endocrinol Diabetes Obes* 2017;**24**(1):15-24.

231. Jackson PA, Beauregard JM, Hofman MS, Kron T, Hogg A, Hicks RJ. An automated voxelized dosimetry tool for radionuclide therapy based on serial quantitative SPECT/CT imaging. *Med Phys* 2013;**40**(11):112503.

232. Jadhav S, Basu S. Metastatic large cell neuroendocrine carcinoma of larynx: Individualizing tumor biology by dual tracer positron emission tomography/computed tomography ((68)Ga-DOTATATE and (18)F-fluorodeoxyglucose) molecular imaging and disease stabilization following (177)Lu-DOTATATE peptide receptor radionuclide therapy after initial progression on chemoradiotherapy. *World J Nucl Med* 2019;**18**(4):431-3.

233. Jahn U, Ilan E, Sandström M, Garske-Román U, Lubberink M, Sundin A. 177Lu-DOTATATE peptide receptor radionuclide therapy: dose response in small intestinal neuroendocrine tumors. *Neuroendocrinology* 2020;**110**(7-8):662-70.

234. Jahn U, Ilan E, Sandström M, Lubberink M, Garske-Román U, Sundin A. Peptide receptor radionuclide therapy (PRRT) with (177)Lu-DOTATATE; differences in tumor dosimetry, vascularity and lesion metrics in pancreatic and small intestinal neuroendocrine neoplasms. *Cancers (Basel)* 2021;**13**(5):962.

235. Jaiswal SK, Sarathi V, Memon SS, et al. Sympathetic paraganglioma: a single-center experience from Western India. *Endocr Pract* 2019;**25**(3):211-9.

236. Jansen TJP, van Lith SAM, Boss M, et al. Exendin-4 analogs in insulinoma theranostics. *J Labelled Comp Radiopharm* 2019;**62**(10):656-72.

237. Jha A, Patel M, Baker E, et al. Role of (68)Ga-DOTATATE PET/CT in a case of SDHB-related pterygopalatine fossa paraganglioma successfully controlled with octreotide. *Nucl Med Mol Imaging* 2020;**54**(1):48-52.

238. Jiménez-Fonseca P, Carmona-Bayonas A, Martín-Pérez E, et al. Health-related quality of life in well-differentiated metastatic gastroenteropancreatic neuroendocrine tumors. *Cancer Metastasis Rev* 2015;**34**(3):381-400.

239. Jiménez-Franco LD, Glatting G, Prasad V, Weber WA, Beer AJ, Kletting P. Effect of tumor perfusion and receptor density on tumor control probability in (177)Lu-DOTATATE therapy: an in silico analysis for standard and optimized treatment. *J Nucl Med* 2021;**62**(1):92-8.

240. Jin XF, Auernhammer CJ, Ilhan H, et al. Combination of 5-fluorouracil with epigenetic modifiers induces radiosensitization, somatostatin receptor 2 expression, and radioligand binding in neuroendocrine tumor cells in vitro. *J Nucl Med* 2019;**60**(9):1240-6.

241. Jin XF, Spampatti MP, Spitzweg C, Auernhammer CJ. Supportive therapy in gastroenteropancreatic neuroendocrine tumors: Often forgotten but important. *Rev Endocr Metab Disord* 2018;**19**(2):145-58.

242. Johnbeck CB, Mortensen J. Somatostatin receptor imaging PET in neuroendocrine neoplasm. *PET Clin* 2021;**16**(2):191-203.

243. Kaemmerer D, Prasad V, Daffner W, et al. Neoadjuvant peptide receptor radionuclide therapy for an inoperable neuroendocrine pancreatic tumor. *World J Gastroenterol* 2009;**15**(46):5867-70.

244. Kaemmerer D, Wirtz RM, Fischer EK, et al. Analysis of somatostatin receptor 2A immunohistochemistry, RT-qPCR, and in vivo PET/CT data in patients with pancreatic neuroendocrine neoplasm. *Pancreas* 2015;**44**(4):648-54.

245. Kaewput C, Vinjamuri S. Comparison of renal uptake of 68Ga-DOTANOC PET/CT and estimated glomerular filtration rate before and after peptide receptor radionuclide therapy in patients with metastatic neuroendocrine tumours. *Nucl Med Commun* 2016;**37**(12):1325-32.

246. Kairemo K, Kangasmäki A. 4D SPECT/CT acquisition for 3D dose calculation and dose planning in (177)Lu-peptide receptor radionuclide therapy: applications for clinical routine. *Recent Results Cancer Res* 2013;**194**:537-50.

247. Kalshetty A, Ramaswamy A, Ostwal V, Basu S. Resistant functioning and/or progressive symptomatic metastatic gastroenteropancreatic neuroendocrine tumors: efficacy of 177Lu-DOTATATE peptide receptor radionuclide therapy in this setting. *Nucl Med Commun* 2018;**39**(12):1143-9.

248. Kameswaran M, Pandey U, Gamre N, Vimalnath KV, Sarma HD, Dash A. Evaluation of (177)Lu-CHX-A"-DTPA-bevacizumab as a radioimmunotherapy agent targeting VEGF expressing cancers. *Appl Radiat Isot* 2016;**114**:196-201.

249. Kamp K, Gumz B, Feelders RA, et al. Safety and efficacy of everolimus in gastrointestinal and pancreatic neuroendocrine tumors after (177)Lu-octreotate. *Endocr Relat Cancer* 2013;**20**(6):825-31.

250. Karfis I, Marin G, Machiels G, Hendlisz A, Flamen P. Acute pancreatitis following peptide receptor radionuclide therapy: an unusual adverse event. *Clin Nucl Med* 2018;**43**(7):e232-e3.

251. Kasi PM, Maige CL, Shahjehan F, et al. A care process model to deliver (177)Lu-Dotatate peptide receptor radionuclide therapy for patients with neuroendocrine tumors. *Front Oncol* 2018;**8**:663.

252. Kasi PM, Sharma A, Jain MK. Expanding the indication for novel theranostic 177Lu-Dotatate peptide receptor radionuclide therapy: proof-of-concept of PRRT in Merkel cell cancer. *Case Rep Oncol* 2019;**12**(1):98-103.

253. Kawase T, Kubota K, Takeda Y, et al. Additional external radiotherapy to multiple metastases originating from thymic neuroendocrine tumor following peptide receptor radionuclide therapy: a case report [article in Japanese]. *Kaku Igaku* 2014;**51**(2):47-53.

254. Kendi AT, Halfdanarson TR, Packard A, Dundar A, Subramaniam RM. Therapy with (177)Lu-DOTATATE: clinical implementation and impact on care of patients with neuroendocrine tumors. *AJR Am J Roentgenol* 2019;**213**(2):309-17.

255. Kesavan M, Claringbold PG, Turner JH. Hematological toxicity of combined 177Lu-octreotate radiopeptide chemotherapy of gastroenteropancreatic neuroendocrine tumors in long-term follow-up. *Neuroendocrinology* 2014;**99**(2):108-17.

256. Keutgen XM, Schadde E, Pommier RF, Halfdanarson TR, Howe JR, Kebebew E. Metastatic neuroendocrine tumors of the gastrointestinal tract and pancreas: A surgeon's plea to centering attention on the liver. *Semin Oncol* 2018;**45**(4):232-5.

257. Kiesewetter B, Raderer M. How I treat neuroendocrine tumours. *ESMO Open* 2020;**5**(4):e000811.

258. Kim C, Liu SV, Subramaniam DS, et al. Phase I study of the (177)Lu-DOTA(0)-Tyr(3)-Octreotate (lutathera) in combination with nivolumab in patients with neuroendocrine tumors of the lung. *J Immunother Cancer* 2020;**8**(2):e000980.

259. Kim MR, Shim HK. Long-term follow-up of a patient with primary presacral neuroendocrine tumor: a case report with literature review. *Am J Case Rep* 2019;**20**:1969-75.

260. Kinney RE, Decker R, Sundlof D, Rizvi MA, Schadler K. Case Report: Neuroendocrine tumor with cardiac metastasis. *Front Cardiovasc Med* 2020;**7**:596921.

261. Kiňová S, Kováčová M, Čaprnda M, Koreň M. Management of treatment in patients with neuroendocrine neoplasmas of digestive tract [article in Slovak]. *Vnitr Lek* 2015;**61**(Suppl 5):12-20.

262. Kipnis ST, Hung M, Kumar S, et al. Laboratory, clinical, and survival outcomes associated with peptide receptor radionuclide therapy in patients with gastroenteropancreatic neuroendocrine tumors. *JAMA Netw Open* 2021;**4**(3):e212274.

263. Kletting P, Kull T, Maaß C, et al. Optimized peptide amount and activity for ^{90}Y-labeled DOTATATE therapy. *J Nucl Med* 2016;**57**(4):503-8.

264. Kletting P, Muller B, Erentok B, et al. Differences in predicted and actually absorbed doses in peptide receptor radionuclide therapy. *Med Phys* 2012;**39**(9):5708-17.

265. Koffas A, Toumpanakis C. Comparative safety review of the current therapies for gastroenteropancreatic neuroendocrine tumors. *Expert Opin Drug Saf* 2021;**20**(3):321-34.

266. Kolasińska-Ćwikła A, Łowczak A, Maciejkiewicz KM, Ćwikła JB. Peptide receptor radionuclide therapy for advanced gastroenteropancreatic neuroendocrine tumors - from oncology perspective. *Nucl Med Rev Cent East Eur* 2018;**21**(2). doi: 10.5603/NMR.2018.0019.

267. Kolasinska-Ćwikła A, Pęczkowska M, Ćwikła JB, et al. A clinical efficacy of PRRT in patients with advanced, nonresectable, paraganglioma-pheochromocytoma, related to SDHx gene mutation. *J Clin Med* 2019;**8**(7):952.

268. Kong G, Callahan J, Hofman MS, et al. High clinical and morphologic response using (90)Y-DOTA-octreotate sequenced with (177)Lu-DOTA-octreotate induction peptide receptor chemoradionuclide therapy (PRCRT) for bulky neuroendocrine tumours. *Eur J Nucl Med Mol Imaging* 2017;**44**(3):476-89.

269. Kong G, Grozinsky-Glasberg S, Hofman MS, et al. Highly favourable outcomes with peptide receptor radionuclide therapy (PRRT) for metastatic rectal neuroendocrine neoplasia (NEN). *Eur J Nucl Med Mol Imaging* 2019;**46**(3):718-27.

270. Kong G, Hicks RJ. Peptide receptor radiotherapy: current approaches and future directions. *Curr Treat Options Oncol* 2019;**20**(10):77.

271. Kos-Kudła B, Bolanowski M, Hubalewska-Dydejczyk A, et al. Pancreatic endocrine tumors - management guidelines (recommended by the Polish Network of Neuroendocrine Tumors) [article in Polish]. *Endokrynol Pol* 2008;**59**(1):68-86.

272. Kos-Kudła B, Hubalewska-Dydejczyk A, Kuśnierz K, et al. Pancreatic neuroendocrine neoplasms - management guidelines (recommended by the Polish Network of Neuroendocrine Tumours). *Endokrynol Pol* 2013;**64**(6):459-79.

273. Kos-Kudła B, Rosiek V, Borowska M, et al. Pancreatic neuroendocrine neoplasms - management guidelines (recommended by the Polish Network of Neuroendocrine Tumours). *Endokrynol Pol* 2017;**68**(2):169-97.

274. Kováčová M, Filková M, Potočárová M, Kiňová S, Pajvani UB. Calcitonin-secreting pancreatic neuroendocrine tumors: a case report and review of the literature. *Endocr Pract* 2014;**20**(8):e140-4.

275. Kovacs L, Tassano M, Cabrera M, et al. Development of (177)Lu-DOTA-dendrimer and determination of its effect on metal and ion levels in tumor tissue. *Cancer Biother Radiopharm* 2015;**30**(10):405-9.

276. Krebs S, O'Donoghue JA, Biegel E, et al. Comparison of (68)Ga-DOTA-JR11 PET/CT with dosimetric (177)Lu-satoreotide tetraxetan ((177)Lu-DOTA-JR11) SPECT/CT in patients with metastatic neuroendocrine tumors undergoing peptide receptor radionuclide therapy. *Eur J Nucl Med Mol Imaging* 2020;**47**(13):3047-57.

277. Krenning EP, de Jong M, Kooij PP, et al. Radiolabelled somatostatin analogue(s) for peptide receptor scintigraphy and radionuclide therapy. *Ann Oncol* 1999;**10**(Suppl 2):S23-9.

278. Krenning EP, Valkema R, Kooij PP, et al. The role of radioactive somatostatin and its analogues in the control of tumor growth. *Recent Results Cancer Res* 2000;**153**:1-13.

279. Krenning EP, Valkema R, Kooij PP, et al. Scintigraphy and radionuclide therapy with [indium-111-labelled-diethyl triamine penta-acetic acid-D-Phe1]-octreotide. *Ital J Gastroenterol Hepatol* 1999;**31**(Suppl 2):S219-23.

280. Krenning EP, Valkema R, Kwekkeboom DJ, et al. Molecular imaging as in vivo molecular pathology for gastroenteropancreatic neuroendocrine tumors: implications for follow-up after therapy. *J Nucl Med* 2005;**46**(Suppl 1):76s-82s.

281. Krishnaraju VS, Satapathy S, Sood A, et al. Expression of CXCR4 receptors in refractory atypical mediastinal carcinoid. *Clin Nucl Med* 2020;**45**(9):e422-e4.

282. Kristiansson A, Ahlstedt J, Holmqvist B, et al. Protection of kidney function with human antioxidation protein α(1)-microglobulin in a mouse (177)Lu-DOTATATE radiation therapy model. *Antioxid Redox Signal* 2019;**30**(14):1746-59.

283. Kroiss A, Putzer D, Decristoforo C, et al. 68Ga-DOTA-TOC uptake in neuroendocrine tumour and healthy tissue: differentiation of physiological uptake and pathological processes in PET/CT. *Eur J Nucl Med Mol Imaging* 2013;**40**(4):514-23.

284. Kucukyurt S, Yagiz Ozogul Y, Ercaliskan A, Kabasakal L, Eskazan AE. Therapy-related chronic myeloid leukemia in a patient receiving peptide receptor radionuclide therapy for pancreatic neuroendocrine tumor. *Cancer Rep (Hoboken)* 2020;**3**(5):e1282.

285. Kulkarni HR, Baum RP. Patient selection for personalized peptide receptor radionuclide therapy using Ga-68 somatostatin receptor PET/CT. *PET Clin* 2014;**9**(1):83-90.

286. Kulke MH, Benson AB, Dasari A, et al. Real-world treatment patterns and clinical outcomes in advanced Gastrointestinal Neuroendocrine Tumors (GI NET): a multicenter retrospective chart review study. *Oncologist* 2019;**24**(8):1056-65.

287. Kunikowska J, Bajera A, Sawicka M, et al. Different technical possibilities of post-therapeutic tandem 90Y//177Lu-DOTATATE imaging. *Nucl Med Rev Cent East Eur* 2013;**16**(2):70-4.

288. Kunikowska J, Królicki L, Cwikła J, et al. [Radioisotope therapy with somatostatin analogues in neuroendocrine tumours (case report)]. *Endokrynol Pol* 2005;**56**(1):46-54.

289. Kunikowska J, Królicki L, Hubalewska-Dydejczyk A, Mikołajczak R, Sowa-Staszczak A, Pawlak D. Clinical results of radionuclide therapy of neuroendocrine tumours with 90Y-DOTATATE and tandem 90Y/177Lu-DOTATATE: which is a better therapy option? *Eur J Nucl Med Mol Imaging* 2011;**38**(10):1788-97.

290. Kunikowska J, Królicki L, Sowa-Staszczak A, Pawlak D, Hubalewska-Dydejczyk A, Mikołajczak R. Nephrotoxicity after PRRT - still a serious clinical problem? Renal toxicity after peptide receptor radionuclide therapy with 90Y-DOTATATE and 90Y/177Lu-DOTATATE. *Endokrynol Pol* 2013;**64**(1):13-20.

291. Kunikowska J, Pawlak D, Bąk MI, Kos-Kudła B, Mikołajczak R, Królicki L. Long-term results and tolerability of tandem peptide receptor radionuclide therapy with (90)Y/(177)Lu-DOTATATE in neuroendocrine tumors with respect to the primary location: a 10-year study. *Ann Nucl Med* 2017;**31**(5):347-56.

292. Kunikowska J, Zemczak A, Górska M, et al. TeleNEN as a telemedicine model for neuroendocrine neoplasm management in case of Meckel's diverticulum NET. *Endokrynol Pol* 2018;**69**(3):313-7.

293. Kunikowska J, Zemczak A, Kołodziej M, et al. Tandem peptide receptor radionuclide therapy using (90)Y/(177)Lu-DOTATATE for neuroendocrine tumors efficacy and side-effects - Polish multicenter experience. *Eur J Nucl Med Mol Imaging* 2020;**47**(4):922-33.

294. Kwekkeboom D, Krenning EP, de Jong M. Peptide receptor imaging and therapy. *J Nucl Med* 2000;**41**(10):1704-13.

295. Kwekkeboom DJ, de Herder WW, Kam BL, et al. Treatment with the radiolabeled somatostatin analog [177 Lu-DOTA 0,Tyr3]octreotate: toxicity, efficacy, and survival. *J Clin Oncol* 2008;**26**(13):2124-30.

296. Kwekkeboom DJ, de Herder WW, Krenning EP. Somatostatin receptor-targeted radionuclide therapy in patients with gastroenteropancreatic neuroendocrine tumors. *Endocrinol Metab Clin North Am* 2011;**40**(1):173-85, ix.

297. Kwekkeboom DJ, de Herder WW, van Eijck CH, et al. Peptide receptor radionuclide therapy in patients with gastroenteropancreatic neuroendocrine tumors. *Semin Nucl Med* 2010;**40**(2):78-88.

298. Kwekkeboom DJ, Kam BL, van Essen M, et al. Somatostatin-receptor-based imaging and therapy of gastroenteropancreatic neuroendocrine tumors. *Endocr Relat Cancer* 2010;**17**(1): R53-73.

299. Kwekkeboom DJ, Krenning EP. Somatostatin receptor imaging. *Semin Nucl Med* 2002;**32**(2):84-91.

300. Kwekkeboom DJ, Mueller-Brand J, Paganelli G, et al. Overview of results of peptide receptor radionuclide therapy with 3 radiolabeled somatostatin analogs. *J Nucl Med* 2005;**46**(Suppl 1):62s-6s.

301. Kwekkeboom DJ, Teunissen JJ, Bakker WH, et al. Radiolabeled somatostatin analog [177Lu-DOTA0,Tyr3]octreotate in patients with endocrine gastroenteropancreatic tumors. *J Clin Oncol* 2005;**23**(12):2754-62.

302. Ladwa R, Wen Hong H, Wyld D, Pattison DA, Burge M. Tumor cystic necrosis following peptide receptor radionuclide therapy in neuroendocrine tumors. *Clin Nucl Med* 2018;**43**(3):186-7.

303. Lamarca A, Cives M, de Mestier L, et al. Advanced small-bowel well-differentiated neuroendocrine tumours: An international survey of practice on 3(rd)-line treatment. *World J Gastroenterol* 2021;**27**(10):976-89.

304. Lamarca A, Pritchard DM, Westwood T, et al. 68Gallium DOTANOC-PET imaging in lung carcinoids: impact on patients' management. *Neuroendocrinology* 2018;**106**(2):128-38.

305. Langsteger W, Rezaee A, Loidl W, et al. 32nd International Austrian Winter Symposium: Zell am See, the Netherlands. 20-23 January 2016. *EJNMMI Res* 2016;**6**(Suppl 1):32.

306. Lapa C, Kircher M, Hänscheid H, et al. Peptide receptor radionuclide therapy as a new tool in treatment-refractory sarcoidosis - initial experience in two patients. *Theranostics* 2018;**8**(3):644-9.

307. Larouche V, Akirov A, Alshehri S, Ezzat S. Management of small bowel neuroendocrine tumors. *Cancers (Basel)* 2019;**11**(9):1395.

308. Lawhn-Heath C, Fidelman N, Chee B, et al. Intraarterial peptide receptor radionuclide therapy using (90)Y-DOTATOC for hepatic metastases of neuroendocrine tumors. *J Nucl Med* 2021;**62**(2):221-7.

309. Leeuwenkamp O, Smith-Palmer J, Ortiz R, et al. Cost-effectiveness of Lutetium [(177)Lu] oxodotreotide versus best supportive care with octreotide in patients with midgut neuroendocrine tumors in France. *J Med Econ* 2020;**23**(12):1534-41.

310. Lenain R, Hamroun A, Lion G, et al. Description of a transient proximal tubulopathy induced by amino acids perfusion in peptide receptor radionuclide therapy: a case report. *Medicine (Baltimore)* 2019;**98**(52):e18478.

311. Leonhäuser B, Happel C, Gröner D, et al. Evaluation of intratherapeutic 177Lu-HA-DOTATATE treatment in neuroendocrine tumors: dosimetry with SPECT, whole-body imaging and gamma probe [in German]. *Nuklearmedizin* 2019;**58**(5):379-86.

312. Leung K. (68)Ga-1,4,7,10-Tetraazacyclododecane-1,4,7,10-tetraacetic acid-p-Cl-Phe-cyclo(D-Cys-Tyr-D-4-amino-Phe(carbamoyl)-Lys-Thr-Cys)D-Tyr-NH(2). Molecular Imaging and Contrast Agent Database (MICAD). Bethesda (MD): National Center for Biotechnology Information (US); 2004.

313. Leung K. (68)Ga-1,4,7-Triazacyclononane,1-glutaric acid-4,7-acetic acid-p-Cl-Phe-cyclo(D-Cys-Tyr-D-4-amino-Phe(carbamoyl)-Lys-Thr-Cys)D-Tyr-NH(2). Molecular Imaging and Contrast Agent Database (MICAD). Bethesda (MD): National Center for Biotechnology Information (US); 2004.

314. Leung K. (64)Cu-4,11-Bis(carboxymethyl)-1,4,8,11-tetraazabicyclo[6.6.2]hexadecane-p-Cl-Phe-cyclo(D-Cys-Tyr-D-4-amino-Phe(carbamoyl)-Lys-Thr-Cys)D-Tyr-NH(2). Molecular Imaging and Contrast Agent Database (MICAD). Bethesda (MD): National Center for Biotechnology Information (US); 2004.

315. Leung K. (64)Cu-1,4,7-Triazacyclononane,1-glutaric acid-4,7-acetic acid-p-Cl-Phe-cyclo(D-Cys-Tyr-D-4-amino-Phe(carbamoyl)-Lys-Thr-Cys)D-Tyr-NH(2). Molecular Imaging and Contrast Agent Database (MICAD). Bethesda (MD): National Center for Biotechnology Information (US); 2004.

316. Leung K. Octreotide conjugated to pegylated ultrasmall superparamagnetic iron oxide nanoparticle. Molecular Imaging and Contrast Agent Database (MICAD). Bethesda (MD): National Center for Biotechnology Information (US); 2004.

317. Leung K. (111)In-Tetraazacyclododecane-N,N',N'',N'''-tetraacetic acid-Phe(4-NO(2))-cyclo(D-Cys-Tyr-D-Trp-Lys-Thr-Cys)-D-Tyr-NH(2). Molecular Imaging and Contrast Agent Database (MICAD). Bethesda (MD): National Center for Biotechnology Information (US); 2004.

318. Leung K. (64)Cu-4,11-Bis(carboxymethyl)-1,4,8,11-tetraazabicyclo[6.6.2]hexadecane-Phe(4-NO(2))-cyclo(D-Cys-Tyr-D-Trp-Lys-Thr-Cys)-D-Tyr-NH(2). Molecular Imaging and Contrast Agent Database (MICAD). Bethesda (MD): National Center for Biotechnology Information (US); 2004.

319. Leung K. (111)In-1,4,7,10-Tetraazacyclododecane-N,N',N'',N'''-tetraacetic acid-cyclo(D-diaminobutyric acid-Arg-Phe-Phe-D-Trp-Lys-Thr-Phe). Molecular Imaging and Contrast Agent Database (MICAD). Bethesda (MD): National Center for Biotechnology Information (US); 2004.

320. Leung K. (64)Cu-1,4,8,11-Tetraazacyclotetradecane-N,N',N'',N'''-tetraacetic acid-D-Phe-cyclo(Cys-Tyr-D-Trp-Lys-Thr-Cys)-Thr(OH). Molecular Imaging and Contrast Agent Database (MICAD). Bethesda (MD): National Center for Biotechnology Information (US); 2004.

321. Leung K. (64)Cu-4,11-Bis(carboxymethyl)-1,4,8,11-tetraazabicyclo[6.6.2]hexadecane-D-Phe-cyclo(Cys-Tyr-D-Trp-Lys-Thr-Cys)-Thr(OH). Molecular Imaging and Contrast Agent Database (MICAD). Bethesda (MD): National Center for Biotechnology Information (US); 2004.

322. Leung K. (111)In-Tetraazacyclododecane-N,N',N'',N'''-tetraacetic acid-c(D-Cys-Phe-Tyr-D-AgI(8)(Me,2-naphthoyl)-Lys-Thr-Phe-Cys)-OH. Molecular Imaging and Contrast Agent Database (MICAD). Bethesda (MD): National Center for Biotechnology Information (US); 2004.

323. Leyden S, Kolarova T, Bouvier C, et al. Unmet needs in the international neuroendocrine tumor (NET) community: assessment of major gaps from the perspective of patients, patient advocates and NET health care professionals. *Int J Cancer* 2020;**146**(5):1316-23.

324. Liberini V, Huellner MW, Grimaldi S, et al. The challenge of evaluating response to peptide receptor radionuclide therapy in gastroenteropancreatic neuroendocrine tumors: the present and the future. *Diagnostics (Basel)* 2020;**10**(12):1083.

325. Liberini V, Rampado O, Gallio E, et al. (68)Ga-DOTATOC PET/CT-based radiomic analysis and prrt outcome: a preliminary evaluation based on an exploratory radiomic analysis on two patients. *Front Med (Lausanne)* 2020;**7**:601853.

326. Lim JC, Hong YD, Kim JJ, Choi SM, Baek HS, Choi SJ. Synthesis and biological evaluation of a novel (177)Lu-DOTA-[Gly(3)-cyclized(Dap(4), (d)-Phe(7), Asp(10))-Arg(11)]α-MSH(3-13) analogue for melanocortin-1 receptor-positive tumor targeting. *Cancer Biother Radiopharm* 2012;**27**(8):464-72.

327. Limouris GS, Karfis I, Chatzioannou A, et al. Super-selective hepatic arterial infusions as established technique ('ARETAIEION' Protocol) of [177Lu]DOTA-TATE in inoperable neuroendocrine liver metastases of gastro-entero-pancreatic (GEP) tumors. *Q J Nucl Med Mol Imaging* 2012;**56**(6):551-8.

328. Lisova K, Sergeev M, Evans-Axelsson S, et al. Microscale radiosynthesis, preclinical imaging and dosimetry study of [(18)F]AMBF(3)-TATE: a potential PET tracer for clinical imaging of somatostatin receptors. *Nucl Med Biol* 2018;**61**:36-44.

329. Liu C, Liu T, Zhang J, Baum RP, Yang Z. Excellent response to 177Lu-DOTATATE peptide receptor radionuclide therapy in a patient with progressive metastatic castration-resistant prostate cancer with neuroendocrine differentiation after 177Lu-PSMA therapy. *Clin Nucl Med* 2019;**44**(11):876-8.

330. Liu F, Zhu H, Yu J, et al. (68)Ga/(177)Lu-labeled DOTA-TATE shows similar imaging and biodistribution in neuroendocrine tumor model. *Tumour Biol* 2017;**39**(6):1010428317705519.

331. Liu Q, Zang J, Sui H, et al. Peptide receptor radionuclide therapy of late-stage neuroendocrine tumor patients with multiple cycles of (177)Lu-DOTA-EB-TATE. *J Nucl Med* 2021;**62**(3):386-92.

332. Liu Q, Zhang J, Kulkarni HR, Baum RP. 177Lu-DOTATOC peptide receptor radionuclide therapy in a patient with neuroendocrine breast carcinoma and breast invasive ductal carcinoma. *Clin Nucl Med* 2020;**45**(5):e232-e5.

333. Lorenzoni A, Capozza A, Artale S, Maccauro M, Seregni EC. Impressive response to tandem treatment with [90Y]DOTATOC and [177Lu]DOTATOC in grade 3 pancreatic neuroendocrine carcinoma. *Clin Nucl Med* 2018;**43**(7):506-8.

334. Loughrey PB, Zhang D, Heaney AP. New treatments for the carcinoid syndrome. *Endocrinol Metab Clin North Am* 2018;**47**(3):557-76.

335. Lubberink M, Wilking H, Öst A, et al. In vivo instability of (177)Lu-DOTATATE during peptide receptor radionuclide therapy. *J Nucl Med* 2020;**61**(9):1337-40.

336. Lundsten S, Spiegelberg D, Raval NR, Nestor M. The radiosensitizer Onalespib increases complete remission in (177)Lu-DOTATATE-treated mice bearing neuroendocrine tumor xenografts. *Eur J Nucl Med Mol Imaging* 2020;**47**(4):980-90.

337. Lundsten S, Spiegelberg D, Stenerlöw B, Nestor M. The HSP90 inhibitor onalespib potentiates 177Lu-DOTATATE therapy in neuroendocrine tumor cells. *Int J Oncol* 2019;**55**(6):1287-95.

338. Lybik N, Wale DJ, Wong KK, Liao E, Viglianti BL. 68Ga-DOTATATE PET/CT imaging of refractory pituitary macroadenoma invading the orbit. *Clin Nucl Med* 2021;**46**(6):505-6.

339. Maaß C, Rivas JR, Attarwala AA, et al. Physiologically based pharmacokinetic modeling of (18)F-SiFAlin-Asp3-PEG1-TATE in AR42J tumor bearing mice. *Nucl Med Biol* 2016;**43**(4):243-6.

340. Maaß C, Sachs JP, Hardiansyah D, Mottaghy FM, Kletting P, Glatting G. Dependence of treatment planning accuracy in peptide receptor radionuclide therapy on the sampling schedule. *EJNMMI Res* 2016;**6**(1):30.

341. Maccauro M, Follacchio GA, Spreafico C, Coppa J, Seregni E. Safety and efficacy of combined peptide receptor radionuclide therapy and liver selective internal radiation therapy in a patient with metastatic neuroendocrine tumor. *Clin Nucl Med* 2019;**44**(4):e286-e8.

342. Magalhães D, Sampaio IL, Ferreira G, et al. Peptide receptor radionuclide therapy with (177)Lu-DOTA-TATE as a promising treatment of malignant insulinoma: a series of case reports and literature review. *J Endocrinol Invest* 2019;**42**(3):249-60.

343. Mahajan S, O'Donoghue J, Weber W, Bodei L. Integrating early rapid post-peptide receptor radionuclide therapy quality assurance scan into the outpatient setting. *J Nucl Med Radiat Ther* 2019;**10**(1):395.

344. Mair C, Warwitz B, Fink K, et al. Radiation exposure after (177)Lu-DOTATATE and (177)Lu-PSMA-617 therapy. *Ann Nucl Med* 2018;**32**(7):499-502.

345. Makis W, McCann K, Buteau FA, McEwan AJ. Resolution of malignant ascites and stabilization of metastases in a patient with small bowel neuroendocrine tumor with 177Lu-DOTATATE following progression after 17 131I-MIBG treatments and chemotherapy. *Clin Nucl Med* 2015;**40**(7):564-6.

346. Makis W, McCann K, Buteau FA, McEwan AJ. Liver and bone metastases from small bowel neuroendocrine tumor respond to 177Lu-DOTATATE induction and maintenance therapies. *Clin Nucl Med* 2015;**40**(2):162-5.

347. Makis W, McCann K, McEwan AJ. Incidental acinic cell carcinoma of the parotid gland discordant on 123I-MIBG, 111In-octreotide, and 18F-FDG PET/CT in a patient with neuroendocrine tumor of the cecum. *Clin Nucl Med* 2015;**40**(8):676-8.

348. Makis W, McCann K, McEwan AJ. Metastatic insulinoma pancreatic neuroendocrine tumor treated with 177Lu-DOTATATE induction and maintenance peptide receptor radionuclide therapy: a suggested protocol. *Clin Nucl Med* 2016;**41**(1):53-4.

349. Makis W, McCann K, Riauka TA, McEwan AJ. Resolution of hyperreninemia, secondary hyperaldosteronism, and hypokalemia with 177Lu-DOTATATE induction and maintenance peptide receptor radionuclide therapy in a patient with pancreatic neuroendocrine tumor. *Clin Nucl Med* 2015;**40**(11):880-4.

350. Makis W, McCann K, Riauka TA, McEwan AJ. Glucagonoma pancreatic neuroendocrine tumor treated with 177Lu DOTATATE induction and maintenance peptide receptor radionuclide therapy. *Clin Nucl Med* 2015;**40**(11):877-9.

351. Makis W, McCann K, Riauka TA, McEwan AJ. Ectopic corticotropin-producing neuroendocrine tumor of the pancreas treated with 177Lu DOTATATE induction and maintenance peptide receptor radionuclide therapy. *Clin Nucl Med* 2016;**41**(1):50-2.

352. Malaza G, Brofferio A, Lin F, Pacak K. Ivabradine in catecholamine-induced tachycardia in a patient with paraganglioma. *N Engl J Med* 2019;**380**(13):1284-6.

353. Malczewska A, Bodei L, Kidd M, Modlin IM. Blood mRNA measurement (NETest) for neuroendocrine tumor diagnosis of image-negative liver metastatic disease. *J Clin Endocrinol Metab* 2019;**104**(3):867-72.

354. Mallón Araujo MDC, Abou Jokh Casas E, Abou Jokh Casas C, Pubul Núñez V. Added-value of molecular imaging in myocardial metastasis of an ileal neuroendocrine tumour treated with peptide receptor radionuclide therapy: a case report. *Eur Heart J Case Rep* 2021;**5**(2):ytaa550.

355. Malo ME, Allen KJH, Jiao R, Frank C, Rickles D, Dadachova E. Mechanistic insights into synergy between melanin-targeting radioimmunotherapy and immunotherapy in experimental melanoma. *Int J Mol Sci* 2020;**21**(22):8721.

356. Mansi R, Fani M. Design and development of the theranostic pair (177) Lu-OPS201/(68) Ga-OPS202 for targeting somatostatin receptor expressing tumors. *J Labelled Comp Radiopharm* 2019;**62**(10):635-45.

357. Mansi R, Nicolas GP, Del Pozzo L, Abid KA, Grouzmann E, Fani M. Evaluation of a new (177)Lu-labeled somatostatin analog for the treatment of tumors expressing somatostatin receptor subtypes 2 and 5. *Molecules* 2020;**25**(18):4155.

358. Marciniak A, Brasuń J. Somatostatin analogues labeled with copper radioisotopes: current status. *J Radioanal Nucl Chem* 2017;**313**(2):279-89.

359. Marin G, Vanderlinden B, Karfis I, et al. A dosimetry procedure for organs-at-risk in (177)Lu peptide receptor radionuclide therapy of patients with neuroendocrine tumours. *Phys Med* 2018;**56**:41-9.

360. Marinova M, Mücke M, Fischer F, et al. Quality of life in patients with midgut NET following peptide receptor radionuclide therapy. *Eur J Nucl Med Mol Imaging* 2019;**46**(11):2252-9.

361. Marinova M, Mücke M, Mahlberg L, et al. Improving quality of life in patients with pancreatic neuroendocrine tumor following peptide receptor radionuclide therapy assessed by EORTC QLQ-C30. *Eur J Nucl Med Mol Imaging* 2018;**45**(1):38-46.

362. Martini C, Buxbaum S, Rodrigues M, et al. Quality of life in patients with metastatic gastroenteropancreatic neuroendocrine tumors receiving peptide receptor radionuclide therapy: information from a monitoring program in clinical routine. *J Nucl Med* 2018;**59**(10):1566-73.

363. Marx SJ. Recent topics around multiple endocrine neoplasia type 1. *J Clin Endocrinol Metab* 2018;**103**(4):1296-301.

364. Mathur A, Prashant V, Sakhare N, et al. Bulk scale formulation of therapeutic doses of clinical grade ready-to-use (177)Lu-DOTA-TATE: the intricate radiochemistry aspects. *Cancer Biother Radiopharm* 2017;**32**(7):266-73.

365. Matuchansky C. 177Lu-Dotatate for midgut neuroendocrine tumors. *N Engl J Med* 2017;**376**(14):1391.

366. Maughan NM, Kim H, Hao Y, et al. Initial experience and lessons learned with implementing Lutetium-177-dotatate radiopharmaceutical therapy in a radiation oncology-based program. *Brachytherapy* 2021;**20**(1):237-47.

367. McElroy KM, Binkovitz LA, Trout AT, et al. Pediatric applications of Dotatate: early diagnostic and therapeutic experience. *Pediatr Radiol* 2020;**50**(7):882-97.

368. Mehrvarz Sarshekeh A, Halperin DM, Dasari A. Update on management of midgut neuroendocrine tumors. *Int J Endocr Oncol* 2016;**3**(2):175-89.

369. Meirovitz A, Shouchane-Blum K, Maly A, et al. The potential of somatostatin receptor 2 as a novel therapeutic target in salivary gland malignant tumors. *J Cancer Res Clin Oncol* 2021;**147**(5):1335-40.

370. Melis M, Forrer F, Capello A, et al. Up-regulation of somatostatin receptor density on rat CA20948 tumors escaped from low dose [177Lu-DOTA0,Tyr3]octreotate therapy. *Q J Nucl Med Mol Imaging* 2007;**51**(4):324-33.

371. Melis M, Kaemmerer D, de Swart J, et al. Localization of radiolabeled somatostatin analogs in the spleen. *Clin Nucl Med* 2016;**41**(2):e111-4.

372. Menda Y, Madsen MT, O'Dorisio TM, et al. (90)Y-DOTATOC dosimetry-based personalized peptide receptor radionuclide therapy. *J Nucl Med* 2018;**59**(11):1692-8.

373. Menda Y, O'Dorisio MS, Kao S, et al. Phase I trial of 90Y-DOTATOC therapy in children and young adults with refractory solid tumors that express somatostatin receptors. *J Nucl Med* 2010;**51**(10):1524-31.

374. Mercado-Asis LB, Wolf KI, Jochmanova I, Taïeb D. Pheochromocytoma: a genetic and diagnostic update. *Endocr Pract* 2018;**24**(1):78-90.

375. Merola E, Capurso G, Campana D, et al. Acute leukaemia following low dose peptide receptor radionuclide therapy for an intestinal carcinoid. *Dig Liver Dis* 2010;**42**(6):457-8.

376. Merola E, Prasad V, Pascher A, et al. Peritoneal carcinomatosis in gastro-entero-pancreatic neuroendocrine neoplasms: clinical impact and effectiveness of the available therapeutic options. *Neuroendocrinology* 2020;**110**(6):517-24.

377. Metz DC, Jensen RT. Gastrointestinal neuroendocrine tumors: pancreatic endocrine tumors. *Gastroenterology* 2008;**135**(5):1469-92.

378. Miao Y, Fisher DR, Quinn TP. Reducing renal uptake of 90Y- and 177Lu-labeled alpha-melanocyte stimulating hormone peptide analogues. *Nucl Med Biol* 2006;**33**(6):723-33.

379. Miao Y, Hoffman TJ, Quinn TP. Tumor-targeting properties of 90Y- and 177Lu-labeled alpha-melanocyte stimulating hormone peptide analogues in a murine melanoma model. *Nucl Med Biol* 2005;**32**(5):485-93.

380. Miao Y, Shelton T, Quinn TP. Therapeutic efficacy of a 177Lu-labeled DOTA conjugated alpha-melanocyte-stimulating hormone peptide in a murine melanoma-bearing mouse model. *Cancer Biother Radiopharm* 2007;**22**(3):333-41.

381. Miederer M, Henriksen G, Alke A, et al. Preclinical evaluation of the alpha-particle generator nuclide 225Ac for somatostatin receptor radiotherapy of neuroendocrine tumors. *Clin Cancer Res* 2008;**14**(11):3555-61.

382. Miederer M, Reber H, Helisch A, Fottner C, Weber M, Schreckenberger M. One single-time-point kidney uptake from OctreoScan correlates with number of desintegrations measured over 72 hours and calculated for the 6.7 hours half-life nuclide (177)Lu. *Clin Nucl Med* 2012;**37**(10):e245-8.

383. Minutoli F, Herberg A, Sindoni A, Cardile D, Cucinotta M, Baldari S. Is detection of additional lesions in post-peptide receptor radionuclide therapy scans with respect to diagnostic imaging only due to different affinity of ligands?: a report of discordance between diagnostic and posttherapy imaging using the same ligand. *Clin Nucl Med* 2012;**37**(8):817-8.

384. Minutoli F, Herberg A, Sindoni A, Cardile D, Cucinotta M, Baldari S. Peptide receptor imaging in neuroendocrine tumors: comparison between diagnostic scintigraphy and post-therapy whole-body scan. *Ann Nucl Med* 2013;**27**(7):654-60.

385. Minutoli F, Laudicella R, Burger IA, Baldari S. Combined use of peptide receptor radionuclide therapy and metronomic chemotherapy in neuroendocrine tumors: a possible choice driven by nuclear medicine molecular imaging. *Eur J Nucl Med Mol Imaging* 2021;**48**(10):3041-2.

386. Mirzaei S, Bastati B, Lipp RW, Knoll P, Zojer N, Ludwig H. Additional lesions detected in therapeutic scans with 177Lu-DOTATATE reflect higher affinity of 177Lu-DOTATATE for somatostatin receptors. *Oncology* 2011;**80**(5-6):326-9.

387. Modlin IM, Moss SF, Oberg K, et al. Gastrointestinal neuroendocrine (carcinoid) tumours: current diagnosis and management. *Med J Aust* 2010;**193**(1):46-52.

388. Moghadam SZ, Divband G, Shakeri S, Aryana K. Favorable response after only one cycle of peptide receptor radionuclide therapy with 177Lu-DOTATATE in a patient with metastatic merkel cell carcinoma. *Clin Nucl Med* 2019;**44**(8):650-2.

389. Mogl MT, Dobrindt EM, Buschermöhle J, et al. Influence of gender on therapy and outcome of neuroendocrine tumors of gastroenteropancreatic origin: a single-center analysis. *Visc Med* 2020;**36**(1):20-7.

390. Montanier N, Joubert-Zakeyh J, Pétorin C, Montoriol PF, Maqdasy S, Kelly A. The prognostic influence of the proliferative discordance in metastatic pancreatic neuroendocrine carcinoma revealed by peptide receptor radionuclide therapy: case report and review of literature. *Medicine (Baltimore)* 2017;**96**(6):e6062.

391. Moorin RE, Meyrick DP, Rose A. Pre-clinical evaluation of 2,3-dimercaptosuccinic acid as a radiation nephrotoxicity protective agent during radiopeptide therapy of neuroendocrine malignancy. *Nucl Med Commun* 2007;**28**(4):261-6.

392. Mortensen J, Oturai P, Højgaard L, et al. Radionuclide therapy for neuroendocrine tumours [article in German]. *Ugeskr Laeger* 2010;**172**(43):2950-3.

393. Moyade P, Vinjamuri S. The heart matters: a review of incidental cardiac uptake on Ga-68 DOTA peptide PET-CT scans. *Nucl Med Commun* 2019;**40**(10):1081-5.

394. Mukherjee A, Lohar S, Dash A, Sarma HD, Samuel G, Korde A. Single vial kit formulation of DOTATATE for preparation of (177) Lu-labeled therapeutic radiopharmaceutical at hospital radiopharmacy. *J Labelled Comp Radiopharm* 2015;**58**(4):166-72.

395. Myers A, Chitwood H. Peptide receptor radionuclide therapy: an emerging treatment for gastrointestinal neuroendocrine tumors. *Clin J Oncol Nurs* 2020;**24**(2):129-33.

396. Nasir A, Gardner NM, Strosberg J, et al. Multimodality management of a polyfunctional pancreatic endocrine carcinoma with markedly elevated serum vasoactive intestinal polypeptide and calcitonin levels. *Pancreas* 2008;**36**(3):309-13.

397. Naswa N, Sharma P, Kumar A, et al. ^{68}Ga-DOTANOC PET/CT in patients with carcinoma of unknown primary of neuroendocrine origin. *Clin Nucl Med* 2012;**37**(3):245-51.

398. Navalkissoor S, Grossman A. Targeted alpha particle therapy for neuroendocrine tumours: the next generation of peptide receptor radionuclide therapy. *Neuroendocrinology* 2019;**108**(3):256-64.

399. Nelson KL, Sheetz MA. Radiation safety observations associated with 177Lu Dotatate patients. *Health Phys* 2019;**117**(6):680-7.

400. Neychev V, Kebebew E. Management options for advanced low or intermediate grade gastroenteropancreatic neuroendocrine tumors: review of recent literature. *Int J Surg Oncol* 2017;**2017**:6424812.

401. Nicolas GP, Mansi R, McDougall L, et al. Biodistribution, pharmacokinetics, and dosimetry of (177)Lu-, (90)Y-, and (111)in-labeled somatostatin receptor antagonist OPS201 in comparison to the agonist (177)Lu-DOTATATE: the mass effect. *J Nucl Med* 2017;**58**(9):1435-41.

402. Nicolini S, Bodei L, Bongiovanni A, et al. Combined use of 177Lu-DOTATATE and metronomic capecitabine (Lu-X) in FDG-positive gastro-entero-pancreatic neuroendocrine tumors. *Eur J Nucl Med Mol Imaging* 2021;**48**(10):3260-7.

403. Nicolini S, Severi S, Ianniello A, et al. Investigation of receptor radionuclide therapy with (177)Lu-DOTATATE in patients with GEP-NEN and a high Ki-67 proliferation index. *Eur J Nucl Med Mol Imaging* 2018;**45**(6):923-30.

404. Nilsson O, Kölby L, Bernhardt P, Forssell-Aronsson E, Johanson V, Ahlman H. GOT1 xenografted to nude mice: a unique model for in vivo studies on SSTR-mediated radiation therapy of carcinoid tumors. *Ann N Y Acad Sci* 2004;**1014**:275-9.

405. Nisa L, Savelli G, Giubbini R. Yttrium-90 DOTATOC therapy in GEP-NET and other SST2 expressing tumors: a selected review. *Ann Nucl Med* 2011;**25**(2):75-85.

406. Nonnekens J, van Kranenburg M, Beerens CE, et al. Potentiation of peptide receptor radionuclide therapy by the PARP inhibitor olaparib. *Theranostics* 2016;**6**(11):1821-32.

407. Novruzov F, Aliyev JA, Jaunmuktane Z, Bomanji JB, Kayani I. The use of (68)Ga DOTATATE PET/CT for diagnostic assessment and monitoring of (177)Lu DOTATATE therapy in pituitary carcinoma. *Clin Nucl Med* 2015;**40**(1):47-9.

408. O'Neill E, Kersemans V, Allen PD, et al. Imaging DNA damage repair in vivo after (177)Lu-DOTATATE therapy. *J Nucl Med* 2020;**61**(5):743-50.

409. Öberg K. Medical therapy of gastrointestinal neuroendocrine tumors. *Visc Med* 2017;**33**(5):352-6.

410. Oberg K, Eriksson B. Nuclear medicine in the detection, staging and treatment of gastrointestinal carcinoid tumours. *Best Pract Res Clin Endocrinol Metab* 2005;**19**(2):265-76.

411. Öberg KE. Gastrointestinal neuroendocrine tumors. *Ann Oncol* 2010;**21**(Suppl 7):vii72-80.

412. Oddstig J, Bernhardt P, Lizana H, et al. Inhomogeneous activity distribution of 177Lu-DOTA0-Tyr3-octreotate and effects on somatostatin receptor expression in human carcinoid GOT1 tumors in nude mice. *Tumour Biol* 2012;**33**(1):229-39.

413. Oh S, Prasad V, Lee DS, Baum RP. Effect of peptide receptor radionuclide therapy on somatostatin receptor status and glucose metabolism in neuroendocrine tumors: intraindividual comparison of Ga-68 DOTANOC PET/CT and F-18 FDG PET/CT. *Int J Mol Imaging* 2011;**2011**:524130.

414. Ohlendorf F, Henkenberens C, Brunkhorst T, et al. Volumetric 68Ga-DOTA-TATE PET/CT for assessment of whole-body tumor burden as a quantitative imaging biomarker in patients with metastatic gastroenteropancreatic neuroendocrine tumors. *Q J Nucl Med Mol Imaging* 2020; Apr 14. doi: 10.23736/S1824-4785.20.03238-0.

415. Ohlendorf F, Werner RA, Henkenberens C, et al. Predictive and prognostic impact of blood-based inflammatory biomarkers in patients with gastroenteropancreatic neuroendocrine tumors commencing peptide receptor radionuclide therapy. *Diagnostics (Basel)* 2021;**11**(3):504.

416. Öksüz M, Winter L, Pfannenberg C, et al. Peptide receptor radionuclide therapy of neuroendocrine tumors with (90)Y-DOTATOC: is treatment response predictable by pre-therapeutic uptake of (68)Ga-DOTATOC? *Diagn Interv Imaging* 2014;**95**(3):289-300.

417. Olmstead C, Cruz K, Stodilka R, Zabel P, Wolfson R. Quantifying public radiation exposure related to lutetium-177 octreotate therapy for the development of a safe outpatient treatment protocol. *Nucl Med Commun* 2015;**36**(2):129-34.

418. Önner H, Abdülrezzak Ü, Tutuş A. Could the skewness and kurtosis texture parameters of lesions obtained from pretreatment Ga-68 DOTA-TATE PET/CT images predict receptor radionuclide therapy response in patients with gastroenteropancreatic neuroendocrine tumors? *Nucl Med Commun* 2020;**41**(10):1034-9.

419. Ostwal V, Basu S, Bhargava P, et al. Capecitabine-temozolomide in advanced grade 2 and grade 3 neuroendocrine neoplasms: benefits of chemotherapy in neuroendocrine neoplasms with significant 18FDG uptake. *Neuroendocrinology* 2021;**111**(10):998-1004.

420. Oversoe SK, Sorensen BS, Tabaksblat EM, Gronbaek H, Kelsen J. Cell-free DNA and clinical characteristics in patients with small intestinal or pancreatic neuroendocrine tumors. *Neuroendocrinology* 2022;**112**(1):43-50..

421. Ozdemir NY, Arslan SA, Sendur MA. 177Lu-Dotatate for midgut neuroendocrine tumors. *N Engl J Med* 2017;**376**(14):1391.

422. Özdirik B, Amthauer H, Schatka I, et al. A rare case of a patient with a high grade neuroendocrine tumor developing neutropenic sepsis after receiving PRRT combined with capecitabine or temozolomide: a case report. *Mol Clin Oncol* 2021;**14**(1):20.

423. Özdirik B, Jann H, Bischoff P, et al. PD-L1 - inhibitors in neuroendocrine neoplasia: results from a real-life study. *Medicine (Baltimore)* 2021;**100**(1):e23835.

424. Paganelli G, Bodei L, Modlin I. A metastatic tumor is no different to a viral pandemic: lessons learnt from COVID-19 may teach us to change the PRRT paradigm. *Eur J Nucl Med Mol Imaging* 2020;**47**(10):2223-6.

425. Paganelli G, Sansovini M, Ambrosetti A, et al. 177 Lu-Dota-octreotate radionuclide therapy of advanced gastrointestinal neuroendocrine tumors: results from a phase II study. *Eur J Nucl Med Mol Imaging* 2014;**41**(10):1845-51.

426. Paganelli G, Sansovini M, Nicolini S, et al. (177)Lu-PRRT in advanced gastrointestinal neuroendocrine tumors: 10-year follow-up of the IRST phase II prospective study. *Eur J Nucl Med Mol Imaging* 2021;**48**(1):152-60.

427. Palmer J, Leeuwenkamp OR. Cost-effectiveness of lutetium ((177)Lu) oxodotreotide vs everolimus in gastroenteropancreatic neuroendocrine tumors in Norway and Sweden. *World J Clin Cases* 2020;**8**(20):4793-806.

428. Panigone S, Nunn AD. Lutetium-177-labeled gastrin releasing peptide receptor binding analogs: a novel approach to radionuclide therapy. *Q J Nucl Med Mol Imaging* 2006;**50**(4):310-21.

429. Panzuto F, Maccauro M, Campana D, et al. Impact of the SARS-CoV2 pandemic dissemination on the management of neuroendocrine neoplasia in Italy: a report from the Italian Association for Neuroendocrine Tumors (Itanet). *J Endocrinol Invest* 2021;**44**(5):989-94.

430. Panzuto F, Rinzivillo M, Spada F, et al. Everolimus in pancreatic neuroendocrine carcinomas G3. *Pancreas* 2017;**46**(3):302-5.

431. Papamichail DG, Exadaktylou PE, Chatzipavlidou VD. Neuroendocrine tumors: peptide receptors radionuclide therapy (PRRT) [article in Greek]. *Hell J Nucl Med* 2016;**19**(1):75-82.

432. Parghane RV, Basu S. Bilateral orbital soft-tissue metastases from renal neuroendocrine tumor: successful theranostic application of (68)Ga/(177)Lu-DOTATATE with improvement of vision. *J Nucl Med Technol* 2019;**47**(2):171-2.

433. Parghane RV, Basu S. Large cardiac metastasis from pancreatic neuroendocrine tumor and response to peptide receptor radionuclide therapy with (177)Lu-DOTATATE. *J Nucl Cardiol* 2020;**27**(1):340-1.

434. Parghane RV, Bhandare M, Chaudhari V, et al. Surgical feasibility, determinants and overall efficacy assessment of neoadjuvant PRRT with (177)Lu-DOTATATE for locally advanced unresectable gastroenteropancreatic neuroendocrine tumors. *J Nucl Med* 2021;**62**(11):1558-63.

435. Parghane RV, Mitra A, Bannore TU, Rakshit S, Banerjee S, Basu S. Initial clinical evaluation of indigenous (90)Y-DOTATATE in sequential duo-PRRT approach ((177)Lu-DOTATATE and (90)Y-DOTATATE) in neuroendocrine tumors with large bulky disease: observation on tolerability, (90)Y-DOTATATE post- PRRT imaging characteristics (bremsstrahlung and PETCT) and early adverse effects. *World J Nucl Med* 2021;**20**(1):73-81.

436. Parghane RV, Mitra A, Upadhye T, Rakshit S, Banerjee S, Basu S. Sequential duo-peptide receptor radionuclide therapy with indigenous 90Y-DOTATATE and 177Lu-DOTATATE in large-volume neuroendocrine tumors: posttherapy Bremsstrahlung and PET/CT imaging following 90Y-DOTATATE treatment. *Clin Nucl Med* 2020;**45**(9):714-5.

437. Parghane RV, Ostwal V, Ramaswamy A, et al. Long-term outcome of "Sandwich" chemo-PRRT: a novel treatment strategy for metastatic neuroendocrine tumors with both FDG- and SSTR-avid aggressive disease. *Eur J Nucl Med Mol Imaging* 2021;**48**(3):913-23.

438. Parghane RV, Talole S, Basu S. Prevalence of hitherto unknown brain meningioma detected on (68)Ga-DOTATATE positron-emission tomography/computed tomography in patients with metastatic neuroendocrine tumor and exploring potential of (177)Lu-DOTATATE peptide receptor radionuclide therapy as single-shot treatment approach targeting both tumors. *World J Nucl Med* 2019;**18**(2):160-70.

439. Parihar AS, Sood A, Sood A, Gulati A, Mittal BR. Demonstration of focal physiologic in-vivo somatostatin receptor expression in the caput epididymis of the testes on (68)Ga-DOTANOC PET/CT and (177)Lu-DOTATATE post-therapy whole body scintigraphy. *Asia Ocean J Nucl Med Biol* 2020;**8**(2):132-5.

440. Partelli S, Bertani E, Bartolomei M, et al. Peptide receptor radionuclide therapy as neoadjuvant therapy for resectable or potentially resectable pancreatic neuroendocrine neoplasms. *Surgery* 2018;**163**(4):761-7.

441. Patel D, Chan D, Cehic G, Pavlakis N, Price TJ. Systemic therapies for advanced gastroenteropancreatic neuroendocrine tumors. *Expert Rev Endocrinol Metab* 2016;**11**(4):311-27.

442. Pattison DA, Hofman MS. Role of fluorodeoxyglucose PET/computed tomography in targeted radionuclide therapy for endocrine malignancies. *PET Clin* 2015;**10**(4):461-76.

443. Pauwels E, Van Binnebeek S, Vandecaveye V, et al. Inflammation-based index and (68)Ga-DOTATOC PET-derived uptake and volumetric parameters predict outcome in neuroendocrine tumor patients treated with (90)Y-DOTATOC. *J Nucl Med* 2020;**61**(7):1014-20.

444. Pavel M. Metastatic neuroendocrine neoplasms. Non-surgical treatment strategies [article in German]. *Chirurg* 2011;**82**(7):612-7.

445. Pazaitou-Panayiotou K, Tiensuu Janson E, Koletsa T, et al. Somatostatin receptor expression in non-medullary thyroid carcinomas. *Hormones (Athens)* 2012;**11**(3):290-6.

446. Pellat A, Coriat R. Well differentiated grade 3 neuroendocrine tumors of the digestive tract: a narrative review. *J Clin Med* 2020;**9**(6):1677.

447. Pelle E, Al-Toubah T, Morse B, El-Haddad G, Strosberg J. Desmoplastic mesenteric lesions do not respond radiographically to peptide receptor radionuclide therapy. *J Neuroendocrinol* 2021;**33**(2):e12936.

448. Perri G, Prakash LR, Katz MHG. Pancreatic neuroendocrine tumors. *Curr Opin Gastroenterol* 2019;**35**(5):468-77.

449. Perysinakis I, Aggeli C, Kaltsas G, Zografos GN. Neoadjuvant therapy for advanced pancreatic neuroendocrine tumors: an emerging treatment modality? *Hormones (Athens)* 2016;**15**(1):15-22.

450. Petersen AL, Henriksen JR, Binderup T, et al. In vivo evaluation of PEGylated ^{64}Cu-liposomes with theranostic and radiotherapeutic potential using micro PET/CT. *Eur J Nucl Med Mol Imaging* 2016;**43**(5):941-52.

451. Pettersson O, Fröss-Baron K, Crona J, Sundin A. tumor contrast-enhancement for monitoring of PRRT (177)Lu-DOTATATE in pancreatic neuroendocrine tumor patients. *Front Oncol* 2020;**10**:193.

452. Pettersson OJ, Fröss-Baron K, Crona J, Sundin A. Tumor growth rate in pancreatic neuroendocrine tumor patients undergoing PRRT with 177Lu-DOTATATE. *Endocr Connect* 2021;**10**(4):422-31.

453. Plaza López PJ, Suarez Pinera M, Mestre Fusco A, Domenech Brasero B, Pifarré Muntané P, Rivera Codias E. Clinical impact of Ga(68)-DOTATOC PET/CT on neuroendocrine tumors. A preliminary experience. *Endocrinol Diabetes Nutr* 2020;**67**(10):636-42.

454. Poeppel TD, Binse I, Petersenn S, et al. 68Ga-DOTATOC versus 68Ga-DOTATATE PET/CT in functional imaging of neuroendocrine tumors. *J Nucl Med* 2011;**52**(12):1864-70.

455. Poeppel TD, Binse I, Petersenn S, et al. Differential uptake of (68)Ga-DOTATOC and (68)Ga-DOTATATE in PET/CT of gastroenteropancreatic neuroendocrine tumors. *Recent Results Cancer Res* 2013;**194**:353-71.

456. Poeppel TD, Boy C, Bockisch A, et al. Peptide receptor radionuclide therapy for patients with somatostatin receptor expressing tumours. German Guideline (S1) [article in German]. *Nuklearmedizin* 2015;**54**(1):1-11.

457. Pool SE, Kam BL, Koning GA, et al. [(111)In-DTPA]octreotide tumor uptake in GEPNET liver metastases after intra-arterial administration: an overview of preclinical and clinical observations and implications for tumor radiation dose after peptide radionuclide therapy. *Cancer Biother Radiopharm* 2014;**29**(4):179-87.

458. Pool SE, ten Hagen TL, Koelewijn S, de Jong M, Koning GA. Multimodality imaging of somatostatin receptor-positive tumors with nuclear and bioluminescence imaging. *Mol Imaging* 2012;**11**(1):27-32.

459. Pozas J, San Román M, Alonso-Gordoa T, et al. Targeting angiogenesis in pancreatic neuroendocrine tumors: resistance mechanisms. *Int J Mol Sci* 2019;**20**(19):4949.

460. Pozzari M, Maisonneuve P, Spada F, et al. Systemic therapies in patients with advanced well-differentiated pancreatic neuroendocrine tumors (PanNETs): When cytoreduction is the aim. A critical review with meta-analysis. *Cancer Treat Rev* 2018;**71**:39-46.

461. Prabhu M, Damle NA, Gupta R, Arora S, Arunraj ST, Bal C. Demonstration of 68Ga-prostate-specific membrane antigen uptake in metastatic pancreatic neuroendocrine tumor. *Indian J Nucl Med* 2018;**33**(3):257-8.

462. Prasad V, Ambrosini V, Alavi A, Fanti S, Baum RP. PET/CT in neuroendocrine tumors: evaluation of receptor status and metabolism. *PET Clin* 2007;**2**(3):351-75.

463. Prasad V, Ambrosini V, Alavi A, Fanti S, Baum RP. PET/CT in neuroendocrine tumors: evaluation of receptor status and metabolism. *PET Clin* 2008;**3**(3):355-79.

464. Prasad V, Srirajaskanthan R, Toumpanakis C, et al. Lessons from a multicentre retrospective study of peptide receptor radionuclide therapy combined with lanreotide for neuroendocrine tumours: a need for standardised practice. *Eur J Nucl Med Mol Imaging* 2020;**47**(10):2358-71.

465. Prosch H. Pulmonary carcinoid tumors [article in German]. *Radiologe* 2017;**57**(5):397-406.

466. Purohit NK, Shah RG, Adant S, Hoepfner M, Shah GM, Beauregard JM. Potentiation of (177)Lu-octreotate peptide receptor radionuclide therapy of human neuroendocrine tumor cells by PARP inhibitor. *Oncotarget* 2018;**9**(37):24693-706.

467. Pusceddu S, Buzzoni R, de Braud F. The underestimated role of somatostatin analogs in the NETTER-1 trial. *Future Oncol* 2017;**13**(15):1287-9.

468. Pusceddu S, De Braud F, Festinese F, et al. Evolution in the treatment of gastroenteropancreatic-neuroendocrine neoplasms, focus on systemic therapeutic options: a systematic review. *Future Oncol* 2015;**11**(13):1947-59.

469. Pusceddu S, Femia D, Lo Russo G, et al. Update on medical treatment of small intestinal neuroendocrine tumors. *Expert Rev Anticancer Ther* 2016;**16**(9):969-76.

470. Puszkiel A, Bauriaud-Mallet M, Bourgeois R, Dierickx L, Courbon F, Chatelut E. Evaluation of the interaction of amino acid infusion on (177)Lu-Dotatate pharmacokinetics in patients with gastroenteropancreatic neuroendocrine tumors. *Clin Pharmacokinet* 2019;**58**(2):213-22.

471. Putzer D, Kroiss A, Waitz D, et al. Somatostatin receptor PET in neuroendocrine tumours: 68Ga-DOTA0,Tyr3-octreotide versus 68Ga-DOTA0-lanreotide. *Eur J Nucl Med Mol Imaging* 2013;**40**(3):364-72.

472. Raheem SJ, Schmidt BW, Solomon VR, Salih AK, Price EW. Ultrasonic-assisted solid-phase peptide synthesis of DOTA-TATE and DOTA-linker-TATE derivatives as a simple and low-cost method for the facile synthesis of chelator-peptide conjugates. *Bioconjug Chem* 2021;**32**(7):1204-13.

473. Ramage J, Naraev BG, Halfdanarson TR. Peptide receptor radionuclide therapy for patients with advanced pancreatic neuroendocrine tumors. *Semin Oncol* 2018;**45**(4):236-48.

474. Ramesh S, Kudachi S, Basu S. Peptide receptor radionuclide therapy with (177)Lu-DOTATATE in carcinoid heart disease: a contraindication or a promising treatment approach bettering chances for corrective surgery? *J Nucl Med Technol* 2018;**46**(3):292-4.

475. Ranade R, Basu S. Metabolic bone disease in the context of metastatic neuroendocrine tumor: differentiation from skeletal metastasis, the molecular PET-CT imaging features, and exploring the possible etiopathologies including parathyroid adenoma (MEN1) and paraneoplastic humoral hypercalcemia of malignancy due to PTHrP hypersecretion. *World J Nucl Med* 2017;**16**(1):62-7.

476. Raymond LM, Korzun T, Kardosh A, Kolbeck KJ, Pommier RF, Mittra ES. The state of PRRT and its sequencing amongst current therapeutic options for gastroenteropancreatic neuroendocrine tumors. *Neuroendocrinology* 2021;**111**(11):1086-98.

477. Reed NS. Neuroendocrine tumours of the gynecological tract. *Curr Opin Oncol* 2016;**28**(5):412-8.

478. Refardt J, Zandee WT, Brabander T, et al. Inferior outcome of neuroendocrine tumor patients negative on somatostatin receptor imaging. *Endocr Relat Cancer* 2020;**27**(11):615-24.

479. Reidy-Lagunes D, Pandit-Taskar N, O'Donoghue JA, et al. Phase I trial of well-differentiated neuroendocrine tumors (NETs) with radiolabeled somatostatin antagonist (177)Lu-satoreotide tetraxetan. *Clin Cancer Res* 2019;**25**(23):6939-47.

480. Reismann P, Kender Z, Dabasi G, Sréter L, Rácz K, Igaz P. Somatostatin receptor endoradiotherapy of neuroendocrine tumors: experience in Hungarian patients [article in Hungarian]. *Orv Hetil* 2011;**152**(10):392-7.

481. Riff BP, Yang YX, Soulen MC, et al. Peptide receptor radionuclide therapy-induced hepatotoxicity in patients with metastatic neuroendocrine tumors. *Clin Nucl Med* 2015;**40**(11):845-50.

482. Rindi G, Wiedenmann B. Neuroendocrine neoplasia of the gastrointestinal tract revisited: towards precision medicine. *Nat Rev Endocrinol* 2020;**16**(10):590-607.

483. Ritler A, Shoshan MS, Deupi X, et al. Elucidating the structure-activity relationship of the pentaglutamic acid sequence of minigastrin with cholecystokinin receptor subtype 2. *Bioconjug Chem* 2019;**30**(3):657-66.

484. Rizvi SFA, Naqvi SAR, Roohi S, Sherazi TA, Rasheed R. (177)Lu-DOTA-coupled minigastrin peptides: promising theranostic agents in neuroendocrine cancers. *Mol Biol Rep* 2018;**45**(6):1759-67.

485. Rodrigues M, Winkler KK, Svirydenka H, Nilica B, Uprimny C, Virgolini I. Long-term survival and value of (18)F-FDG PET/CT in patients with gastroenteropancreatic neuroendocrine tumors treated with second peptide receptor radionuclide therapy course with (177)Lu-DOTATATE. *Life (Basel)* 2021;**11**(3):198.

486. Rogowski W, Wachuła E, Lewczuk A, et al. Baseline chromogranin A and its dynamics are prognostic markers in gastroenteropancreatic neuroendocrine tumors. *Future Oncol* 2017;**13**(12):1069-79.

487. Rolleman EJ, Valkema R, de Jong M, Kooij PP, Krenning EP. Safe and effective inhibition of renal uptake of radiolabelled octreotide by a combination of lysine and arginine. *Eur J Nucl Med Mol Imaging* 2003;**30**(1):9-15.

488. Roohi S, Rizvi SK, Naqvi SAR. (177)Lu-DOTATATE peptide receptor radionuclide therapy: indigenously developed freeze dried cold kit and biological response in in-vitro and in-vivo models. *Dose Response* 2021;**19**(1):1559325821990147.

489. Rossi RE, Massironi S, Spampatti MP, et al. Treatment of liver metastases in patients with digestive neuroendocrine tumors. *J Gastrointest Surg* 2012;**16**(10):1981-92.

490. Rottenburger C, Nicolas GP, McDougall L, et al. Cholecystokinin 2 receptor agonist (177)Lu-PP-F11N for radionuclide therapy of medullary thyroid carcinoma: results of the lumed phase 0a study. *J Nucl Med* 2020;**61**(4):520-6.

491. Rousseau E, Lau J, Zhang Z, et al. Effects of adding an albumin binder chain on [(177)Lu]Lu-DOTATATE. *Nucl Med Biol* 2018;**66**:10-7.

492. Rufini V, Baum RP, Castaldi P, et al. Role of PET/CT in the functional imaging of endocrine pancreatic tumors. *Abdom Imaging* 2012;**37**(6):1004-20.

493. Ryan J, Akhurst T, Lynch AC, Michael M, Heriot AG. Neoadjuvant (90) Yttrium peptide receptor radionuclide therapy for advanced rectal neuroendocrine tumour: a case report. *ANZ J Surg* 2017;**87**(1-2):92-3.

494. Sabet A, Dautzenberg K, Haslerud T, et al. Specific efficacy of peptide receptor radionuclide therapy with (177)Lu-octreotate in advanced neuroendocrine tumours of the small intestine. *Eur J Nucl Med Mol Imaging* 2015;**42**(8):1238-46.

495. Sabet A, Ezziddin K, Pape UF, et al. Accurate assessment of long-term nephrotoxicity after peptide receptor radionuclide therapy with (177)Lu-octreotate. *Eur J Nucl Med Mol Imaging* 2014;**41**(3):505-10.

496. Sabet A, Ezziddin S, Heinemann F, et al. Osseous metastases of gastro-enteropancreatic neuroendocrine tumours. Diagnostic value of intra-therapeutic 177Lu-octreotate imaging in comparison with bone scintigraphy. *Nuklearmedizin* 2012;**51**(3):95-100.

497. Sabet A, Haslerud T, Pape UF, et al. Outcome and toxicity of salvage therapy with 177Lu-octreotate in patients with metastatic gastroenteropancreatic neuroendocrine tumours. *Eur J Nucl Med Mol Imaging* 2014;**41**(2):205-10.

498. Sabet A, Khalaf F, Haslerud T, et al. Bone metastases in GEP-NET: response and long-term outcome after PRRT from a follow-up analysis. *Am J Nucl Med Mol Imaging* 2013;**3**(5):437-45.

499. Sabet A, Khalaf F, Mahjoob S, Al-Zreiqat A, Biersack HJ, Ezziddin S. May bone-targeted radionuclide therapy overcome PRRT-refractory osseous disease in NET? A pilot report on (188)Re-HEDP treatment in progressive bone metastases after (177)Lu-octreotate. *Am J Nucl Med Mol Imaging* 2013;**4**(1):80-8.

500. Sabet A, Khalaf F, Yong-Hing CJ, et al. Can peptide receptor radionuclide therapy be safely applied in florid bone metastases? A pilot analysis of late stage osseous involvement. *Nuklearmedizin* 2014;**53**(2):54-9.

501. Sabet A, Nagarajah J, Dogan AS, et al. Does PRRT with standard activities of 177Lu-octreotate really achieve relevant somatostatin receptor saturation in target tumor lesions?: insights from intra-therapeutic receptor imaging in patients with metastatic gastroenteropancreatic neuroendocrine tumors. *EJNMMI Res* 2013;**3**(1):82.

502. Sabongi JG, Gonçalves MC, Alves CD, Alves J, Scapulatempo-Neto C, Moriguchi SM. Lutetium 177-DOTA-TATE therapy for esthesioneuroblastoma: a case report. *Exp Ther Med* 2016;**12**(5):3078-82.

503. Sainz-Esteban A, Prasad V, Schuchardt C, Zachert C, Carril JM, Baum RP. Comparison of sequential planar 177Lu-DOTA-TATE dosimetry scans with 68Ga-DOTA-TATE PET/CT images in patients with metastasized neuroendocrine tumours undergoing peptide receptor radionuclide therapy. *Eur J Nucl Med Mol Imaging* 2012;**39**(3):501-11.

504. Salavati A, Prasad V, Schneider CP, Herbst R, Baum RP. Peptide receptor radionuclide therapy of Merkel cell carcinoma using (177)lutetium-labeled somatostatin analogs in combination with radiosensitizing chemotherapy: a potential novel treatment based on molecular pathology. *Ann Nucl Med* 2012;**26**(4):365-9.

505. Sampaio IL, Luiz HV, Violante LS, et al. Treatment of gastroenteropancreatic neuroendocrine tumors with 177Lu-DOTA-TATE: experience of the Portuguese Institute of Oncology in Porto [article in Portuguese]. *Acta Med Port* 2016;**29**(11):726-33.

506. Sampaio IL, Luíz HV, Violante LS, et al. [Errata to the article "Treatment of Gastroenteropancreatic Neuroendocrine Tumors with 177Lu-DOTA-TATE: Experience of the Portuguese Institute of Oncology in Porto" by Inês Lucena Sampaio, Henrique Vara Luiz, Liliana Sobral Violante, Ana Paula Santos, Luís Antunes, Isabel Torres, Cristina Sanches, Isabel Azevedo, Hugo Duarte published on Acta Med Port 2016;**29**(11):726-733]. *Acta Med Port* 2020;**33**(1):81-2.

507. Sampathirao N, Basu S. MIB-1 Index-stratified assessment of dual-tracer PET/CT with (68)Ga-DOTATATE and (18)F-FDG and multimodality anatomic imaging in metastatic neuroendocrine tumors of unknown primary in a PRRT workup setting. *J Nucl Med Technol* 2017;**45**(1):34-41.

508. Sampathirao N, Indirani M, Shelley S. Primary pancreatic lymphoma camouflaged under the umbrella of spectrum of neuroendocrine tumors in somatostatin receptor imaging. *World J Nucl Med* 2020;**19**(3):306-9.

509. Sanders JC, Kuwert T, Hornegger J, Ritt P. Quantitative SPECT/CT imaging of (177)Lu with in vivo validation in patients undergoing peptide receptor radionuclide therapy. *Mol Imaging Biol* 2015;**17**(4):585-93.

510. Sandström M, Freedman N, Fröss-Baron K, Kahn T, Sundin A. Kidney dosimetry in 777 patients during (177)Lu-DOTATATE therapy: aspects on extrapolations and measurement time points. *EJNMMI Phys* 2020;**7**(1):73.

511. Sandström M, Garske-Román U, Johansson S, Granberg D, Sundin A, Freedman N. Kidney dosimetry during (177)Lu-DOTATATE therapy in patients with neuroendocrine tumors: aspects on calculation and tolerance. *Acta Oncol* 2018;**57**(4):516-21.

512. Sanli Y, Garg I, Kandathil A, et al. Neuroendocrine tumor diagnosis and management: (68)Ga-DOTATATE PET/CT. *AJR Am J Roentgenol* 2018;**211**(2):267-77.

513. Sansovini M, Severi S, Ambrosetti A, et al. Treatment with the radiolabelled somatostatin analog Lu-DOTATATE for advanced pancreatic neuroendocrine tumors. *Neuroendocrinology* 2013;**97**(4):347-54.

514. Sansovini M, Severi S, Ianniello A, et al. Long-term follow-up and role of FDG PET in advanced pancreatic neuro-endocrine patients treated with (177)Lu-D OTATATE. *Eur J Nucl Med Mol Imaging* 2017;**44**(3):490-9.

515. Santoro L, Mora-Ramirez E, Trauchessec D, et al. Implementation of patient dosimetry in the clinical practice after targeted radiotherapy using [(177)Lu-[DOTA0, Tyr3]-octreotate. *EJNMMI Res* 2018;**8**(1):103.

516. Saponjski J, Macut D, Sobic-Saranovic D, et al. Somatostatin receptor scintigraphy in the follow up of neuroendocrine neoplasms of appendix. *World J Clin Cases* 2020;**8**(17):3697-707.

517. Satapathy S, Mittal BR. 177Lu-DOTATATE peptide receptor radionuclide therapy versus Everolimus in advanced pancreatic neuroendocrine tumors: a systematic review and meta-analysis. *Nucl Med Commun* 2019;**40**(12):1195-203.

518. Satapathy S, Mittal BR, Sood A, Verma R, Panda N. Efficacy and safety of concomitant 177Lu-DOTATATE and low-dose capecitabine in advanced medullary thyroid carcinoma: a single-centre experience. *Nucl Med Commun* 2020;**41**(7):629-35.

519. Sauter AW, Mansi R, Hassiepen U, et al. Targeting of the cholecystokinin-2 receptor with the minigastrin analog (177)Lu-DOTA-PP-F11N: does the use of protease inhibitors further improve in vivo distribution? *J Nucl Med* 2019;**60**(3):393-9.

520. Savelli G, Bertagna F, Franco F, et al. Final results of a phase 2A study for the treatment of metastatic neuroendocrine tumors with a fixed activity of 90Y-DOTA-D-Phe1-Tyr3 octreotide. *Cancer* 2012;**118**(11):2915-24.

521. Savolainen S, Konijnenberg M, Bardiès M, et al. Radiation dosimetry is a necessary ingredient for a perfectly mixed molecular radiotherapy cocktail. *Eur J Nucl Med Mol Imaging* 2012;**39**(3):548-9.

522. Savovic T, Prior JO, Nicod-Lalonde M, et al. First experience of durable cytoreduction in chronic lymphoid leukemia with (177)Lu-DOTATATE. *Med Oncol* 2019;**36**(5):41.

523. Sayeg Y, Sayeg M, Baum RP, et al. Pulmonary neuroendocrine neoplasms [article in German]. *Pneumologie* 2014;**68**(7):456-77.

524. Scheidhauer K, Miederer M, Gaertner FC. PET-CT for neuroendocrine tumors and nuclear medicine therapy options [article in German]. *Radiologe* 2009;**49**(3):217-23.

525. Schiavo Lena M, Partelli S, Castelli P, et al. Histopathological and immunophenotypic changes of pancreatic neuroendocrine tumors after neoadjuvant peptide receptor radionuclide therapy (PRRT). *Endocr Pathol* 2020;**31**(2):119-31.

526. Schmidt M, Baum RP, Simon T, Howman-Giles R. Therapeutic nuclear medicine in pediatric malignancy. *Q J Nucl Med Mol Imaging* 2010;**54**(4):411-28.

527. Schmidt MC, Uhrhan K, Fischer T, et al. Complete remission of metastatic neuroendocrine paragastric carcinoma after "neoadjuvant" peptide receptor radionuclide therapy and surgery. *Clin Nucl Med* 2015;**40**(8):667-9.

528. Schmitt A, Bernhardt P, Nilsson O, et al. Radiation therapy of small cell lung cancer with 177Lu-DOTA-Tyr3-octreotate in an animal model. *J Nucl Med* 2004;**45**(9):1542-8.

529. Schneider JR, Shatzkes DR, Scharf SC, et al. Neuroradiological and neuropathological changes after 177Lu-octreotate peptide receptor radionuclide therapy of refractory esthesioneuroblastoma. *Oper Neurosurg (Hagerstown)* 2018;**15**(6):100-9.

530. Schuchardt C, Kulkarni HR, Prasad V, Zachert C, Müller D, Baum RP. The Bad Berka dose protocol: comparative results of dosimetry in peptide receptor radionuclide therapy using (177)Lu-DOTATATE, (177)Lu-DOTANOC, and (177)Lu-DOTATOC. *Recent Results Cancer Res* 2013;**194**:519-36.

531. Scott AT, Howe JR. Management of small bowel neuroendocrine tumors. *Surg Oncol Clin N Am* 2020;**29**(2):223-41.

532. Scoville SD, Cloyd JM, Pawlik TM. New and emerging systemic therapy options for well-differentiated gastroenteropancreatic neuroendocrine tumors. *Expert Opin Pharmacother* 2020;**21**(2):183-91.

533. Segelov E, Chan D, Lawrence B, et al. Identifying and prioritizing gaps in neuroendocrine tumor research: a modified delphi process with patients and health care providers to set the research action plan for the newly formed commonwealth neuroendocrine tumor collaboration. *J Glob Oncol* 2017;**3**(4):380-8.

534. Seifert V, Richter S, Bechmann N, et al. HIF2alpha-associated pseudohypoxia promotes radioresistance in pheochromocytoma: insights from 3D models. *Cancers (Basel)* 2021;**13**(3):385.

535. Seregni E, Maccauro M, Chiesa C, et al. Treatment with tandem [90Y]DOTA-TATE and [177Lu]DOTA-TATE of neuroendocrine tumours refractory to conventional therapy. *Eur J Nucl Med Mol Imaging* 2014;**41**(2):223-30.

536. Seregni E, Maccauro M, Coliva A, et al. Treatment with tandem [(90)Y]DOTA-TATE and [(177)Lu] DOTA-TATE of neuroendocrine tumors refractory to conventional therapy: preliminary results. *Q J Nucl Med Mol Imaging* 2010;**54**(1):84-91.

537. Severi S, Grassi I, Nicolini S, Sansovini M, Bongiovanni A, Paganelli G. Peptide receptor radionuclide therapy in the management of gastrointestinal neuroendocrine tumors: efficacy profile, safety, and quality of life. *Onco Targets Ther* 2017;**10**:551-7.

538. Severi S, Sansovini M, Ianniello A, et al. Feasibility and utility of re-treatment with (177)Lu-DOTATATE in GEP-NENs relapsed after treatment with (90)Y-DOTATOC. *Eur J Nucl Med Mol Imaging* 2015;**42**(13):1955-63.

539. Shah M, Da Silva R, Gravekamp C, Libutti SK, Abraham T, Dadachova E. Targeted radionuclide therapies for pancreatic cancer. *Cancer Gene Ther* 2015;**22**(8):375-9.

540. Shah RG, Merlin MA, Adant S, Zine-Eddine F, Beauregard JM, Shah GM. Chemotherapy-induced upregulation of somatostatin receptor-2 increases the uptake and efficacy of (177)Lu-DOTA-octreotate in neuroendocrine tumor cells. *Cancers (Basel)* 2021;**13**(2):232.

541. Sharma P, Basu S. Occurrence of the redifferentiation-akin phenomenon on (68)Ga-DOTATATE PET/CT after CAPTEM chemotherapy in metastatic neuroendocrine tumors with intermediate MIB1 Index: what could be the molecular explanation? *J Nucl Med Technol* 2020;**48**(3):290-1.

542. Shimata K, Sugawara Y, Hibi T. Liver transplantation for unresectable pancreatic neuroendocrine tumors with liver metastases in an era of transplant oncology. *Gland Surg* 2018;**7**(1):42-6.

543. Sindoni A, Famà F, Gioffre-Florio M, Baldari S. Emerging treatment modalities for advanced pancreatic neuroendocrine tumors. *Hormones (Athens)* 2016;**15**(4):563-4.

544. Singh A, van der Meulen NP, Müller C, et al. First-in-human PET/CT imaging of metastatic neuroendocrine neoplasms with cyclotron-produced (44)Sc-DOTATOC: a proof-of-concept study. *Cancer Biother Radiopharm* 2017;**32**(4):124-32.

545. Singh S, Asa SL, Dey C, et al. Diagnosis and management of gastrointestinal neuroendocrine tumors: an evidence-based Canadian consensus. *Cancer Treat Rev* 2016;**47**:32-45.

546. Singh S, Dey C, Kennecke H, et al. Consensus recommendations for the diagnosis and management of pancreatic neuroendocrine tumors: guidelines from a Canadian National Expert Group. *Ann Surg Oncol* 2015;**22**(8):2685-99.

547. Singh S, Poon R, Wong R, Metser U. 68Ga PET imaging in patients with neuroendocrine tumors: a systematic review and meta-analysis. *Clin Nucl Med* 2018;**43**(11):802-10.

548. Singla S, Gupta S, Reddy RM, Durgapal P, Bal CS. 68Ga-DOTA-NOC PET and peptide receptor radionuclide therapy in management of bilateral ovarian metastases from gastrointestinal carcinoid. *Jpn J Clin Oncol* 2012;**42**(12):1202-6.

549. Sipka G, Besenyi Z, Farkas I, Pávics L. Theranostics in 2020: Neuroendocrine tumors [article in Hungarian]. *Magy Onkol* 2020;**64**(2):119-30.

550. Slooter GD, Aalbers AG, Breeman WA, et al. The inhibitory effect of (111)In-DTPA(0)-octreotide on intrahepatic tumor growth after partial hepatectomy. *J Nucl Med* 2002;**43**(12):1681-7.

551. Slooter GD, Breeman WA, Marquet RL, Krenning EP, van Eijck CH. Anti-proliferative effect of radiolabelled octreotide in a metastases model in rat liver. *Int J Cancer* 1999;**81**(5):767-71.

552. Smith-Palmer J, Leeuwenkamp OR, Virk J, Reed N. Lutetium oxodotreotide ((177)Lu-Dotatate) for the treatment of unresectable or metastatic progressive gastroenteropancreatic neuroendocrine tumors: a cost-effectiveness analysis for Scotland. *BMC Cancer* 2021;**21**(1):10.

553. Soeiro P, Martins H, Silva R, Moreira AP. Complete remission of inoperable hepatic and bone metastases due to neuroendocrine pancreatic tumour 3 years after peptide receptor radionucleotide therapy. *BMJ Case Rep* 2020;**13**(3):e233263.

554. Sorbye H, Kong G, Grozinsky-Glasberg S. PRRT in high-grade gastroenteropancreatic neuroendocrine neoplasms (WHO G3). *Endocr Relat Cancer* 2020;**27**(3):R67-r77.

555. Sowa-Staszczak A, Pach D, Kunikowska J, et al. Efficacy and safety of 90Y-DOTATATE therapy in neuroendocrine tumours. *Endokrynol Pol* 2011;**62**(5):392-400.

556. Sowa-Staszczak A, Pach D, Stefańska A, et al. Case report of a patient with initially inoperable well-differentiated midgut neuroendocrine tumor (WDNT)--PRRT and long-acting somatostatin analogs as the neoadjuvant therapy. *Nucl Med Rev Cent East Eur* 2012;**15**(2):137-9.

557. Sowa-Staszczak A, Pach D, Stefańska A, et al. Can treatment using radiolabelled somatostatin analogue increase the survival rate in patients with non-functioning neuroendocrine pancreatic tumours? *Nucl Med Rev Cent East Eur* 2011;**14**(2):73-8.

558. Spetz J, Langen B, Rudqvist N, et al. Hedgehog inhibitor sonidegib potentiates (177)Lu-octreotate therapy of GOT1 human small intestine neuroendocrine tumors in nude mice. *BMC Cancer* 2017;**17**(1):528.

559. Spetz J, Langen B, Rudqvist NP, et al. Transcriptional effects of (177)Lu-octreotate therapy using a priming treatment schedule on GOT1 tumor in nude mice. *EJNMMI Res* 2019;**9**(1):28.

560. Spetz J, Rudqvist N, Langen B, et al. Time-dependent transcriptional response of GOT1 human small intestine neuroendocrine tumor after (177)Lu[Lu]-octreotate therapy. *Nucl Med Biol* 2018;**60**:11-8.

561. Srirajaskanthan R, Toumpanakis C, Karpathakis A, et al. Surgical management and palliative treatment in bronchial neuroendocrine tumours: a clinical study of 45 patients. *Lung Cancer* 2009;**65**(1):68-73.

562. Stahl A, Meisetschläger G, Schottelius M, et al. [123I]Mtr-TOCA, a radioiodinated and carbohydrated analogue of octreotide: scintigraphic comparison with [111In]octreotide. *Eur J Nucl Med Mol Imaging* 2006;**33**(1):45-52.

563. Stallons TAR, Saidi A, Tworowska I, Delpassand ES, Torgue JJ. Preclinical investigation of (212)Pb-DOTAMTATE for peptide receptor radionuclide therapy in a neuroendocrine tumor model. *Mol Cancer Ther* 2019;**18**(5):1012-21.

564. Starr JS, Sonbol MB, Hobday TJ, Sharma A, Kendi AT, Halfdanarson TR. Peptide receptor radionuclide therapy for the treatment of pancreatic neuroendocrine tumors: recent insights. *Onco Targets Ther* 2020;**13**:3545-55.

565. Strosberg J. (177)Lutetium-Dotatate delays decline in quality of life in patients with midgut neuroendocrine tumors. *Oncotarget* 2018;**9**(69):33059.

566. Strosberg J, El-Haddad G, Wolin E, et al. Phase 3 trial of (177)Lu-Dotatate for midgut neuroendocrine tumors. *N Engl J Med* 2017;**376**(2):125-35.

567. Strosberg J, Krenning E. 177Lu-Dotatate for midgut neuroendocrine tumors. *N Engl J Med* 2017;**376**(14):1391-2.

568. Strosberg J, Kunz PL, Hendifar A, et al. Impact of liver tumour burden, alkaline phosphatase elevation, and target lesion size on treatment outcomes with (177)Lu-Dotatate: an analysis of the NETTER-1 study. *Eur J Nucl Med Mol Imaging* 2020;**47**(10):2372-82.

569. Strosberg J, Wolin E, Chasen B, et al. Health-related quality of life in patients with progressive midgut neuroendocrine tumors treated with (177)Lu-Dotatate in the phase III NETTER-1 trial. *J Clin Oncol* 2018;**36**(25):2578-84.

570. Strosberg JR, Srirajaskanthan R, El-Haddad G, et al. Symptom diaries of patients with midgut neuroendocrine tumors treated with (177)Lu-DOTATATE. *J Nucl Med* 2021;**62**(12):1712-8.

571. Svensson J, Mölne J, Forssell-Aronsson E, Konijnenberg M, Bernhardt P. Nephrotoxicity profiles and threshold dose values for [177Lu]-DOTATATE in nude mice. *Nucl Med Biol* 2012;**39**(6):756-62.

572. Swärd C, Bernhardt P, Ahlman H, et al. [177Lu-DOTA 0-Tyr 3]-octreotate treatment in patients with disseminated gastroenteropancreatic neuroendocrine tumors: the value of measuring absorbed dose to the kidney. *World J Surg* 2010;**34**(6):1368-72.

573. Swärd C, Bernhardt P, Johanson V, et al. Comparison of [177Lu-DOTA0,Tyr3]-octreotate and [177Lu-DOTA0,Tyr3]-octreotide for receptor-mediated radiation therapy of the xenografted human midgut carcinoid tumor GOT1. *Cancer Biother Radiopharm* 2008;**23**(1):114-20.

574. Taïeb D, Garrigue P, Bardiès M, Abdullah AE, Pacak K. Application and dosimetric requirements for gallium-68-labeled somatostatin analogues in targeted radionuclide therapy for gastroenteropancreatic neuroendocrine tumors. *PET Clin* 2015;**10**(4):477-86.

575. Taïeb D, Hicks RJ, Hindié E, et al. European Association of Nuclear Medicine Practice Guideline/Society of Nuclear Medicine and Molecular Imaging Procedure Standard 2019 for radionuclide imaging of phaeochromocytoma and paraganglioma. *Eur J Nucl Med Mol Imaging* 2019;**46**(10):2112-37.

576. Teunissen JJ, Kwekkeboom DJ, de Jong M, Esser JP, Valkema R, Krenning EP. Endocrine tumours of the gastrointestinal tract. Peptide receptor radionuclide therapy. *Best Pract Res Clin Gastroenterol* 2005;**19**(4):595-616.

577. Teunissen JJ, Kwekkeboom DJ, Krenning EP. Quality of life in patients with gastroenteropancreatic tumors treated with [177Lu-DOTA0,Tyr3]octreotate. *J Clin Oncol* 2004;**22**(13):2724-9.

578. Thakral P, Sen I, Pant V, et al. Dosimetric analysis of patients with gastro entero pancreatic neuroendocrine tumors (NETs) treated with PRCRT (peptide receptor chemo radionuclide therapy) using Lu-177 DOTATATE and capecitabine/temozolomide (CAP/TEM). *Br J Radiol* 2018;**91**(1091):20170172.

579. Thapa P, Ranade R, Ostwal V, Shrikhande SV, Goel M, Basu S. Performance of 177Lu-DOTATATE-based peptide receptor radionuclide therapy in metastatic gastroenteropancreatic neuroendocrine tumor: a multiparametric response evaluation correlating with primary tumor site, tumor proliferation index, and dual tracer imaging characteristics. *Nucl Med Commun* 2016;**37**(10):1030-7.

580. The MRT. (111)Indium-diethylenetriaminepentaacetic acid-d-phenylalanine-octreotide. Molecular Imaging and Contrast Agent Database (MICAD). Bethesda (MD): National Center for Biotechnology Information (US); 2004.

581. Thisgaard H, Olsen BB, Dam JH, Bollen P, Mollenhauer J, Høilund-Carlsen PF. Evaluation of cobalt-labeled octreotide analogs for molecular imaging and auger electron-based radionuclide therapy. *J Nucl Med* 2014;**55**(8):1311-6.

582. Thomas KEH, Voros BA, Boudreaux JP, Thiagarajan R, Woltering EA, Ramirez RA. Current treatment options in gastroenteropancreatic neuroendocrine carcinoma. *Oncologist* 2019;**24**(8):1076-88.

583. Thuillier P, Maajem M, Schick U, et al. Clinical assessment of 177Lu-DOTATATE quantification by comparison of SUV-based parameters measured on both post-PRRT SPECT/CT and 68Ga-DOTATOC PET/CT in patients with neuroendocrine tumors: a feasibility study. *Clin Nucl Med* 2021;**46**(2):111-8.

584. Titan AL, Norton JA, Fisher AT, et al. Evaluation of outcomes following surgery for locally advanced pancreatic neuroendocrine tumors. *JAMA Netw Open* 2020;**3**(11):e2024318.

585. Todorović-Tirnanić M, Kaemmerer D, Prasad V, Hommann M, Baum RP. Intraoperative somatostatin receptor detection after peptide receptor radionuclide therapy with (177)Lu- and (90)Y-DOTATOC (tandem PRRNT) in a patient with a metastatic neuroendocrine tumor. *Recent Results Cancer Res* 2013;**194**:487-96.

586. Tolomeo A, Lopopolo G, Dimiccoli V, Perioli L, Modoni S, Scilimati A. Impact of 68Ga-DOTATOC PET/CT in comparison to 111In-Octreotide SPECT/CT in management of neuro-endocrine tumors: a case report. *Medicine (Baltimore)* 2020;**99**(7):e19162.

587. Toumpanakis C, Caplin ME. Update on the role of somatostatin analogs for the treatment of patients with gastroenteropancreatic neuroendocrine tumors. *Semin Oncol* 2013;**40**(1):56-68.

588. Tran CG, Sherman SK, Howe JR. The Landmark Series: management of small bowel neuroendocrine tumors. *Ann Surg Oncol* 2021;**28**(5):2741-51.

589. Tran K, Khan S, Taghizadehasl M, et al. Gallium-68 Dotatate PET/CT is superior to other imaging modalities in the detection of medullary carcinoma of the thyroid in the presence of high serum calcitonin. *Hell J Nucl Med* 2015;**18**(1):19-24.

590. Treglia G, Kroiss AS, Piccardo A, Lococo F, Santhanam P, Imperiale A. Role of positron emission tomography in thyroid and neuroendocrine tumors. *Minerva Endocrinol* 2018;**43**(3):341-55.

591. Turaga KK, Kvols LK. Recent progress in the understanding, diagnosis, and treatment of gastroenteropancreatic neuroendocrine tumors. *CA Cancer J Clin* 2011;**61**(2):113-32.

592. Uhlyarik A, Lahm E, Vachaja J, Pápai Z. Treatment of metastatic midgut neuroendocrine tumour associated with carcinoid syndrome [article in Hungarian]. *Orv Hetil* 2014;**155**(5):194-8.

593. Underwood J, Sturchio G, Arnold S. Patient release and instructions for lutetium dotatate radiopharmaceutical therapy. *Health Phys* 2021;**121**(2):160-5.

594. Uri I, Grozinsky-Glasberg S. Current treatment strategies for patients with advanced gastroenteropancreatic neuroendocrine tumors (GEP-NETs). *Clin Diabetes Endocrinol* 2018;**4**:16.

595. Valkema R, De Jong M, Bakker WH, et al. Phase I study of peptide receptor radionuclide therapy with [In-DTPA]octreotide: the Rotterdam experience. *Semin Nucl Med* 2002;**32**(2):110-22.

596. Valkema R, Pauwels S, Kvols LK, et al. Survival and response after peptide receptor radionuclide therapy with [90Y-DOTA0,Tyr3]octreotide in patients with advanced gastroenteropancreatic neuroendocrine tumors. *Semin Nucl Med* 2006;**36**(2):147-56.

597. Van Binnebeek S, Baete K, Vanbilloen B, et al. Individualized dosimetry-based activity reduction of ^{90}Y-DOTATOC prevents severe and rapid kidney function deterioration from peptide receptor radionuclide therapy. *Eur J Nucl Med Mol Imaging* 2014;**41**(6):1141-57.

598. Van Binnebeek S, Deroose CM, Baete K, et al. Altered biodistribution of somatostatin analogues after first cycle of Peptide receptor radionuclide therapy. *J Clin Oncol* 2011;**29**(19):e579-81.

599. Van Binnebeek S, Vanbilloen B, Baete K, et al. Comparison of diagnostic accuracy of (111)In-pentetreotide SPECT and (68)Ga-DOTATOC PET/CT: A lesion-by-lesion analysis in patients with metastatic neuroendocrine tumours. *Eur Radiol* 2016;**26**(3):900-9.

600. van der Zwan WA, Bodei L, Mueller-Brand J, de Herder WW, Kvols LK, Kwekkeboom DJ. GEPNETs update: radionuclide therapy in neuroendocrine tumors. *Eur J Endocrinol* 2015;**172**(1):R1-8.

601. van Eerd JE, Vegt E, Wetzels JF, et al. Gelatin-based plasma expander effectively reduces renal uptake of 111In-octreotide in mice and rats. *J Nucl Med* 2006;**47**(3):528-33.

602. van Essen M, Krenning EP, Kam BL, de Herder WW, van Aken MO, Kwekkeboom DJ. Report on short-term side effects of treatments with 177Lu-octreotate in combination with capecitabine in seven patients with gastroentero-pancreatic neuroendocrine tumours. *Eur J Nucl Med Mol Imaging* 2008;**35**(4):743-8.

603. van Essen M, Sundin A, Krenning EP, Kwekkeboom DJ. Neuroendocrine tumours: the role of imaging for diagnosis and therapy. *Nat Rev Endocrinol* 2014;**10**(2):102-14.

604. van Schaik E, van Vliet EI, Feelders RA, et al. Improved control of severe hypoglycemia in patients with malignant insulinomas by peptide receptor radionuclide therapy. *J Clin Endocrinol Metab* 2011;**96**(11):3381-9.

605. van Vliet EI, Teunissen JJ, Kam BL, de Jong M, Krenning EP, Kwekkeboom DJ. Treatment of gastroenteropancreatic neuroendocrine tumors with peptide receptor radionuclide therapy. *Neuroendocrinology* 2013;**97**(1):74-85.

606. Velikyan I, Sundin A, Sörensen J, et al. Quantitative and qualitative intrapatient comparison of 68Ga-DOTATOC and 68Ga-DOTATATE: net uptake rate for accurate quantification. *J Nucl Med* 2014;**55**(2):204-10.

607. Veltroni A, Cosaro E, Spada F, et al. Clinico-pathological features, treatments and survival of malignant insulinomas: a multicenter study. *Eur J Endocrinol* 2020;**182**(4):439-46.

608. Vida Navas EM, Martínez Lorca A, Sancho Gutiérrez A, et al. Case Report: Re-treatment with Lu-DOTATATE in neuroendocrine tumors. *Front Endocrinol (Lausanne)* 2021;**12**:676973.

609. Vija Racaru L, Sinigaglia M, Fontaine S, D'Aure D, Courbon F, Dierickx L. Diagnostic and therapeutic uptake of intrathyroid metastasis of midgut neuroendocrine tumor on 68Ga-DOTANOC PET/CT and 177Lu-DOTATATE imaging. *Clin Nucl Med* 2019;**44**(7):e445-e8.

610. Villard L, Romer A, Marincek N, et al. Cohort study of somatostatin-based radiopeptide therapy with [(90)Y-DOTA]-TOC versus [(90)Y-DOTA]-TOC plus [(177)Lu-DOTA]-TOC in neuroendocrine cancers. *J Clin Oncol* 2012;**30**(10):1100-6.

611. Vinjamuri S, Gilbert TM, Banks M, et al. Peptide receptor radionuclide therapy with (90)Y-DOTATATE/(90)Y-DOTATOC in patients with progressive metastatic neuroendocrine tumours: assessment of response, survival and toxicity. *Br J Cancer* 2013;**108**(7):1440-8.

612. von Hacht JL, Erdmann S, Niederstadt L, et al. Increasing molar activity by HPLC purification improves 68Ga-DOTA-NAPamide tumor accumulation in a B16/F1 melanoma xenograft model. *PLoS One* 2019;**14**(6):e0217883.

613. Wehrmann C, Senftleben S, Zachert C, Müller D, Baum RP. Results of individual patient dosimetry in peptide receptor radionuclide therapy with 177Lu DOTA-TATE and 177Lu DOTA-NOC. *Cancer Biother Radiopharm* 2007;**22**(3):406-16.

614. Wei X, Hain S, Hartmann L, Hirzebruch S, Ahmadzadehfar H. Dual imaging with 68Ga-DOTATOC and 18F-FDG PET for planning and follow-up of PRRT in combination with temozolomide treatment in a patient with a metastatic neuroendocrine tumor. *Clin Nucl Med* 2019;**44**(6):480-2.

615. Weikert T, Maas OC, Haas T, et al. Early prediction of treatment response of neuroendocrine hepatic metastases after peptide receptor radionuclide therapy with (90)Y-DOTATOC using diffusion weighted and dynamic contrast-enhanced MRI. *Contrast Media Mol Imaging* 2019;**2019**:1517208.

616. Werner RA, Brumberg J, Dierks A, et al. Paralytic subileus as an adverse effect of amino acid-based nephroprotection in a patient undergoing peptide receptor radionuclide therapy. *Clin Nucl Med* 2015;**40**(3):263-4.

617. Werner RA, Derlin T, Rowe SP, et al. high interobserver agreement for the standardized reporting system SSTR-RADS 1.0 on somatostatin receptor PET/CT. *J Nucl Med* 2021;**62**(4):514-20.

618. Werner RA, Ilhan H, Lehner S, et al. Correction to: pre-therapy somatostatin receptor-based heterogeneity predicts overall survival in pancreatic neuroendocrine tumor patients undergoing peptide receptor radionuclide therapy. *Mol Imaging Biol* 2018;**20**(6):1068.

619. Werner RA, Ilhan H, Lehner S, et al. Pre-therapy somatostatin receptor-based heterogeneity predicts overall survival in pancreatic neuroendocrine tumor patients undergoing peptide receptor radionuclide therapy. *Mol Imaging Biol* 2019;**21**(3):582-90.

620. Werner RA, Solnes LB, Javadi MS, et al. SSTR-RADS Version 1.0 as a reporting system for SSTR PET imaging and selection of potential PRRT candidates: a proposed standardization framework. *J Nucl Med* 2018;**59**(7):1085-91.

621. Werner RA, Weich A, Higuchi T, et al. Imaging of chemokine receptor 4 expression in neuroendocrine tumors - a triple tracer comparative approach. *Theranostics* 2017;**7**(6):1489-98.

622. Wetz C, Apostolova I, Steffen IG, et al. Predictive value of asphericity in pretherapeutic [(111)In]DTPA-octreotide SPECT/CT for response to peptide receptor radionuclide therapy with [(177)Lu]DOTATATE. *Mol Imaging Biol* 2017;**19**(3):437-45.

623. Wetz C, Genseke P, Apostolova I, et al. The association of intra-therapeutic heterogeneity of somatostatin receptor expression with morphological treatment response in patients undergoing PRRT with [177Lu]-DOTATATE. *PLoS One* 2019;**14**(5):e0216781.

624. Wetz C, Rogasch J, Genseke P, et al. Asphericity of somatostatin receptor expression in neuroendocrine tumors: an innovative predictor of outcome in everolimus treatment? *Diagnostics (Basel)* 2020;**10**(9):732.

625. White BE, Mujica-Mota R, Snowsill T, Gamper EM, Srirajaskanthan R, Ramage JK. Evaluating cost-effectiveness in the management of neuroendocrine neoplasms. *Rev Endocr Metab Disord* 2021;**22**(3):647-63.

626. Wibmer AG, Hricak H, Ulaner GA, Weber W. Trends in oncologic hybrid imaging. *Eur J Hybrid Imaging* 2018;**2**(1):1.

627. Wong PC, She WH, Khoo US, Cheung TT. A case of primary hepatic neuroendocrine tumor and literature review. *Case Rep Oncol* 2021;**14**(1):90-7.

628. Woodbridge LR, Murtagh BM, Yu DF, Planche KL. Midgut neuroendocrine tumors: imaging assessment for surgical resection. *Radiographics* 2014;**34**(2):413-26.

629. Woodburn KW, Fan Q, Kessel D, Luo Y, Young SW. Photodynamic therapy of B16F10 murine melanoma with lutetium texaphyrin. *J Invest Dermatol* 1998; **110**(5):746-51.

630. Wu Y, Pfeifer AK, Myschetzky R, et al. Induction of anti-tumor immune responses by peptide receptor radionuclide therapy with (177)Lu-DOTATATE in a murine model of a human neuroendocrine tumor. *Diagnostics (Basel)* 2013;**3**(4):344-55.

631. Xia Y, Zhang L, Wu H, Qiao L, Xia L. Primary hepatic neuroendocrine tumor with multiple liver metastases: a case report with literature review. *J Int Med Res* 2020;**48**(6):300060520932114.

632. Yadav MP, Ballal S, Bal C. Concomitant (177)Lu-DOTATATE and capecitabine therapy in malignant paragangliomas. *EJNMMI Res* 2019;**9**(1):13.

633. Yalchin M, Oliveira A, Theocharidou E, et al. The impact of radiological response to peptide receptor radionuclide therapy on overall survival in patients with metastatic midgut neuroendocrine tumors. *Clin Nucl Med* 2017;**42**(3):e135-e41.

634. Yazdanpanah O, Surapaneni S, Shanah L, Kabashneh S. Diagnosis and management of gastrointestinal neuroendocrine tumors: a comprehensive literature review. *Cureus* 2021;**13**(3):e14006.

635. Yilmaz E, Engin MN, Özkan ZG, et al. Y90 selective internal radiation therapy and peptide receptor radionuclide therapy for the treatment of metastatic neuroendocrine tumors: combination or not? *Nucl Med Commun* 2020;**41**(12):1242-9.

636. Yoo C, Oh CR, Kim ST, et al. Systemic treatment of advanced gastroenteropancreatic neuroendocrine tumors in Korea: literature review and expert opinion. *Cancer Res Treat* 2021;**53**(2):291-300.

637. Yordanova A, Mayer K, Essler M, Ahmadzadehfar H. Successful repeated peptide receptor radionuclide therapies in renal neuroendocrine tumor with osseous metastasis. *Clin Nucl Med* 2016;**41**(12):977-9.

638. Yordanova A, Wicharz MM, Mayer K, et al. The role of adding somatostatin analogues to peptide receptor radionuclide therapy as a combination and maintenance therapy. *Clin Cancer Res* 2018;**24**(19):4672-9.

639. Yu IS, Funk G, Lin E, Kennecke HF. the use of peptide receptor radionuclide therapy in patients with neuroendocrine tumor cardiac metastases. *Clin Nucl Med* 2021;**46**(1):e23-e6.

640. Yu NY, Rule WG, Sio TT, Ashman JB, Nelson KL. Radiation contamination following cremation of a deceased patient treated with a radiopharmaceutical. *JAMA* 2019;**321**(8):803-4.

641. Zandee WT, Brabander T, Blažević A, et al. Symptomatic and radiological response to 177Lu-DOTATATE for the treatment of functioning pancreatic neuroendocrine tumors. *J Clin Endocrinol Metab* 2019;**104**(4):1336-44.

642. Zandee WT, Brabander T, Blažević A, et al. Peptide receptor radionuclide therapy with 177Lu-DOTATATE for symptomatic control of refractory carcinoid syndrome. *J Clin Endocrinol Metab* 2021;**106**(9):e3665-72.

643. Zanini S, Renzi S, Giovinazzo F, Bermano G. mTOR pathway in gastroenteropancreatic neuroendocrine tumor (GEP-NETs). *Front Endocrinol (Lausanne)* 2020;**11**:562505.

644. Zerbi A, Capitanio V, Boninsegna L, et al. Treatment of malignant pancreatic neuroendocrine neoplasms: middle-term (2-year) outcomes of a prospective observational multicentre study. *HPB (Oxford)* 2013;**15**(12):935-43.

645. Zhang J, Kulkarni HR, Baum RP. Peptide receptor radionuclide therapy using 225Ac-DOTATOC achieves partial remission in a patient with progressive neuroendocrine liver metastases after repeated β-emitter peptide receptor radionuclide therapy. *Clin Nucl Med* 2020;**45**(3):241-3.

646. Zhang J, Kulkarni HR, Baum RP. Ectopic corticotropin-releasing hormone-secreting pancreatic neuroendocrine tumor: excellent response of liver metastases to peptide receptor radionuclide therapy as demonstrated by 68Ga-DOTATOC and 18F-FDG PET/CT imaging. *Clin Nucl Med* 2020;**45**(2):e125-e7.

647. Zhang J, Kulkarni HR, Singh A, Baum RP. Ten-year survival after peptide receptor radionuclide therapy of a metastatic well-differentiated G3 pancreatic neuroendocrine neoplasm. *Clin Nucl Med* 2018;**43**(9):676-8.

648. Zhang J, Kulkarni HR, Singh A, Baum RP. Delayed response (partial remission) 3 years after peptide receptor radionuclide therapy in a patient participating in the NETTER-1 Trial. *Clin Nucl Med* 2019;**44**(3):223-6.

649. Zhang J, Kulkarni HR, Singh A, Baum RP. successful intra-arterial peptide receptor radionuclide therapy of DOTATOC-negative high-grade liver metastases of a pancreatic neuroendocrine neoplasm using 177Lu-DOTA-LM3: a somatostatin receptor antagonist. *Clin Nucl Med* 2020;**45**(3):e165-e8.

650. Zhang J, Kulkarni HR, Singh A, Baum RP. Twelve-year survival of a patient with lymph node, pulmonary, bone, cardiac and intraspinal metastases of a rectal neuroendocrine neoplasm Treated with peptide receptor radionuclide therapy – the value of salvage peptide receptor radionuclide therapy. *Clin Nucl Med* 2020;**45**(4):e198-e200.

651. Zhang L, Vines DC, Scollard DA, et al. Correlation of somatostatin receptor-2 expression with gallium-68-DOTATATE uptake in neuroblastoma xenograft models. *Contrast Media Mol Imaging* 2017;**2017**:9481276.

652. Zhang P, Shen L. Implementation of multidisciplinary team in the diagnosis and treatment of gastroenteropancreatic neuroendocrine neoplasm [article in Chinese]. *Zhonghua Wei Chang Wai Ke Za Zhi* 2016;**19**(11):1205-10.

653. Zhao W, Esquinas PL, Frezza A, Hou X, Beauregard JM, Celler A. Accuracy of kidney dosimetry performed using simplified time activity curve modelling methods: a (177)Lu-DOTATATE patient study. *Phys Med Biol* 2019;**64**(17):175006.

654. Zhu M, Bassam Sonbol M, Halfdanarson T, et al. Homologous recombination repair defect may predict treatment response to peptide receptor radionuclide therapy for neuroendocrine tumors. *Oncologist* 2020;**25**(8):e1246-e8.

Excluded records following assessment for eligibility (385 excluded of 403 assessed), by exclusion reason

In instances where records reported non-separable treatment data **and** non-separable data between different NET types, the record was excluded and moved to the “non-separable treatment” category.

Non-separable data between different NETs (n=85)

1. Adnan A, Sampathirao N, Basu S. Implications of fluorodeoxyglucose uptake in low-intermediate grade metastatic neuroendocrine tumors from peptide receptor radionuclide therapy outcome viewpoint: a semi-quantitative standardized uptake value-based analysis. *World J Nucl Med* 2019;**18**(4):389-95.

2. Al-Toubah T, Strosberg J. Peptide receptor radiotherapy comes of age. *Endocrinol Metab Clin North Am* 2018;**47**(3):615-25.

3. Alevroudis E, Spei ME, Chatziioannou SN, et al. Clinical utility of (18)F-FDG PET in neuroendocrine tumors prior to peptide receptor radionuclide therapy: a systematic review and meta-analysis. *Cancers (Basel)* 2021;**13**(8):1813.

4. Ballal S, Yadav MP, Damle NA, Sahoo RK, Bal C. Concomitant 177Lu-DOTATATE and capecitabine therapy in patients with advanced neuroendocrine tumors: a long-term-outcome, toxicity, survival, and quality-of-life study. *Clin Nucl Med* 2017;**42**(11):e457-e66.

5. Baum RP, Kulkarni HR, Carreras C. Peptides and receptors in image-guided therapy: theranostics for neuroendocrine neoplasms. *Semin Nucl Med* 2012;**42**(3):190-207.

6. Black JRM, Atkinson SR, Singh A, Evans J, Sharma R. The Inflammation-Based Index Can predict response and improve patient selection in NETs treated with PRRT: a pilot study. *J Clin Endocrinol Metab* 2019;**104**(2):285-92.

7. Bodei L, Ferone D, Grana CM, et al. Peptide receptor therapies in neuroendocrine tumors. *J Endocrinol Invest* 2009;**32**(4):360-9.

8. Bodei L, Kidd MS, Singh A, et al. PRRT neuroendocrine tumor response monitored using circulating transcript analysis: the NETest. *Eur J Nucl Med Mol Imaging* 2020;**47**(4):895-906.
9. Bodei L, Pepe G, Paganelli G. Peptide receptor radionuclide therapy (PRRT) of neuroendocrine tumors with somatostatin analogues. *Eur Rev Med Pharmacol Sci* 2010;**14**(4):347-51.
10. Brabander T, Teunissen JJ, Van Eijck CH, et al. Peptide receptor radionuclide therapy of neuroendocrine tumours. *Best Pract Res Clin Endocrinol Metab* 2016;**30**(1):103-14.
11. Brabander T, van der Zwan WA, Teunissen JJM, et al. Long-term efficacy, survival, and safety of [(177)Lu-DOTA(0),Tyr(3)]octreotate in patients with gastroenteropancreatic and bronchial neuroendocrine tumors. *Clin Cancer Res* 2017;**23**(16):4617-24.
12. Buscombe JR. Evidence base for the use of PRRT. *Semin Nucl Med* 2020;**50**(5):399-404.
13. Caplin ME, Ratnayake GM. Diagnostic and therapeutic advances in neuroendocrine tumours. *Nat Rev Endocrinol* 2021;**17**(2):81-2.
14. Chen L, Navalkissoor S, Quigley AM, et al. (177)Lu-DOTATATE in older patients with metastatic neuroendocrine tumours: safety, efficacy and health-related quality of life. *Eur J Nucl Med Mol Imaging* 2021;**48**(11):3582-94.
15. Danthala M, Kallur KG, Prashant GR, Rajkumar K, Raghavendra Rao M. (177)Lu-DOTATATE therapy in patients with neuroendocrine tumours: 5 years' experience from a tertiary cancer care centre in India. *Eur J Nucl Med Mol Imaging* 2014;**41**(7):1319-26.
16. Das S, Du L, Schad A, et al. A clinical score for neuroendocrine tumor patients under consideration for Lu-177-DOTATATE therapy. *Endocr Relat Cancer* 2021;**28**(3):203-12.
17. Dash A, Chakraborty S, Pillai MR, Knapp FF, Jr. Peptide receptor radionuclide therapy: an overview. *Cancer Biother Radiopharm* 2015;**30**(2):47-71.
18. de Lima BM, de Carvalho JR, Gaspar PR, Pujatti PB, Gonçalves R. Liver steatosis secondary to PRRT with 177Lu-DOTATATE: an unknown adverse effect. *Clin Nucl Med* 2020;**45**(5):374-6.
19. Del Prete M, Buteau FA, Arsenault F, et al. Personalized (177)Lu-octreotate peptide receptor radionuclide therapy of neuroendocrine tumours: initial results from the P-PRRT trial. *Eur J Nucl Med Mol Imaging* 2019;**46**(3):728-42.
20. Erbas B, Tuncel M. Renal function assessment during peptide receptor radionuclide therapy. *Semin Nucl Med* 2016;**46**(5):462-78.
21. Faggiano A, Lo Calzo F, Pizza G, Modica R, Colao A. The safety of available treatments options for neuroendocrine tumors. *Expert Opin Drug Saf* 2017;**16**(10):1149-61.
22. Forrer F, Valkema R, Kwekkeboom DJ, de Jong M, Krenning EP. Neuroendocrine tumors. Peptide receptor radionuclide therapy. *Best Pract Res Clin Endocrinol Metab* 2007;**21**(1):111-29.
23. Frilling A, Weber F, Cicinnati V, Broelsch C. Role of radiolabeled octreotide therapy in patients with metastatic neuroendocrine neoplasms. *Expert Rev Endocrinol Metab* 2007;**2**(4):517-27.
24. Gabriel M. Radionuclide therapy beyond radioiodine. *Wien Med Wochenschr* 2012;**162**(19-20):430-9.
25. Gangi A, Howe JR. The Landmark Series: neuroendocrine tumor liver metastases. *Ann Surg Oncol* 2020;**27**(9):3270-80.
26. Garkavij M, Nickel M, Sjögreen-Gleisner K, et al. 177Lu-[DOTA0,Tyr3] octreotate therapy in patients with disseminated neuroendocrine tumors: analysis of dosimetry with impact on future therapeutic strategy. *Cancer* 2010;**116**(4 Suppl):1084-92.
27. Giovacchini G, Nicolas G, Forrer F. Peptide receptor radionuclide therapy with somatostatin analogues in neuroendocrine tumors. *Anticancer Agents Med Chem* 2012;**12**(5):526-42.
28. Gupta SK, Singla S, Bal C. Renal and hematological toxicity in patients of neuroendocrine tumors after peptide receptor radionuclide therapy with 177Lu-DOTATATE. *Cancer Biother Radiopharm* 2012;**27**(9):593-9.
29. Hagmarker L, Svensson J, Rydén T, et al. Bone marrow absorbed doses and correlations with hematologic response during (177)Lu-DOTATATE treatments are influenced by image-based dosimetry method and presence of skeletal metastases. *J Nucl Med* 2019;**60**(10):1406-13.
30. Haider M, Al-Toubah T, El-Haddad G, Strosberg J. Molecular imaging and radionuclide therapy of neuroendocrine tumors. *Curr Opin Endocrinol Diabetes Obes* 2020;**27**(1):16-21.
31. Haug AR, Auernhammer CJ, Wängler B, et al. 68Ga-DOTATATE PET/CT for the early prediction of response to somatostatin receptor-mediated radionuclide therapy in patients with well-differentiated neuroendocrine tumors. *J Nucl Med* 2010;**51**(9):1349-56.

32. Heckert JM, Kipnis ST, Kumar S, et al. abnormal pretreatment liver function tests are associated with discontinuation of peptide receptor radionuclide therapy in a U.S.-based neuroendocrine tumor cohort. *Oncologist* 2020;**25**(7):572-8.

33. Hörsch D, Ezziddin S, Haug A, et al. Peptide receptor radionuclide therapy for neuroendocrine tumors in Germany: first results of a multi-institutional cancer registry. *Recent Results Cancer Res* 2013;**194**:457-65.

34. Hubble D, Kong G, Michael M, Johnson V, Ramdave S, Hicks RJ. 177Lu-octreotate, alone or with radiosensitising chemotherapy, is safe in neuroendocrine tumour patients previously treated with high-activity 111In-octreotide. *Eur J Nucl Med Mol Imaging* 2010;**37**(10):1869-75.

35. Huizing DMV, Aalbersberg EA, Versleijen MWJ, et al. Early response assessment and prediction of overall survival after peptide receptor radionuclide therapy. *Cancer Imaging* 2020;**20**(1):57.

36. Khan S, Krenning EP, van Essen M, Kam BL, Teunissen JJ, Kwekkeboom DJ. Quality of life in 265 patients with gastroenteropancreatic or bronchial neuroendocrine tumors treated with [177Lu-DOTA0,Tyr3]octreotate. *J Nucl Med* 2011;**52**(9):1361-8.

37. Kunikowska J, Królicki L, Sowa-Staszczak A, et al. Polish experience in peptide receptor radionuclide therapy. *Recent Results Cancer Res* 2013;**194**:467-78.

38. Kwekkeboom DJ, Bakker WH, Kam BL, et al. Treatment of patients with gastro-entero-pancreatic (GEP) tumours with the novel radiolabelled somatostatin analogue [177Lu-DOTA(0),Tyr3]octreotate. *Eur J Nucl Med Mol Imaging* 2003;**30**(3):417-22.

39. Lapa C, Werner RA, Bluemel C, et al. Prediction of clinically relevant hyperkalemia in patients treated with peptide receptor radionuclide therapy. *EJNMMI Res* 2014;**4**:74.

40. Liu AJ, Ueberroth BE, McGarrah PW, et al. Treatment outcomes of well-differentiated high-grade neuroendocrine tumors. *Oncologist* 2021;**26**(5):383-8.

41. Liu Q, Cheng Y, Zang J, et al. Dose escalation of an Evans blue-modified radiolabeled somatostatin analog (177)Lu-DOTA-EB-TATE in the treatment of metastatic neuroendocrine tumors. *Eur J Nucl Med Mol Imaging* 2020;**47**(4):947-57.

42. Løitegård T, Berntzen DT, Thiis-Evensen E. The RECIST criteria compared to conventional response evaluation after peptide receptor radionuclide therapy in patients with neuroendocrine neoplasms. *Ann Nucl Med* 2019;**33**(3):147-52.

43. Löser A, Schwarzenböck SM, Heuschkel M, Willenberg HS, Krause BJ, Kurth J. Peptide receptor radionuclide therapy with 177Lu-DOTA-octreotate: dosimetry, nephrotoxicity, and the effect of hematological toxicity on survival. *Nucl Med Commun* 2018;**39**(3):236-46.

44. Medaer E, Verslype C, Van Cutsem E, et al. Influence of pretreatment with everolimus or sunitinib on the subacute hematotoxicity of (177)Lu-DOTATATE PRRT. *Acta Oncol* 2020;**59**(6):644-51.

45. Modlin IM, Kidd M, Malczewska A, et al. The NETest: The clinical utility of multigene blood analysis in the diagnosis and management of neuroendocrine tumors. *Endocrinol Metab Clin North Am* 2018;**47**(3):485-504.

46. Nilica B, Waitz D, Stevanovic V, et al. Direct comparison of (68)Ga-DOTA-TOC and (18)F-FDG PET/CT in the follow-up of patients with neuroendocrine tumour treated with the first full peptide receptor radionuclide therapy cycle. *Eur J Nucl Med Mol Imaging* 2016;**43**(9):1585-92.

47. Ortega C, Wong RK, Schaefferkoetter J, et al. Quantitative (68)Ga-DOTATATE PET/CT parameters for the prediction of therapy response in patients with progressive metastatic neuroendocrine tumors treated with (177)Lu-DOTATATE. *J Nucl Med* 2021;**62**(10):1406-14.

48. Pencharz D, Walker M, Yalchin M, et al. Early efficacy of and toxicity from lutetium-177-DOTATATE treatment in patients with progressive metastatic NET. *Nucl Med Commun* 2017;**38**(7):593-600.

49. Ranade R, Basu S. 177Lu-DOTATATE PRRT in patients with metastatic neuroendocrine tumor and a single functioning kidney: tolerability and effect on renal function. *J Nucl Med Technol* 2016;**44**(2):65-9.

50. Rudisile S, Gosewisch A, Wenter V, et al. Salvage PRRT with (177)Lu-DOTA-octreotate in extensively pretreated patients with metastatic neuroendocrine tumor (NET): dosimetry, toxicity, efficacy, and survival. *BMC Cancer* 2019;**19**(1):788.

51. Sabet A, Biersack HJ, Ezziddin S. Advances in peptide receptor radionuclide therapy. *Semin Nucl Med* 2016;**46**(1):40-6.

52. Sabet A, Ezziddin K, Pape UF, et al. Long-term hematotoxicity after peptide receptor radionuclide therapy with 177Lu-octreotate. *J Nucl Med* 2013;**54**(11):1857-61.

53. Sarnelli A, Guerriero F, Botta F, et al. Therapeutic schemes in 177Lu and 90Y-PRRT: radiobiological considerations. *Q J Nucl Med Mol Imaging* 2017;**61**(2):216-31.

54. Shaheen S, Moradi F, Gamino G, Kunz PL. Patient selection and toxicities of PRRT for metastatic neuroendocrine tumors and research opportunities. *Curr Treat Options Oncol* 2020;**21**(4):25.

55. Sharma R, Wang WM, Yusuf S, et al. (68)Ga-DOTATATE PET/CT parameters predict response to peptide receptor radionuclide therapy in neuroendocrine tumours. *Radiother Oncol* 2019;**141**:108-15.

56. Sitani K, Parghane RV, Talole S, Basu S. Long-term outcome of indigenous (177)Lu-DOTATATE PRRT in patients with metastatic advanced neuroendocrine tumours: a single institutional observation in a large tertiary care setting. *Br J Radiol* 2021;**94**(1117):20201041.

57. Sonbol MB, Halfdanarson TR, Hilal T. Assessment of therapy-related myeloid neoplasms in patients with neuroendocrine tumors after peptide receptor radionuclide therapy: a systematic review. *JAMA Oncol* 2020;**6**(7):1086-92.

58. Sundlöv A, Sjögreen-Gleisner K, Svensson J, et al. Individualised (177)Lu-DOTATATE treatment of neuroendocrine tumours based on kidney dosimetry. *Eur J Nucl Med Mol Imaging* 2017;**44**(9):1480-9.

59. Sundlöv A, Sjögreen-Gleisner K, Tennvall J, et al. Pituitary function after high-dose 177Lu-DOTATATE therapy and long-term follow-up. *Neuroendocrinology* 2021;**111**(4):344-53.

60. Svensson J, Berg G, Wängberg B, Larsson M, Forssell-Aronsson E, Bernhardt P. Renal function affects absorbed dose to the kidneys and haematological toxicity during ^{177}Lu-DOTATATE treatment. *Eur J Nucl Med Mol Imaging* 2015;**42**(6):947-55.

61. Thiis-Evensen E, Poole AC, Nguyen HT, Sponheim J. Achieving objective response in treatment of non-resectable neuroendocrine tumors does not predict longer time to progression compared to achieving stable disease. *BMC Cancer* 2020;**20**(1):466.

62. van Essen M, Krenning EP, Kam BL, de Herder WW, Feelders RA, Kwekkeboom DJ. Salvage therapy with (177)Lu-octreotate in patients with bronchial and gastroenteropancreatic neuroendocrine tumors. *J Nucl Med* 2010;**51**(3):383-90.

63. van Vliet EI, de Herder WW, de Rijke YB, et al. Hypocalcaemia after treatment with [177Lu-DOTA 0,Tyr3]octreotate. *Eur J Nucl Med Mol Imaging* 2013;**40**(12):1843-52.

64. van Vliet EI, Krenning EP, Teunissen JJ, Bergsma H, Kam BL, Kwekkeboom DJ. Comparison of response evaluation in patients with gastroenteropancreatic and thoracic neuroendocrine tumors after treatment with [177Lu-DOTA0,Tyr3]octreotate. *J Nucl Med* 2013;**54**(10):1689-96.

65. Wang H, Cheng Y, Zhang J, et al. Response to single low-dose (177)Lu-DOTA-EB-TATE treatment in patients with advanced neuroendocrine neoplasm: a prospective pilot study. *Theranostics* 2018;**8**(12):3308-16.

66. Werner RA, Beykan S, Higuchi T, et al. The impact of 177Lu-octreotide therapy on 99mTc-MAG3 clearance is not predictive for late nephropathy. *Oncotarget* 2016;**7**(27):41233-41.

67. Werner RA, Bluemel C, Lassmann M, et al. SPECT- and PET-based patient-tailored treatment in neuroendocrine tumors: a comprehensive multidisciplinary team approach. *Clin Nucl Med* 2015;**40**(5):e271-7.

68. Werner RA, Lapa C, Ilhan H, et al. Survival prediction in patients undergoing radionuclide therapy based on intratumoral somatostatin-receptor heterogeneity. *Oncotarget* 2017;**8**(4):7039-49.

69. Yao JC, Catena L, Colao A, Paganelli G. Perspectives in the development of novel treatment approaches. *Tumori* 2010;**96**(5):858-73.

70. Yordanova A, Mayer K, Brossart P, et al. Safety of multiple repeated cycles of (177)Lu-octreotate in patients with recurrent neuroendocrine tumour. *Eur J Nucl Med Mol Imaging* 2017;**44**(7):1207-14.

71. Zhang J, Kulkarni HR, Singh A, Niepsch K, Müller D, Baum RP. Peptide receptor radionuclide therapy in grade 3 neuroendocrine neoplasms: safety and survival analysis in 69 patients. *J Nucl Med* 2019;**60**(3):377-85.

72. Zhang J, Song Q, Cai L, Xie Y, Chen Y. The efficacy of (177)Lu-DOTATATE peptide receptor radionuclide therapy (PRRT) in patients with metastatic neuroendocrine tumours: a systematic review and meta-analysis. *J Cancer Res Clin Oncol* 2020;**146**(6):1533-43.

73. Bodei L, Cremonesi M, Grana CM, et al. Peptide receptor radionuclide therapy with ^{177}Lu-DOTATATE: the IEO phase I-II study. *Eur J Nucl Med Mol Imaging* 2011;**38**(12):2125-35.

74. Bodei L, Kidd M, Modlin IM, et al. Measurement of circulating transcripts and gene cluster analysis predicts and defines therapeutic efficacy of peptide receptor radionuclide therapy (PRRT) in neuroendocrine tumors. *Eur J Nucl Med Mol Imaging* 2016;**43**(5):839-51.

75. Bodei L, Kidd M, Paganelli G, et al. Long-term tolerability of PRRT in 807 patients with neuroendocrine tumours: the value and limitations of clinical factors. *Eur J Nucl Med Mol Imaging* 2015;**42**(1):5-19.

76. Demirci E, Kabasakal L, Toklu T, et al. 177Lu-DOTATATE therapy in patients with neuroendocrine tumours including high-grade (WHO G3) neuroendocrine tumours: response to treatment and long-term survival update. *Nucl Med Commun* 2018;**39**(8):789-96.
77. Lemelin A, Maucort-Boulch D, Castel-Kremer E, et al. Elderly patients with metastatic neuroendocrine tumors are undertreated and have shorter survival: the LyREMeNET study. *Neuroendocrinology* 2020;**110**(7-8):653-61.
78. Lin E, Chen T, Little A, et al. Safety and outcomes of (177) Lu-DOTATATE for neuroendocrine tumours: experience in New South Wales, Australia. *Intern Med J* 2019;**49**(10):1268-77.
79. Martin AI, Horgan N. Radiolabelled somatostatin analogue treatment in a case of carcinoid tumour with choroidal metastases. *Eye (Lond)* 2012;**26**(12):1596-8.
80. Sistani G, Sutherland DEK, Mujoomdar A, et al. Efficacy of (177)Lu-Dotatate induction and maintenance therapy of various types of neuroendocrine tumors: a phase II registry study. *Curr Oncol* 2020;**28**(1):115-27.
81. Vaghaiwalla T, Ruhle B, Memeh K, et al. Response rates in metastatic neuroendocrine tumors receiving peptide receptor radionuclide therapy and implications for future treatment strategies. *Surgery* 2021;**169**(1):162-7.
82. van der Zwan WA, Brabander T, Kam BLR, et al. Salvage peptide receptor radionuclide therapy with [(177)Lu-DOTA,Tyr(3)]octreotate in patients with bronchial and gastroenteropancreatic neuroendocrine tumours. *Eur J Nucl Med Mol Imaging* 2019;**46**(3):704-17.
83. Vaughan E, Machta J, Walker M, Toumpanakis C, Caplin M, Navalkissoor S. Retreatment with peptide receptor radionuclide therapy in patients with progressing neuroendocrine tumours: efficacy and prognostic factors for response. *Br J Radiol* 2018;**91**(1091):20180041.
84. Vorster M, Modiselle MR, Corbett CS, Lawal IO, Buscombe JR, Sathekge MM. First results and experience with PRRT in South Africa. *World J Nucl Med* 2018;**17**(2):86-93.
85. Hamiditabar M, Ali M, Bolek L, Vahdati G, Tworowska I, Delpassand ES. Safety and effectiveness of 177Lu-DOTATATE peptide receptor radionuclide therapy after regional hepatic embolization in patients with somatostatin-expressing neuroendocrine tumors. *Clin Nucl Med* 2017;**42**(11):822-8.

Did not report outcome of interest (n=70)

1. Al-Jundi M, Thakur S, Gubbi S, Klubo-Gwiezdzinska J. Novel targeted therapies for metastatic thyroid cancer – a comprehensive review. *Cancers (Basel)* 2020;**12**(8):2104.
2. Ambrosini V, Kunikowska J, Baudin E, et al. Consensus on molecular imaging and theranostics in neuroendocrine neoplasms. *Eur J Cancer* 2021;**146**:56-73.
3. Ansquer C, Kraeber-Bodéré F, Chatal JF. Current status and perspectives in peptide receptor radiation therapy. *Curr Pharm Des* 2009;**15**(21):2453-62.
4. Ballinger JR. Theranostic radiopharmaceuticals: established agents in current use. *Br J Radiol* 2018;**91**(1091):20170969.
5. Basu S, Abhyankar A, Kand P, et al. 'Reverse discordance' between 68Ga-DOTA-NOC PET/CT and 177Lu-DOTA-TATE posttherapy scan: the plausible explanations and its implications for high-dose therapy with radiolabeled somatostatin receptor analogs. *Nucl Med Commun* 2011;**32**(7):654-8.
6. Bautista PA. The emergence of theranostics in the Philippines: overcoming challenges and bringing hope. *Nucl Med Mol Imaging* 2019;**53**(1):30-2.
7. Bodei L, Kidd M, Modlin IM, et al. Gene transcript analysis blood values correlate with ^{68}Ga-DOTA-somatostatin analog (SSA) PET/CT imaging in neuroendocrine tumors and can define disease status. *Eur J Nucl Med Mol Imaging* 2015;**42**(9):1341-52.
8. Bodei L, Mueller-Brand J, Baum RP, et al. The joint IAEA, EANM, and SNMMI practical guidance on peptide receptor radionuclide therapy (PRRNT) in neuroendocrine tumours. *Eur J Nucl Med Mol Imaging* 2013;**40**(5):800-16.
9. Bodei L, Schöder H, Baum RP, et al. Molecular profiling of neuroendocrine tumours to predict response and toxicity to peptide receptor radionuclide therapy. *Lancet Oncol* 2020;**21**(9):e431-e43.
10. Bongiovanni A, Recine F, Celli M, et al. Osteoblastic bone metastases from neuroendocrine tumor (NET) of unknown origin detected by 18fluorocholine PET/CT and its comparison with 68gallium-DOTATOC PET/CT: Case report and review of the literature. *Medicine (Baltimore)* 2017;**96**(46):e8567.
11. Brabander T, Nonnekens J, Hofland J. The next generation of peptide receptor radionuclide therapy. *Endocr Relat Cancer* 2019;**26**(8): C7-c11.

12. Brogsitter C, Hartmann H, Wunderlich G, Schottelius M, Wester HJ, Kotzerke J. Twins in spirit part IV - [(177)Lu] high affinity DOTATATE. A promising new tracer for peptide receptor radiotherapy? *Nuklearmedizin* 2017;**56**(1):1-8.

13. Carollo A, Papi S, Chinol M. Lutetium-177 labeled peptides: the European Institute of Oncology experience. *Curr Radiopharm* 2016;**9**(1):19-32.

14. Casciano R, Wang X, Stern L, et al. International practice patterns and resource utilization in the treatment of neuroendocrine tumors. *Pancreas* 2013;**42**(2):339-47.

15. Chicheportiche A, Grozinsky-Glasberg S, Gross DJ, et al. Predictive power of the post-treatment scans after the initial or first two courses of [(177)Lu]-DOTA-TATE. *EJNMMI Phys* 2018;**5**(1):36.

16. Clement D, Ramage J, Srirajaskanthan R. Update on pathophysiology, treatment, and complications of carcinoid syndrome. *J Oncol* 2020;**2020**:8341426.

17. Damle NA, Bal C, Gupta S, Singhal A. Discordance in 68Ga-DOTANOC and 177Lu-DOTATATE uptake in diagnostic and post-therapy scans in patients with medullary thyroid cancer–likely reasons. *J Cancer Res Ther* 2013;**9**(4):754-5.

18. de Jong M, Krenning E. New advances in peptide receptor radionuclide therapy. *J Nucl Med* 2002;**43**(5):617-20.

19. Delbeke D, Graham MM. Addendum to the editorial "Joint guidance on peptide receptor radionuclide therapy in neuroendocrine tumors". *J Nucl Med* 2013;**54**(7):1170.

20. Delbeke D, Graham MM. Joint guidance on peptide receptor radionuclide therapy in neuroendocrine tumors. *J Nucl Med* 2013;**54**(5):663.

21. Demirkan BH, Eriksson B. Systemic treatment of neuroendocrine tumors with hepatic metastases. *Turk J Gastroenterol* 2012;**23**(5):427-37.

22. Dijkgraaf I, Boerman OC, Oyen WJ, Corstens FH, Gotthardt M. Development and application of peptide-based radiopharmaceuticals. *Anticancer Agents Med Chem* 2007;**7**(5):543-51.

23. Ehlerding EB, Lan X, Cai W. "Albumin Hitchhiking" with an Evans Blue Analog for Cancer Theranostics. *Theranostics* 2018;**8**(3):812-4.

24. Erovic BM, Kim D, Cassol C, et al. Prognostic and predictive markers in medullary thyroid carcinoma. *Endocr Pathol* 2012;**23**(4):232-42.

25. Esser JP, Krenning EP, Teunissen JJ, et al. Comparison of [(177)Lu-DOTA(0),Tyr(3)]octreotate and [(177)Lu-DOTA(0),Tyr(3)]octreotide: which peptide is preferable for PRRT? *Eur J Nucl Med Mol Imaging* 2006;**33**(11):1346-51.

26. Eychenne R, Bouvry C, Bourgeois M, Loyer P, Benoist E, Lepareur N. Overview of radiolabeled somatostatin analogs for cancer imaging and therapy. *Molecules* 2020;**25**(17):4012.

27. Ezziddin S, Lohmar J, Yong-Hing CJ, et al. Does the pretherapeutic tumor SUV in 68Ga DOTATOC PET predict the absorbed dose of 177Lu octreotate? *Clin Nucl Med* 2012;**37**(6):e141-7.

28. Fazio N, Ungaro A, Spada F, et al. The role of multimodal treatment in patients with advanced lung neuroendocrine tumors. *J Thorac Dis* 2017;**9**(Suppl 15):S1501-s10.

29. Feijtel D, de Jong M, Nonnekens J. Peptide receptor radionuclide therapy: looking back, looking forward. *Curr Top Med Chem* 2020;**20**(32):2959-69.

30. Filippov A, Bonjoc KC, Chea J, Bowles N, Poku E, Chaudhry A. Role of theranostics in thoracic oncology. *J Thorac Dis* 2020;**12**(9):5140-6.

31. Garcia-Alvarez A, Hernando Cubero J, Capdevila J. Drug development in neuroendocrine tumors: what is on the horizon? *Curr Treat Options Oncol* 2021;**22**(5):43.

32. Gleisner KS, Brolin G, Sundlöv A, et al. Long-term retention of 177Lu/177mLu-DOTATATE in patients investigated by γ-spectrometry and γ-camera imaging. *J Nucl Med* 2015;**56**(7):976-84.

33. Goldsmith SJ. Targeted radionuclide therapy: a historical and personal review. *Semin Nucl Med* 2020;**50**(1):87-97.

34. González-Flores E, Serrano R, Sevilla I, et al. SEOM clinical guidelines for the diagnosis and treatment of gastroenteropancreatic and bronchial neuroendocrine neoplasms (NENs) (2018). *Clin Transl Oncol* 2019;**21**(1):55-63.

35. Gupta SK, Singla S, Thakral P, Bal CS. Dosimetric analyses of kidneys, liver, spleen, pituitary gland, and neuroendocrine tumors of patients treated with 177Lu-DOTATATE. *Clin Nucl Med* 2013;**38**(3):188-94.

36. Hennrich U, Kopka K. Lutathera(®): The first FDA- and EMA-approved radiopharmaceutical for peptide receptor radionuclide therapy. *Pharmaceuticals (Basel)* 2019;**12**(3):114.

37. Hicks RJ. Use of molecular targeted agents for the diagnosis, staging and therapy of neuroendocrine malignancy. *Cancer Imaging* 2010;**10**(Spec no A[1a]):S83-91.

38. Hicks RJ. The difference between medicine and magic is that magicians know what they are doing. *Eur J Nucl Med Mol Imaging* 2015;**42**(1):1-4.

39. Hofman MS, Hicks RJ. Peptide receptor radionuclide therapy for neuroendocrine tumours: standardized and randomized, or personalized? *Eur J Nucl Med Mol Imaging* 2014;**41**(2):211-3.

40. Hörsch D, Kulkarni HR, Baum RP. THERANOSTICS-clinical aimshots in surgical warfare against well-differentiated neuroendocrine neoplasms. *Ann Transl Med* 2014;**2**(1):1.

41. Jiménez-Franco LD, Kletting P, Beer AJ, Glatting G. Treatment planning algorithm for peptide receptor radionuclide therapy considering multiple tumor lesions and organs at risk. *Med Phys* 2018; Jun 15. doi: 10.1002/mp.13049.

42. Kamaleshwaran KK, Joseph J, Upadhya I, Shinto AS. Image findings of a rare case of neuroendocrine tumor metastatic to orbital extraocular muscle in gallium-68 DOTANOC positron emission tomography/computed tomography and therapy with lutetium-177 DOTATATE. *Indian J Nucl Med* 2017;**32**(2):125-7.

43. Kedia R, Hansen N, Goldner W. Nuclear imaging in metastatic paraganglioma. *J Nucl Med Technol* 2016;**44**(4):251-2.

44. Kidd M, Modlin IM. Therapy: The role of liquid biopsies to manage and predict PRRT for NETs. *Nat Rev Gastroenterol Hepatol* 2017;**14**(6):331-2.

45. Kollar A, Bütikofer L, Ochsenbein A, Stettler C, Trepp R. Treatment sequence in patients with neuroendocrine tumours: a nationwide multicentre, observational analysis of the Swiss neuroendocrine tumour registry. *Swiss Med Wkly* 2020;**150**:w20176.

46. Krenning EP, Kwekkeboom DJ, Valkema R, Pauwels S, Kvols LK, De Jong M. Peptide receptor radionuclide therapy. *Ann N Y Acad Sci* 2004;**1014**:234-45.

47. Kulkarni HR, Baum RP. Theranostics with Ga-68 somatostatin receptor PET/CT: monitoring response to peptide receptor radionuclide therapy. *PET Clin* 2014;**9**(1):91-7.

48. Kulkarni HR, Singh A, Baum RP. Advances in the diagnosis of neuroendocrine neoplasms. *Semin Nucl Med* 2016;**46**(5):395-404.

49. Kunikowska J, Królicki L. Targeted α-emitter therapy of neuroendocrine tumors. *Semin Nucl Med* 2020;**50**(2):171-6.

50. Kwekkeboom DJ, Bakker WH, Kooij PP, et al. [177Lu-DOTAOTyr3]octreotate: comparison with [111In-DTPAo]octreotide in patients. *Eur J Nucl Med* 2001;**28**(9):1319-25.

51. Lapa C. Exciting opportunities in nuclear medicine imaging and therapy. *J Clin Med* 2019;**8**(11):1944.

52. Lapa C, Werner RA, Schmid JS, et al. Prognostic value of positron emission tomography-assessed tumor heterogeneity in patients with thyroid cancer undergoing treatment with radiopeptide therapy. *Nucl Med Biol* 2015;**42**(4):349-54.

53. Lehman JM, Gwin ME, Massion PP. Immunotherapy and targeted therapy for small cell lung cancer: there is hope. *Curr Oncol Rep* 2017;**19**(7):49.

54. Lehrman ED, Fidelman N. Liver-directed therapy for neuroendocrine tumor liver metastases in the era of peptide receptor radionuclide therapy. *Semin Intervent Radiol* 2020;**37**(5):499-507.

55. Malczewska A, Kos-Kudła B, Kidd M, et al. The clinical applications of a multigene liquid biopsy (NETest) in neuroendocrine tumors. *Adv Med Sci* 2020;**65**(1):18-29.

56. Nicolas GP, Morgenstern A, Schottelius M, Fani M. New developments in peptide receptor radionuclide therapy. *J Nucl Med* 2018; Dec 20. doi: 10.2967/jnumed.118.213496.

57. Oberg K. Molecular imaging radiotherapy: theranostics for personalized patient management of neuroendocrine tumors (NETs). *Theranostics* 2012;**2**(5):448-58.

58. Panagiotidis E, Alshammari A, Michopoulou S, et al. Comparison of the impact of 68Ga-DOTATATE and 18F-FDG PET/CT on clinical management in patients with neuroendocrine tumors. *J Nucl Med* 2017;**58**(1):91-6.

59. Parus JL, Mikolajczak R. Beta-emitting radionuclides for peptide receptor radionuclide therapy. *Curr Top Med Chem* 2012;**12**(23):2686-93.

60. Paulson AS, Bergsland EK. Systemic therapy for advanced carcinoid tumors: where do we go from here? *J Natl Compr Canc Netw* 2012;**10**(6):785-93.

61. Plante A, Baudin E, Do Cao C, et al. Patient-reported tolerance in treatments approved in neuroendocrine tumors: a national survey from the French Group of Endocrine Tumors. *Clin Res Hepatol Gastroenterol* 2018;**42**(2):153-9.

62. Pool SE, Krenning EP, Koning GA, et al. Preclinical and clinical studies of peptide receptor radionuclide therapy. *Semin Nucl Med* 2010;**40**(3):209-18.

63. Şimşek DH, Şanlı Y, Kuyumcu S, Başaran B, Mudun A. (68)Ga-DOTATATE PET-CT imaging in carotid body paragangliomas. *Ann Nucl Med* 2018;**32**(4):297-301.
64. Singh N, Krishna B, Vyas M, et al. Lutetium DOTATATE whole body scans: a novel approach for evaluation of neuroendocrine tumors. *Indian J Nucl Med* 2011;**26**(3):135-8.
65. Sorschag M, Malle P, Gallowitsch HJ. Nuclear medicine in NET. *Wien Med Wochenschr* 2012;**162**(19-20):416-22.
66. Weiner RE, Thakur ML. Radiolabeled peptides in oncology: role in diagnosis and treatment. *BioDrugs* 2005;**19**(3):145-63.
67. Zandee WT, Kamp K, van Adrichem RC, Feelders RA, de Herder WW. Effect of hormone secretory syndromes on neuroendocrine tumor prognosis. *Endocr Relat Cancer* 2017;**24**(7):R261-r74.
68. Zhang J, Kulkarni HR, Lehmann C, Singh A, Baum RP. Pregnancy and delivery after PRRT without sequelae. *Clin Nucl Med* 2018;**43**(11):842-5.
69. Bodei L, Kidd MS, Singh A, et al. PRRT genomic signature in blood for prediction of (177)Lu-octreotate efficacy. *Eur J Nucl Med Mol Imaging* 2018;**45**(7):1155-69.
70. de Keizer B, van Aken MO, Feelders RA, et al. Hormonal crises following receptor radionuclide therapy with the radiolabeled somatostatin analogue [177Lu-DOTA0,Tyr3]octreotate. *Eur J Nucl Med Mol Imaging* 2008;**35**(4):749-55.

Review paper (n=61)

1. Basu S, Ranade R, Thapa P. 177Lu-DOTATATE versus 177Lu-EDTMP versus cocktail/sequential therapy in bone-confined painful metastatic disease in medullary carcinoma of the thyroid and neuroendocrine tumour: can semiquantitative comparison of 68Ga-DOTATATE and 18F-fluoride PET-CT aid in personalized treatment decision making in selecting the best therapeutic option? *Nucl Med Commun* 2016;**37**(1):100-2.
2. Bison SM, Konijnenberg MW, Melis M, et al. Peptide receptor radionuclide therapy using radiolabeled somatostatin analogs: focus on future developments. *Clin Transl Imaging* 2014;**2**(1):55-66.
3. Bodei L, Cremonesi M, Kidd M, et al. Peptide receptor radionuclide therapy for advanced neuroendocrine tumors. *Thorac Surg Clin* 2014;**24**(3):333-49.
4. Bodei L, Kidd M, Prasad V, Modlin IM. Peptide receptor radionuclide therapy of neuroendocrine tumors. *Front Horm Res* 2015;**44**:198-215.
5. Bushnell DL, Bodeker KL. Overview and current status of peptide receptor radionuclide therapy. *Surg Oncol Clin N Am* 2020;**29**(2):317-26.
6. Chin RI, Wu FS, Menda Y, Kim H. Radiopharmaceuticals for neuroendocrine tumors. *Semin Radiat Oncol* 2021;**31**(1):60-70.
7. Cives M, Strosberg J. Radionuclide therapy for neuroendocrine tumors. *Curr Oncol Rep* 2017;**19**(2):9.
8. Del Olmo-García MI, Muros MA, López-de-la-Torre M, et al. Prevention and management of hormonal crisis during theragnosis with LU-DOTA-TATE in neuroendocrine tumors. a systematic review and approach proposal. *J Clin Med* 2020;**9**(7):2203.
9. Desai H, Borges-Neto S, Wong TZ. Molecular imaging and therapy for neuroendocrine tumors. *Curr Treat Options Oncol* 2019;**20**(10):78.
10. Fazio N, Ungaro A, Spada F, et al. The role of multimodal treatment in patients with advanced lung neuroendocrine tumors. *J Thorac Dis* 2017;**9**(Suppl 15):S1501-s10.
11. Frilling A, Clift AK. Therapeutic strategies for neuroendocrine liver metastases. *Cancer* 2015;**121**(8):1172-86.
12. Granberg D, Juhlin CC, Falhammar H. Metastatic pheochromocytomas and abdominal paragangliomas. *J Clin Endocrinol Metab* 2021;**106**(5):e1937-e52.
13. Grossrubatscher E, Fanciulli G, Pes L, et al. Advances in the management of medullary thyroid carcinoma: focus on peptide receptor radionuclide therapy. *J Clin Med* 2020;**9**(11):3507.
14. Gulenchyn KY, Yao X, Asa SL, Singh S, Law C. Radionuclide therapy in neuroendocrine tumours: a systematic review. *Clin Oncol (R Coll Radiol)* 2012;**24**(4):294-308.
15. Haider M, Das S, Al-Toubah T, Pelle E, El-Haddad G, Strosberg J. Somatostatin receptor radionuclide therapy in neuroendocrine tumors. *Endocr Relat Cancer* 2021;**28**(3):R81-r93.
16. Harring TR, Nguyen NT, Goss JA, O'Mahony CA. Treatment of liver metastases in patients with neuroendocrine tumors: a comprehensive review. *Int J Hepatol* 2011;**2011**:154541.

17. Hernando J, Ros J, Arroyo A, Capdevila J. Clinical and translational challenges in thyroid cancer. *Curr Med Chem* 2020;**27**(29):4806-22.
18. Hörsch D, Schmid KW, Anlauf M, et al. Neuroendocrine tumors of the bronchopulmonary system (typical and atypical carcinoid tumors): current strategies in diagnosis and treatment. Conclusions of an expert meeting February 2011 in Weimar, Germany. *Oncol Res Treat* 2014;**37**(5):266-76.
19. Jha A, Taïeb D, Carrasquillo JA, et al. High-specific-activity-(131)I-MIBG versus (177)Lu-DOTATATE targeted radionuclide therapy for metastatic pheochromocytoma and paraganglioma. *Clin Cancer Res* 2021;**27**(11):2989-95.
20. Jungels C, Karfis I. 131I-metaiodobenzylguanidine and peptide receptor radionuclide therapy in pheochromocytoma and paraganglioma. *Curr Opin Oncol* 2021;**33**(1):33-9.
21. Kam BL, Teunissen JJ, Krenning EP, et al. Lutetium-labelled peptides for therapy of neuroendocrine tumours. *Eur J Nucl Med Mol Imaging* 2012;**39**(Suppl 1):S103-12.
22. Kesavan M, Turner JH. Myelotoxicity of peptide receptor radionuclide therapy of neuroendocrine tumors: a decade of experience. *Cancer Biother Radiopharm* 2016;**31**(6):189-98.
23. Kiesewetter B, Raderer M. My burning issues in neuroendocrine tumours (NET). *Memo* 2018;**11**(4):313-6.
24. Kim K, Kim SJ. Lu-177-based peptide receptor radionuclide therapy for advanced neuroendocrine tumors. *Nucl Med Mol Imaging* 2018;**52**(3):208-15.
25. Kim SJ, Pak K, Koo PJ, Kwak JJ, Chang S. The efficacy of (177)Lu-labelled peptide receptor radionuclide therapy in patients with neuroendocrine tumours: a meta-analysis. *Eur J Nucl Med Mol Imaging* 2015;**42**(13):1964-70.
26. Kim YI. Salvage peptide receptor radionuclide therapy in patients with progressive neuroendocrine tumors: a systematic review and meta-analysis. *Nucl Med Commun* 2021;**42**(4):451-8.
27. Kjaer A, Knigge U. Use of radioactive substances in diagnosis and treatment of neuroendocrine tumors. *Scand J Gastroenterol* 2015;**50**(6):740-7.
28. Krenning EP, Teunissen JJ, Valkema R, deHerder WW, deJong M, Kwekkeboom DJ. Molecular radiotherapy with somatostatin analogs for (neuro-)endocrine tumors. *J Endocrinol Invest* 2005;**28**(11 Suppl International):146-50.
29. La Salvia A, Espinosa-Olarte P, Riesco-Martinez MDC, Anton-Pascual B, Garcia-Carbonero R. Targeted cancer therapy: what's new in the field of neuroendocrine neoplasms? *Cancers (Basel)* 2021;**13**(7):1701.
30. Lee DY, Kim YI. Peptide receptor radionuclide therapy in patients with differentiated thyroid cancer: a meta-analysis. *Clin Nucl Med* 2020;**45**(8):604-10.
31. Lee ST, Kulkarni HR, Singh A, Baum RP. Theranostics of neuroendocrine tumors. *Visc Med* 2017;**33**(5):358-66.
32. Lo Russo G, Pusceddu S, Prinzi N, et al. Peptide receptor radionuclide therapy: focus on bronchial neuroendocrine tumors. *Tumour Biol* 2016;**37**(10):12991-3003.
33. Mak IYF, Hayes AR, Khoo B, Grossman A. Peptide receptor radionuclide therapy as a novel treatment for metastatic and invasive phaeochromocytoma and paraganglioma. *Neuroendocrinology* 2019;**109**(4):287-98.
34. Maqsood MH, Tameez Ud Din A, Khan AH. Neuroendocrine tumor therapy with lutetium-177: a literature review. *Cureus* 2019;**11**(1):e3986.
35. Minczeles NS, Hofland J, de Herder WW, Brabander T. Strategies towards improving clinical outcomes of peptide receptor radionuclide therapy. *Curr Oncol Rep* 2021;**23**(4):46.
36. Mittra ES. Neuroendocrine tumor therapy: (177)Lu-DOTATATE. *AJR Am J Roentgenol* 2018;**211**(2):278-85.
37. Ohmoto A, Morizane C. Genomic profiles and current therapeutic agents in neuroendocrine neoplasms. *Curr Drug Targets* 2020;**21**(4):389-405.
38. Pavel ME, Sers C. Women in cancer thematic review: systemic therapies in neuroendocrine tumors and novel approaches toward personalized medicine. *Endocr Relat Cancer* 2016;**23**(11):T135-t54.
39. Prasad V, Bodei L, Kidd M, Modlin IM. Whither peptide receptor radionuclide therapy for neuroendocrine tumors: an Einsteinian view of the facts and myths. *Eur J Nucl Med Mol Imaging* 2014;**41**(10):1825-30.
40. Prasad V, Brenner W, Modlin IM. How smart is peptide receptor radionuclide therapy of neuroendocrine tumors especially in the salvage setting? The clinician's perspective. *Eur J Nucl Med Mol Imaging* 2014;**41**(2):202-4.
41. Priya SR, Dravid CS, Digumarti R, Dandekar M. Targeted therapy for medullary thyroid cancer: a review. *Front Oncol* 2017;**7**:238.
42. Satapathy S, Mittal BR, Bhansali A. Peptide receptor radionuclide therapy in the management of advanced pheochromocytoma and paraganglioma: a systematic review and meta-analysis. *Clin Endocrinol (Oxf)* 2019;**91**(6):718-27.

43. Stueven AK, Kayser A, Wetz C, et al. Somatostatin analogues in the treatment of neuroendocrine tumors: past, present and future. *Int J Mol Sci* 2019;**20**(12):3049.

44. Subramaniam RM. Section Editor's Notebook: (68)Ga- and (177)Lu-DOTATATE theragnostics-personalized care and improving patient outcomes. *AJR Am J Roentgenol* 2018;**211**(2):244-5.

45. Torniai M, Scortichini L, Tronconi F, et al. Systemic treatment for lung carcinoids: from bench to bedside. *Clin Transl Med* 2019;**8**(1):22.

46. Tsoukalas N, Baxevanos P, Aravantinou-Fatorou E, et al. Advances on systemic treatment for lung neuroendocrine neoplasms. *Ann Transl Med* 2018;**6**(8):146.

47. Van Essen M, Krenning EP, De Jong M, Valkema R, Kwekkeboom DJ. Peptide receptor radionuclide therapy with radiolabelled somatostatin analogues in patients with somatostatin receptor positive tumours. *Acta Oncol* 2007;**46**(6):723-34.

48. van Essen M, Krenning EP, Kam BL, de Jong M, Valkema R, Kwekkeboom DJ. Peptide-receptor radionuclide therapy for endocrine tumors. *Nat Rev Endocrinol* 2009;**5**(7):382-93.

49. Vegt E, de Jong M, Wetzels JF, et al. Renal toxicity of radiolabeled peptides and antibody fragments: mechanisms, impact on radionuclide therapy, and strategies for prevention. *J Nucl Med* 2010;**51**(7):1049-58.

50. Wang LF, Lin L, Wang MJ, Li Y. The therapeutic efficacy of 177Lu-DOTATATE/DOTATOC in advanced neuroendocrine tumors: a meta-analysis. *Medicine (Baltimore)* 2020;**99**(10):e19304.

51. Waseem N, Aparici CM, Kunz PL. Evaluating the role of theranostics in grade 3 neuroendocrine neoplasms. *J Nucl Med* 2019;**60**(7):882-91.

52. Werner RA, Bluemel C, Allen-Auerbach MS, Higuchi T, Herrmann K. 68Gallium- and 90Yttrium-/ 177Lutetium: "theranostic twins" for diagnosis and treatment of NETs. *Ann Nucl Med* 2015;**29**(1):1-7.

53. Werner RA, Weich A, Kircher M, et al. The theranostic promise for neuroendocrine tumors in the late 2010s - Where do we stand, where do we go? *Theranostics* 2018;**8**(22):6088-100.

54. Wolin EM. Challenges in the diagnosis and management of well-differentiated neuroendocrine tumors of the lung (typical and atypical carcinoid): current status and future considerations. *Oncologist* 2015;**20**(10):1123-31.

55. Camus B, Cottereau AS, Palmieri LJ, et al. Indications of peptide receptor radionuclide therapy (PRRT) in gastroenteropancreatic and pulmonary neuroendocrine tumors: an updated review. *J Clin Med* 2021;**10**(6):1267.

56. Hörsch D, Ezziddin S, Haug A, et al. Effectiveness and side-effects of peptide receptor radionuclide therapy for neuroendocrine neoplasms in Germany: a multi-institutional registry study with prospective follow-up. *Eur J Cancer* 2016;**58**:41-51.

57. Naraev BG, Ramirez RA, Kendi AT, Halfdanarson TR. peptide receptor radionuclide therapy for patients with advanced lung carcinoids. *Clin Lung Cancer* 2019;**20**(3):e376-e92.

58. Patel M, Tena I, Jha A, Taieb D, Pacak K. somatostatin receptors and analogs in pheochromocytoma and paraganglioma: old players in a new precision medicine world. *Front Endocrinol (Lausanne)* 2021;**12**:625312.

59. Saravana-Bawan B, Bajwa A, Paterson J, McEwan AJB, McMullen TPW. Efficacy of 177Lu peptide receptor radionuclide therapy for the treatment of neuroendocrine tumors: a meta-analysis. *Clin Nucl Med* 2019;**44**(9):719-27.

60. Stolniceanu CR, Nistor I, Bilha SC, et al. Nephrotoxicity/renal failure after therapy with 90Yttrium- and 177Lutetium-radiolabeled somatostatin analogs in different types of neuroendocrine tumors: a systematic review. *Nucl Med Commun* 2020;**41**(7):601-17.

61. Taïeb D, Jha A, Treglia G, Pacak K. Molecular imaging and radionuclide therapy of pheochromocytoma and paraganglioma in the era of genomic characterization of disease subgroups. *Endocr Relat Cancer* 2019;**26**(11):R627-r52.

Non-target NET type (n=48)

1. Lutetium Lu 177 Dotatate approved by FDA. *Cancer Discov* 2018;**8**(4):Of2.

2. Arora S, Passah A, Damle NA, et al. Somatostatin receptor PET/CT features of carcinoid heart disease. *Clin Nucl Med* 2018;**43**(8):e280-e1.

3. Basu S, Parghane RV, Kamaldeep, Chakrabarty S. Peptide receptor radionuclide therapy of neuroendocrine tumors. *Semin Nucl Med* 2020;**50**(5):447-64.

4. Basu S, Ranade R, Thapa P. Correlation and discordance of tumour proliferation index and molecular imaging characteristics and their implications for treatment decisions and outcome pertaining to peptide receptor radionuclide therapy in patients with advanced neuroendocrine tumour: developing a personalized model. *Nucl Med Commun* 2015;**36**(8):766-74.

5. Bodei L, Kidd M, Baum RP, Modlin IM. PRRT: Defining the paradigm shift to achieve standardization and individualization. *J Nucl Med* 2014;**55**(11):1753-6.

6. Bodei L, Modlin IM, Luster M, et al. Myeloid neoplasms after chemotherapy and PRRT: myth and reality. *Endocr Relat Cancer* 2016;**23**(8):C1-7.

7. Brogsitter C, Zöphel K, Folprecht G, Eisenhofer G, Kotzerke J. Repeated peptide receptor radiotherapy in multiple recurrences of a metastasized neuroendocrine tumor. *Nuklearmedizin* 2017;**56**(3):N19-n21.

8. Bundschuh RA, Habacha B, Lütje S, Essler M. Therapy of patients with neuroendocrine neoplasia-evidence-based approaches and new horizons. *J Clin Med* 2019;**8**(9):1474.

9. Capdevila J, Hernando J, Perez-Hoyos S, Roman-Gonzalez A, Grande E. Meta-analysis of randomized clinical trials comparing active treatment with placebo in metastatic neuroendocrine tumors. *Oncologist* 2019;**24**(12):e1315-e20.

10. Cives M, Soares HP, Strosberg J. Will clinical heterogeneity of neuroendocrine tumors impact their management in the future? Lessons from recent trials. *Curr Opin Oncol* 2016;**28**(4):359-66.

11. Das S, Berlin J, Savona M. Pancytopenia in a patient with metastatic well-differentiated neuroendocrine tumor after peptide receptor radionuclide therapy. *JAMA Oncol* 2021;**7**(7):1060-1.

12. Davis LM, Nicou N, Martin W, et al. Timing of peptide receptor radiotargeted therapy in relation to cardiac valve surgery for carcinoid heart disease in patients with neuroendocrine metastases and cardiac syndrome. A single-centre study from a centre of excellence. *Nucl Med Commun* 2020;**41**(6):575-81.

13. de Jong M, Kwekkeboom D, Valkema R, Krenning EP. Radiolabelled peptides for tumour therapy: current status and future directions. Plenary lecture at the EANM 2002. *Eur J Nucl Med Mol Imaging* 2003;**30**(3):463-9.

14. de Visser M, Verwijnen SM, de Jong M. Update: improvement strategies for peptide receptor scintigraphy and radionuclide therapy. *Cancer Biother Radiopharm* 2008;**23**(2):137-57.

15. Ebbers SC, Braat A, Moelker A, Stokkel MPM, Lam M, Barentsz MW. Intra-arterial versus standard intravenous administration of lutetium-177-DOTA-octreotate in patients with NET liver metastases: study protocol for a multicenter, randomized controlled trial (LUTIA trial). *Trials* 2020;**21**(1):141.

16. Feola T, Centello R, Sesti F, et al. Neuroendocrine carcinomas with atypical proliferation index and clinical behavior: a systematic review. *Cancers (Basel)* 2021;**13**(6):1247.

17. Gerasimou G, Moralidis E, Gotzamani-Psarrakou A. Somatostatin receptor imaging with (111)In-pentetreotide in gastro-intestinal tract and lung neuroendocrine tumors-Impact on targeted treatment. *Hell J Nucl Med* 2010;**13**(2):158-62.

18. Gezer E, Çetinarslan B, Cantürk Z, Tarkun İ, Sözen M, Selek A. Metastatic MEN1 syndrome treated with lutetium-177 – a case report. *Eur Endocrinol* 2019;**15**(2):92-4.

19. Hendifar AE, Delpassand ES, Kittleson MM, Tuli R. Cardiac toxicity in a patient receiving peptide receptor radionuclide therapy. *Pancreas* 2018;**47**(8):e55-e6.

20. Jafari E, Ahmadzadehfar H, Bagheri D, Amini A, Assadi M. Assessment of early oxidative stress following the use of radiotheranostics agents 177Lu-PSMA for prostate cancer and 177Lu-DOTATATE for neuroendocrine tumors; radioprotective effect of vitamin C. *Nucl Med Commun* 2021;**42**(3):325-31.

21. Jois B, Asopa R, Basu S. Somatostatin receptor imaging in non-(131)I-avid metastatic differentiated thyroid carcinoma for determining the feasibility of peptide receptor radionuclide therapy with (177)Lu-DOTATATE: low fraction of patients suitable for peptide receptor radionuclide therapy and evidence of chromogranin A level-positive neuroendocrine differentiation. *Clin Nucl Med* 2014;**39**(6):505-10.

22. Khasraw M, Gill A, Harrington T, Pavlakis N, Modlin I. Management of advanced neuroendocrine tumors with hepatic metastasis. *J Clin Gastroenterol* 2009;**43**(9):838-47.

23. Kwekkeboom DJ, Krenning EP. Peptide receptor radionuclide therapy in the treatment of neuroendocrine tumors. *Hematol Oncol Clin North Am* 2016;**30**(1):179-91.

24. Kwekkeboom DJ, Krenning EP, Lebtahi R, et al. ENETS consensus guidelines for the standards of care in neuroendocrine tumors: peptide receptor radionuclide therapy with radiolabeled somatostatin analogs. *Neuroendocrinology* 2009;**90**(2):220-6.

25. Lawal I, Louw L, Warwick J, et al. The College of Nuclear Physicians of South Africa practice guidelines on peptide receptor radionuclide therapy in neuroendocrine tumours. *S Afr J Surg* 2018;**56**(3):55-64.

26. Levine R, Krenning EP. Clinical history of the theranostic radionuclide approach to neuroendocrine tumors and other types of cancer: historical review based on an interview of Eric P. Krenning by Rachel Levine. *J Nucl Med* 2017;**58**(Suppl 2):3s-9s.

27. Liu AJ, Halfdanarson TR, Sonbol MB. Currarino syndrome: a rare condition with potential connection to neuroendocrine tumors. *Pancreas* 2020;**49**(8):1104-8.

28. Liu T, Liao J, Dang J, Li G. Treatments for patients with advanced neuroendocrine tumors: a network meta-analysis. *Ther Adv Med Oncol* 2019;**11**:1758835919853673.

29. Luster M, Pfestroff A, Verburg FA. Recent advances in nuclear medicine in endocrine oncology. *Curr Opin Oncol* 2017;**29**(1):1-6.

30. Makis W, McCann K, Bryanton M, McEwan AJ. Cardiac metastases of neuroendocrine tumors treated with 177Lu DOTATATE peptide receptor radionuclide therapy or 131I-MIBG therapy. *Clin Nucl Med* 2015;**40**(12):962-4.

31. Makis W, McCann K, Buteau FA, McEwan AJ. complete resolution of neuroendocrine tumor soft tissue metastases after 177Lu DOTATATE PRRT induction and maintenance therapy. *Clin Nucl Med* 2015;**40**(8):663-6.

32. Negro A, Rossi GM, Nicoli D, et al. peptide receptor radionuclide therapy-induced Gitelman-like syndrome. *Am J Kidney Dis* 2017;**70**(5):725-8.

33. Pach D, Sowa-Staszczak A, Kunikowska J, et al. Repeated cycles of peptide receptor radionuclide therapy (PRRT) – results and side-effects of the radioisotope 90Y-DOTA TATE, 177Lu-DOTA TATE or 90Y/177Lu-DOTA TATE therapy in patients with disseminated NET. *Radiother Oncol* 2012;**102**(1):45-50.

34. Paganelli G, Sansovini M, Scarpi E. Reply to: Predicting the outcome of peptide receptor radionuclide therapy in neuroendocrine tumors: the importance of dual-tracer imaging. *Eur J Nucl Med Mol Imaging* 2017;**44**(10):1777-8.

35. Piccin A, Grana CM, Negri G, Pusceddu I, Paganelli G, Cortelazzo S. Secondary acute myeloid leukaemia after peptide receptor radionuclide therapy. *Ann Hematol* 2012;**91**(2):299-300.

36. Salner AL, Blankenship B, Dunnack H, Niemann C, Bertsch H. Lutetium Lu-177 Dotatate flare reaction. *Adv Radiat Oncol* 2021;**6**(1):100623.

37. Sierra ML, Agazzi A, Bodei L, et al. Lymphocytic toxicity in patients after peptide-receptor radionuclide therapy (PRRT) with 177Lu-DOTATATE and 90Y-DOTATOC. *Cancer Biother Radiopharm* 2009;**24**(6):659-65.

38. Smit Duijzentkunst DA, Kwekkeboom DJ, Bodei L. Somatostatin receptor 2-targeting compounds. *J Nucl Med* 2017;**58**(Suppl 2):54s-60s.

39. Sowa-Staszczak A, Hubalewska-Dydejczyk A, Tomaszuk M. PRRT as neoadjuvant treatment in NET. *Recent Results Cancer Res* 2013;**194**:479-85.

40. Strosberg J, Leeuwenkamp O, Siddiqui MK. Peptide receptor radiotherapy re-treatment in patients with progressive neuroendocrine tumors: a systematic review and meta-analysis. *Cancer Treat Rev* 2021;**93**:102141.

41. Teunissen JJ, Kwekkeboom DJ, Kooij PP, Bakker WH, Krenning EP. Peptide receptor radionuclide therapy for non-radioiodine-avid differentiated thyroid carcinoma. *J Nucl Med* 2005;**46**(Suppl 1):107s-14s.

42. Turner JH. Recent advances in theranostics and challenges for the future. *Br J Radiol* 2018;**91**(1091):20170893.

43. Vallathol DH, Ali AZ, Alapati N, Manasa N, Vasanth P, Digumarti R. Lutetium 177 dotatate induced acute myeloid leukemia. *Leuk Lymphoma* 2020;**61**(13):3263-5.

44. Walter T, Brixi-Benmansour H, Lombard-Bohas C, Cadiot G. New treatment strategies in advanced neuroendocrine tumours. *Dig Liver Dis* 2012;**44**(2):95-105.

45. Wang L, Tang K, Zhang Q, et al. Somatostatin receptor-based molecular imaging and therapy for neuroendocrine tumors. *Biomed Res Int* 2013;**2013**:102819.

46. Wu Q, Chen B, Yan G, Yang Z, Xiong L, He J. A systematic review and meta-analysis of gastrointestinal events associated with nonoperative therapies for neuroendocrine tumors. *Onco Targets Ther* 2018;**11**:7655-68.

47. Zanotti-Fregonara P, Morgat C, Champion C, Hindié E. Predicting the outcome of peptide receptor radionuclide therapy in neuroendocrine tumors: the importance of dual-tracer imaging. *Eur J Nucl Med Mol Imaging* 2017;**44**(6):1095-6.

48. Malhotra G, Asopa RV. Metastatic mediastinal carcinoid presenting as superior mediastinal syndrome and treated with peptide receptor radionuclide therapy. *Clin Nucl Med* 2014;**39**(1):e89-92.

Non-separable treatment (n=29)

1. Aalbersberg EA, Huizing DMV, Walraven I, et al. Parameters to predict progression-free and overall survival after peptide receptor radionuclide therapy: a multivariate analysis in 782 patients. *J Nucl Med* 2019;**60**(9):1259-65.

2. Abou Jokh Casas E, Pubul Núñez V, Anido-Herranz U, et al. Evaluation of (177)Lu-Dotatate treatment in patients with metastatic neuroendocrine tumors and prognostic factors. *World J Gastroenterol* 2020;**26**(13):1513-24.

3. Baum RP, Kulkarni HR, Singh A, et al. Results and adverse events of personalized peptide receptor radionuclide therapy with (90)Yttrium and (177)Lutetium in 1048 patients with neuroendocrine neoplasms. *Oncotarget* 2018;**9**(24):16932-50.

4. Bodei L, Ćwikla JB, Kidd M, Modlin IM. The role of peptide receptor radionuclide therapy in advanced/metastatic thoracic neuroendocrine tumors. *J Thorac Dis* 2017;**9**(Suppl 15):S1511-s23.

5. Bongiovanni A, Recine F, Foca F, et al. Metastatic neuroendocrine neoplasia treatments in patients over 70 years of age. *Endocr Connect* 2018;**7**(12):1535-41.

6. Braat A, Ahmadzadehfar H, Kappadath SC, et al. Radioembolization with (90)Y resin microspheres of neuroendocrine liver metastases after initial peptide receptor radionuclide therapy. *Cardiovasc Intervent Radiol* 2020;**43**(2):246-53.

7. Brans B, Mottaghy FM, Kessels A. 90Y/177Lu-DOTATATE therapy: survival of the fittest? *Eur J Nucl Med Mol Imaging* 2011;**38**(10):1785-7.

8. Budiawan H, Salavati A, Kulkarni HR, Baum RP. Peptide receptor radionuclide therapy of treatment-refractory metastatic thyroid cancer using (90)Yttrium and (177)Lutetium labeled somatostatin analogs: toxicity, response and survival analysis. *Am J Nucl Med Mol Imaging* 2013;**4**(1):39-52.

9. Bushnell D. Treatment of metastatic carcinoid tumors with radiolabeled biologic molecules. *J Natl Compr Canc Netw* 2009;**7**(7):760-4.

10. De Filpo G, Maggi M, Mannelli M, Canu L. Management and outcome of metastatic pheochromocytomas/paragangliomas: an overview. *J Endocrinol Invest* 2021;**44**(1):15-25.

11. Gabriel M, Nilica B, Kaiser B, Virgolini IJ. Twelve-year follow-up after peptide receptor radionuclide therapy. *J Nucl Med* 2019;**60**(4):524-9.

12. Graf J, Pape UF, Jann H, et al. Prognostic significance of somatostatin receptor heterogeneity in progressive neuroendocrine tumor treated with Lu-177 DOTATOC or Lu-177 DOTATATE. *Eur J Nucl Med Mol Imaging* 2020;**47**(4):881-94.

13. Hamiditabar M, Ali M, Roys J, et al. Peptide receptor radionuclide therapy with 177Lu-octreotate in patients with somatostatin receptor expressing neuroendocrine tumors: six years' assessment. *Clin Nucl Med* 2017;**42**(6):436-43.

14. Kamieniarz L, Armeni E, O'Mahony LF, et al. Orbital metastases from neuroendocrine neoplasms: clinical implications and outcomes. *Endocrine* 2020;**67**(2):485-93.

15. Katona BW, Roccaro GA, Soulen MC, et al. Efficacy of peptide receptor radionuclide therapy in a United States-based cohort of metastatic neuroendocrine tumor patients: single-institution retrospective analysis. *Pancreas* 2017;**46**(9):1121-6.

16. Kong G, Grozinsky-Glasberg S, Hofman MS, et al. Efficacy of peptide receptor radionuclide therapy for functional metastatic paraganglioma and pheochromocytoma. *J Clin Endocrinol Metab* 2017;**102**(9):3278-87.

17. Kulkarni HR, Prasad V, Schuchardt C, Baum RP. Is there a correlation between peptide receptor radionuclide therapy-associated hematological toxicity and spleen dose? *Recent Results Cancer Res* 2013;**194**:561-6.

18. Lim LE, Chan DL, Thomas D, et al. Australian experience of peptide receptor radionuclide therapy in lung neuroendocrine tumours. *Oncotarget* 2020;**11**(27):2636-46.

19. Nastos K, Cheung VTF, Toumpanakis C, et al. Peptide receptor radionuclide treatment and (131)I-MIBG in the management of patients with metastatic/progressive phaeochromocytomas and paragangliomas. *J Surg Oncol* 2017;**115**(4):425-34.

20. Panzuto F, Rinzivillo M, Fazio N, et al. Real-world study of everolimus in advanced progressive neuroendocrine tumors. *Oncologist* 2014;**19**(9):966-74.

21. Puranik AD, Kulkarni HR, Singh A, Baum RP. Peptide receptor radionuclide therapy with (90)Y/ (177)Lu-labelled peptides for inoperable head and neck paragangliomas (glomus tumours). *Eur J Nucl Med Mol Imaging* 2015;**42**(8):1223-30.

22. Ramirez RA, Chauhan A, Gimenez J, Thomas KEH, Kokodis I, Voros BA. Management of pulmonary neuroendocrine tumors. *Rev Endocr Metab Disord* 2017;**18**(4):433-42.

23. Salavati A, Puranik A, Kulkarni HR, Budiawan H, Baum RP. Peptide receptor radionuclide therapy (PRRT) of medullary and nonmedullary thyroid cancer using radiolabeled somatostatin analogues. *Semin Nucl Med* 2016;**46**(3):215-24.

24. Sowa-Staszczak A, Stefanska A, Chrapczynski P, Trofimiuk-Müldner M, Szura M, Hubalewska-Dydejczyk A. Does combination of "cold" and "hot" somatostatin analogs prolong survival of patients with neuroendocrine neoplasms? *Endocr J* 2017;**64**(2):171-7.

25. Srirajaskanthan R, Toubanakis C, Dusmet M, Caplin ME. A review of thymic tumours. *Lung Cancer* 2008;**60**(1):4-13.
26. Zhang J, Liu Q, Singh A, Schuchardt C, Kulkarni HR, Baum RP. Prognostic value of (18)F-FDG PET/CT in a large cohort of patients with advanced metastatic neuroendocrine neoplasms treated with peptide receptor radionuclide therapy. *J Nucl Med* 2020;**61**(11):1560-9.
27. Kaemmerer D, Twrznik M, Kulkarni HR, et al. Prior resection of the primary tumor prolongs survival after peptide receptor radionuclide therapy of advanced neuroendocrine neoplasms. *Ann Surg* 2021;**274**(1):e45-e53.
28. Sharma N, Naraev BG, Engelman EG, et al. Peptide receptor radionuclide therapy outcomes in a north american cohort with metastatic well-differentiated neuroendocrine tumors. *Pancreas* 2017;**46**(2):151-6.
29. Zacho MD, Iversen P, Villadsen GE, et al. Clinical efficacy of first and second series of peptide receptor radionuclide therapy in patients with neuroendocrine neoplasm: a cohort study. *Scand J Gastroenterol* 2021;**56**(3):289-97.

Case report or only individual data reported (n=28)

1. Acar E, Çapa Kaya G, Durak H. Hepatopulmonary shunt reduction with 177Lu-DOTATATE therapy. *Clin Nucl Med* 2016;**41**(11):866-71.
2. Asbach S, Schluermann F, Ruf J, Bode C, Lang C. Apparent pacemaker dysfunction during peptide receptor radionuclide therapy for neuroendocrine tumor. *Clin Case Rep* 2018;**6**(1):197-9.
3. Balter H, Victoria T, Mariella T, et al. ^{177}Lu-labeled agents for neuroendocrine tumor therapy and bone pain palliation in Uruguay. *Curr Radiopharm* 2016;**9**(1):85-93.
4. Basu S, Ranade R, Thapa P. Metastatic neuroendocrine tumor with extensive bone marrow involvement at diagnosis: evaluation of response and hematological toxicity profile of PRRT with (177)Lu-DOTATATE. *World J Nucl Med* 2016;**15**(1):38-43.
5. Bodei L, Cremonesi M, Ferrari M, et al. Long-term evaluation of renal toxicity after peptide receptor radionuclide therapy with 90Y-DOTATOC and 177Lu-DOTATATE: the role of associated risk factors. *Eur J Nucl Med Mol Imaging* 2008;**35**(10):1847-56.
6. Cecchin D, Schiavi F, Fanti S, et al. Peptide receptor radionuclide therapy in a case of multiple spinal canal and cranial paragangliomas. *J Clin Oncol* 2011;**29**(7):e171-4.
7. Chiapponi C, Lürssen N, Cremer B, et al. Peptide receptor radionuclide therapy as a two-step strategy for initially unresectable liver disease from neuroendocrine tumors: a single-center experience. *Endocrine* 2020;**70**(1):187-93.
8. Cottereau AS, Bricaire L, Arrondeau J, et al. Prolonged response to (177)Lu-DOTATATE therapy of a bone marrow infiltration in a refractory thymic neuro endocrine tumor. *Invest New Drugs* 2020;**38**(4):1196-9.
9. Donato S, Simões H, Pinto AT, B MC, Leite V. SDHx-related pheochromocytoma/paraganglioma – genetic, clinical, and treatment outcomes in a series of 30 patients from a single center. *Endocrine* 2019;**65**(2):408-15.
10. Escala Cornejo RA, García-Talavera P, Navarro Martin M, et al. Large cell neuroendocrine carcinoma of the lung with atypical evolution and a remarkable response to lutetium Lu 177 dotatate. *Ann Nucl Med* 2018;**32**(8):568-72.
11. Estêvão R, Duarte H, Lopes F, Fernandes J, Monteiro E. Peptide receptor radionuclide therapy in head and neck paragangliomas – report of 14 cases. *Rev Laryngol Otol Rhinol (Bord)* 2015;**136**(4):155-8.
12. Gubbi S, Al-Jundi M, Del Rivero J, et al. Case Report: Primary hypothyroidism associated with lutetium 177-DOTATATE therapy for metastatic paraganglioma. *Front Endocrinol (Lausanne)* 2020;**11**:587065.
13. Gupta SK, Singla S, Karunanithi S, Damle N, Bal C. Peptide receptor radionuclide therapy with (177)Lu DOTATATE in a case of recurrent carotid body paraganglioma with spinal metastases. *Clin Nucl Med* 2014;**39**(5):440-1.
14. Huang K, Brenner W, Prasad V. Tumor lysis syndrome: a rare but serious complication of radioligand therapies. *J Nucl Med* 2019;**60**(6):752-5.
15. Lapa C, Hänscheid H, Wild V, et al. Somatostatin receptor expression in small cell lung cancer as a prognostic marker and a target for peptide receptor radionuclide therapy. *Oncotarget* 2016;**7**(15):20033-40.
16. Leijon H, Remes S, Hagström J, et al. Variable somatostatin receptor subtype expression in 151 primary pheochromocytomas and paragangliomas. *Hum Pathol* 2019;**86**:66-75.
17. Makis W, McCann K, McEwan AJ. Medullary thyroid carcinoma (MTC) treated with 177Lu-DOTATATE PRRT: a report of two cases. *Clin Nucl Med* 2015;**40**(5):408-12.
18. Makis W, McCann K, McEwan AJ. The challenges of treating paraganglioma patients with (177)Lu-DOTATATE PRRT: catecholamine crises, tumor lysis syndrome and the need for modification of treatment protocols. *Nucl Med Mol Imaging* 2015;**49**(3):223-30.

19. Makis W, McCann K, McEwan AJ. Thymoma treated with 177Lu DOTATATE induction and maintenance PRRT. *Clin Nucl Med* 2015;**40**(5):e278-81.

20. Muros MA, Varsavsky M, Iglesias Rozas P, et al. Outcome of treating advanced neuroendocrine tumours with radiolabelled somatostatin analogues. *Clin Transl Oncol* 2009;**11**(1):48-53.

21. Naik C, Basu S. Peptide receptor radionuclide therapy with (177)Lu-DOTATATE for metastatic neuroendocrine tumor occurring in association with multiple endocrine neoplasia type 1 and Cushing's syndrome. *World J Nucl Med* 2017;**16**(2):126-32.

22. Naik C, Basu S. (177)Lu-DOTATATE peptide receptor radionuclide therapy in patients with borderline low and discordant renal parameters: treatment feasibility assessment by sequential estimation of triple parameters and filtration fraction. *World J Nucl Med* 2018;**17**(1):12-20.

23. Skoura E, Priftakis D, Novruzov F, et al. The impact of Ga-68 DOTATATE PET/CT imaging on management of patients with paragangliomas. *Nucl Med Commun* 2020;**41**(2):169-74.

24. Soydal Ç, Peker A, Özkan E, Küçük Ö N, Kir MK. The role of baseline Ga-68 DOTATATE positron emission tomography/computed tomography in the prediction of response to fixed-dose peptide receptor radionuclide therapy with Lu-177 DOTATATE. *Turk J Med Sci* 2016;**46**(2):409-13.

25. Thapa P, Parghane R, Basu S. (177)Lu-DOTATATE peptide receptor radionuclide therapy in metastatic or advanced and inoperable primary neuroendocrine tumors of rare sites. *World J Nucl Med* 2017;**16**(3):223-8.

26. Trêpa M, Silveira I, Amaral C, Luz A. Innovative approach to a functional mediastinal paraganglioma with anomalous coronary supply: a case report. *Eur Heart J Case Rep* 2020;**4**(3):1-6.

27. Wolf KI, Jha A, van Berkel A, et al. Eruption of metastatic paraganglioma after successful therapy with (177)Lu/(90)Y-DOTATOC and (177)Lu-DOTATATE. *Nucl Med Mol Imaging* 2019;**53**(3):223-30.

28. Zovato S, Kumanova A, Demattè S, et al. Peptide receptor radionuclide therapy (PRRT) with 177Lu-DOTATATE in individuals with neck or mediastinal paraganglioma (PGL). *Horm Metab Res* 2012;**44**(5):411-4.

Not treated with [^{177}Lu]Lu-DOTA-TATE (n=27)

1. Apostolidis L, Dal Buono A, Merola E, et al. Multicenter analysis of treatment outcomes for systemic therapy in well differentiated grade 3 neuroendocrine tumors (NET G3). *Cancers (Basel)* 2021;**13**(8):1936.

2. Arveschoug AK, Bekker AC, Iversen P, Bluhme H, Villadsen GE, Staanum PF. Extravasation of [(177)Lu]Lu-DOTATOC: case report and discussion. *EJNMMI Res* 2020;**10**(1):68.

3. Baum RP, Kluge AW, Kulkarni H, et al. [(177)Lu-DOTA](0)-D-Phe(1)-Tyr(3)-Octreotide ((177)Lu-DOTATOC) for peptide receptor radiotherapy in patients with advanced neuroendocrine tumours: a phase-II study. *Theranostics* 2016;**6**(4):501-10.

4. Baum RP, Zhang J, Schuchardt C, Mueller D, Maecke H. First-in-human study of novel SSTR antagonist (177)Lu-DOTA-LM3 for peptide receptor radionuclide therapy in patients with metastatic neuroendocrine neoplasms: dosimetry, safety and efficacy. *J Nucl Med* 2021;**62**(11):1571-81.

5. Breeman WA, de Jong M, Kwekkeboom DJ, et al. Somatostatin receptor-mediated imaging and therapy: basic science, current knowledge, limitations and future perspectives. *Eur J Nucl Med* 2001;**28**(9):1421-9.

6. Brunner P, Jörg AC, Glatz K, et al. The prognostic and predictive value of sstr(2)-immunohistochemistry and sstr(2)-targeted imaging in neuroendocrine tumors. *Eur J Nucl Med Mol Imaging* 2017;**44**(3):468-75.

7. Burkett BJ, Dundar A, Young JR, et al. How we do it: a multidisciplinary approach to (177)Lu DOTATATE peptide receptor radionuclide therapy. *Radiology* 2021;**298**(2):261-74.

8. Bussink J, Span PN. γ-H2AX foci in peripheral blood lymphocytes to quantify radiation-induced DNA damage after 177Lu-DOTA-octreotate peptide receptor radionuclide therapy. *J Nucl Med* 2015;**56**(4):501-2.

9. Ferolla P. Medical treatment of advanced thoracic neuroendocrine tumors. *Thorac Surg Clin* 2014;**24**(3):351-5.

10. Filipović A, Vucković L, Pejakov L. Paraganglioma of the thyroid gland: a case report. *Vojnosanit Pregl* 2014;**71**(9):875-8.

11. Forrer F, Riedweg I, Maecke HR, Mueller-Brand J. Radiolabeled DOTATOC in patients with advanced paraganglioma and pheochromocytoma. *Q J Nucl Med Mol Imaging* 2008;**52**(4):334-40.

12. Hammel P, Hentic O, Neuzillet C, Faivre S, Raymond E, Ruszniewski P. New treatment options with cytotoxic agents in neuroendocrine tumours. *Target Oncol* 2012;**7**(3):169-72.

13. Komoda S, Komoda T, Knosalla C, et al. A giant neuroendocrine tumor of the thymus gland causing superior vena cava syndrome. *Gen Thorac Cardiovasc Surg* 2012;**60**(12):863-7.

14. Makis W, McCann K, McEwan AJ. Orbital metastases of neuroendocrine tumors treated with 177Lu-DOTATATE PRRT or 131I-MIBG therapies. *Clin Nucl Med* 2016;**41**(2):137-41.

15. Pfeifer AK, Gregersen T, Grønbæk H, et al. Peptide receptor radionuclide therapy with Y-DOTATOC and (177)Lu-DOTATOC in advanced neuroendocrine tumors: results from a Danish cohort treated in Switzerland. *Neuroendocrinology* 2011;**93**(3):189-96.

16. Poeppel TD, Yuece A, Boy C, et al. Novel SDHD gene mutation (H102R) in a patient with metastatic cervical paraganglioma effectively treated by peptide receptor radionuclide therapy. *J Clin Oncol* 2011;**29**(33):e812-5.

17. Ruhwedel T, Rogasch JMM, Huang K, et al. The prognostic value of the De Ritis Ratio for progression-free survival in patients with NET undergoing [(177)Lu]Lu-DOTATOC-PRRT: a retrospective analysis. *Cancers (Basel)* 2021;**13**(4):635.

18. Sowa-Staszczak A, Chrzan R, Pach D, et al. Are RECIST criteria sufficient to assess response to therapy in neuroendocrine tumors? *Clin Imaging* 2012;**36**(4):360-4.

19. Sowa-Staszczak A, Pach D, Chrzan R, et al. Peptide receptor radionuclide therapy as a potential tool for neoadjuvant therapy in patients with inoperable neuroendocrine tumours (NETs). *Eur J Nucl Med Mol Imaging* 2011;**38**(9):1669-74.

20. Swiętaszczyk C, Prasad V, Baum RP. Intense 18F-fluoride accumulation in liver metastases from a neuroendocrine tumor after peptide receptor radionuclide therapy. *Clin Nucl Med* 2012;**37**(4):e82-3.

21. Teunissen JJ, Kwekkeboom DJ, Valkema R, Krenning EP. Nuclear medicine techniques for the imaging and treatment of neuroendocrine tumours. *Endocr Relat Cancer* 2011;**18**(Suppl 1): S27-51.

22. Tuncel M, Kılıçkap S, Süslü N. Clinical impact of (68)Ga-DOTATATE PET-CT imaging in patients with medullary thyroid cancer. *Ann Nucl Med* 2020;**34**(9):663-74.

23. Umlauft M, Radojewski P, Spanjol PM, et al. Diabetes mellitus and its effects on all-cause mortality after radiopeptide therapy for neuroendocrine tumors. *J Nucl Med* 2017;**58**(1):97-102.

24. Van Binnebeek S, Baete K, Terwinghe C, et al. Significant impact of transient deterioration of renal function on dosimetry in PRRT. *Ann Nucl Med* 2013;**27**(1):74-7.

25. Yadav SK, Jha CK, Patil S, Datta D, Mishra A, Pradhan PK. Lutetium therapy-induced carcinoid crisis: a case report and review of literature. *J Cancer Res Ther* 2020;**16**(Suppl):S206-s8.

26. Zöphel K, Strumpf A, Wunderlich G, Oehme L, Eisenhofer G, Kotzerke J. Cure of neuroendocrine carcinoma by peptide receptor radionuclide therapy. *Clin Nucl Med* 2008;**33**(10):690-1.

27. Wild D, Fani M, Fischer R, et al. Comparison of somatostatin receptor agonist and antagonist for peptide receptor radionuclide therapy: a pilot study. *J Nucl Med* 2014;**55**(8):1248-52.

Did not report specific NET type (n=21)

1. Lutetium Lu 177 Dotatate. Drugs and Lactation Database (LactMed). Bethesda (MD): National Library of Medicine (US); 2006.

2. Arora S, Passah A, Damle NA, et al. Clinical issues related to fluid management during (177)Lu-peptide receptor radionuclide therapy in metastatic neuroendocrine tumors with carcinoid heart disease. *Indian J Nucl Med* 2018;**33**(4):359-61.

3. Bergsma H, Konijnenberg MW, Kam BL, et al. Subacute haematotoxicity after PRRT with (177)Lu-DOTA-octreotate: prognostic factors, incidence and course. *Eur J Nucl Med Mol Imaging* 2016;**43**(3):453-63.

4. Dannoon SF, Alenezi SA, Elgazzar AH. The efficacy of the available peptide receptor radionuclide therapy for neuroendocrine tumors: a meta-analysis. *Nucl Med Commun* 2017;**38**(12):1085-93.

5. De Jong M, Valkema R, Jamar F, et al. Somatostatin receptor-targeted radionuclide therapy of tumors: preclinical and clinical findings. *Semin Nucl Med* 2002;**32**(2):133-40.

6. Jafari E, Amini AL, Ahmadzadehfar H, Bagheri D, Assadi M. Cardiotoxicity and cardiac monitoring following the use of radiotheranostics agents including 177Lu-PSMA for prostate cancer and 177Lu-DOTATATE for neuroendocrine tumors. *Nuklearmedizin* 2021;**60**(2):99-105.

7. Kulkarni HR, Schuchardt C, Baum RP. Peptide receptor radionuclide therapy with (177)Lu labeled somatostatin analogs DOTATATE and DOTATOC: contrasting renal dosimetry in the same patient. *Recent Results Cancer Res* 2013;**194**:551-9.

8. Mujica-Mota R, Varley-Campbell J, Tikhonova I, et al. Everolimus, lutetium-177 DOTATATE and sunitinib for advanced, unresectable or metastatic neuroendocrine tumours with disease progression: a systematic review and cost-effectiveness analysis. *Health Technol Assess* 2018;**22**(49):1-326.
9. Narayanan S, Kunz PL. Role of somatostatin analogues in the treatment of neuroendocrine tumors. *Hematol Oncol Clin North Am* 2016;**30**(1):163-77.
10. Nicolas G, Giovacchini G, Müller-Brand J, Forrer F. Targeted radiotherapy with radiolabeled somatostatin analogs. *Endocrinol Metab Clin North Am* 2011;**40**(1):187-204, ix-x.
11. Öberg KE. Management of neuroendocrine tumors: current and future therapies. *Expert Rev Endocrinol Metab* 2011;**6**(1):49-62.
12. Oberg KE. The management of neuroendocrine tumours: current and future medical therapy options. *Clin Oncol (R Coll Radiol)* 2012;**24**(4):282-93.
13. Sandström M, Garske-Román U, Granberg D, et al. Individualized dosimetry of kidney and bone marrow in patients undergoing 177Lu-DOTA-octreotate treatment. *J Nucl Med* 2013;**54**(1):33-41.
14. Severi S, Nanni O, Bodei L, et al. Role of 18FDG PET/CT in patients treated with 177Lu-DOTATATE for advanced differentiated neuroendocrine tumours. *Eur J Nucl Med Mol Imaging* 2013;**40**(6):881-8.
15. Tapia Rico G, Li M, Pavlakis N, Cehic G, Price TJ. Prevention and management of carcinoid crises in patients with high-risk neuroendocrine tumours undergoing peptide receptor radionuclide therapy (PRRT): Literature review and case series from two Australian tertiary medical institutions. *Cancer Treat Rev* 2018;**66**:1-6.
16. Uri I, Avniel-Polak S, Gross DJ, Grozinsky-Glasberg S. Update in the therapy of advanced neuroendocrine tumors. *Curr Treat Options Oncol* 2017;**18**(12):72.
17. Valkema R, Pauwels SA, Kvols LK, et al. Long-term follow-up of renal function after peptide receptor radiation therapy with (90)Y-DOTA(0),Tyr(3)-octreotide and (177)Lu-DOTA(0), Tyr(3)-octreotate. *J Nucl Med* 2005;**46**(Suppl 1):83s-91s.
18. Vija L, Dierickx L, Courbon F. Receptor radionuclide targeting for neuroendocrine tumors (NET) diagnostic and therapy. *Ann Endocrinol (Paris)* 2019;**80**(3):166-71.
19. Virgolini I. Peptide receptor radionuclide therapy (PRRT): clinical significance of re-treatment? *Eur J Nucl Med Mol Imaging* 2015;**42**(13):1949-54.
20. Wee CE, Dundar A, Eiring RA, et al. Bowel obstruction as a complication of peptide receptor radionuclide therapy (PRRT). *J Nucl Med* 2021;**62**(9):1320.
21. Zhang J, Wang H, Jacobson O, et al. Safety, pharmacokinetics, and dosimetry of a long-acting radiolabeled somatostatin analog (177)Lu-DOTA-EB-TATE in patients with advanced metastatic neuroendocrine tumors. *J Nucl Med* 2018;**59**(11):1699-705.

[^{177}Lu]Lu-DOTA-TATE used in tandem/combination with another anticancer agent (n=9)

1. Ashwathanarayana AG, Biswal CK, Sood A, Parihar AS, Kapoor R, Mittal BR. Imaging-guided use of combined (177)Lu-DOTATATE and capecitabine therapy in metastatic mediastinal paraganglioma. *J Nucl Med Technol* 2017;**45**(4):314-6.
2. Goncalves I, Burbury K, Michael M, et al. Characteristics and outcomes of therapy-related myeloid neoplasms after peptide receptor radionuclide/chemoradionuclide therapy (PRRT/PRCRT) for metastatic neuroendocrine neoplasia: a single-institution series. *Eur J Nucl Med Mol Imaging* 2019;**46**(9):1902-10.
3. Kwekkeboom DJ. Therapy: Neuroendocrine cancer – are two radionuclides better than one? *Nat Rev Endocrinol* 2012;**8**(6):326-8.
4. Satapathy S, Mittal BR, Sood A, Sood A, Kapoor R, Gupta R. Peptide receptor radionuclide therapy as first-line systemic treatment in advanced inoperable/metastatic neuroendocrine tumors. *Clin Nucl Med* 2020;**45**(9):e393-e9.
5. Sood A, Singh H, Sood A, Basher RK, Mittal BR. Incidentally detected thyroid follicular neoplasm on somatostatin receptor imaging and post-therapy Scan. *Indian J Nucl Med* 2017;**32**(3):224-6.
6. Thang SP, Lung MS, Kong G, et al. Peptide receptor radionuclide therapy (PRRT) in European Neuroendocrine Tumour Society (ENETS) grade 3 (G3) neuroendocrine neoplasia (NEN) - a single-institution retrospective analysis. *Eur J Nucl Med Mol Imaging* 2018;**45**(2):262-77.
7. Yordanova A, Ahrens H, Feldmann G, et al. Peptide receptor radionuclide therapy combined with chemotherapy in patients with neuroendocrine tumors. *Clin Nucl Med* 2019;**44**(5):e329-e35.

8. Denoyer D, Lobachevsky P, Jackson P, Thompson M, Martin OA, Hicks RJ. Analysis of 177Lu-DOTA-octreotate therapy-induced DNA damage in peripheral blood lymphocytes of patients with neuroendocrine tumors. *J Nucl Med* 2015;**56**(4):505-11.
9. Jha A, de Luna K, Balili CA, et al. Clinical, diagnostic, and treatment characteristics of SDHA-related metastatic pheochromocytoma and paraganglioma. *Front Oncol* 2019;**9**:53.

PRRT product not specified (n=3)

1. Faggiano A, Di Maio S, Mocerino C, et al. Therapeutic sequences in patients with grade 1-2 neuroendocrine tumors (NET): an observational multicenter study from the ELIOS group. *Endocrine* 2019;**66**(2):417-24.
2. Mayer K, Kiry S, Yordanova A, et al. Systemic therapy of neuroendocrine neoplasia: single center experience from a cohort of 110 consecutive cases. *Int J Endocrinol* 2020;**2020**:1491475.
3. Robelin P, Hadoux J, Forestier J, et al. Characterization, prognosis, and treatment of patients with metastatic lung carcinoid tumors. *J Thorac Oncol* 2019;**14**(6):993-1002.

Non-English text (n=3)

1. Estorch M. Treatment with (177)Lu-DOTATATE: past, present and future. *Rev Esp Med Nucl Imagen Mol* 2017;**36**(2):69-71.
2. Hervás I, Bello P, Falgas M, et al. (177)Lu-DOTATATE treatment in neuroendocrine tumours. A preliminary study. *Rev Esp Med Nucl Imagen Mol* 2017;**36**(2):91-8.
3. Medina-Ornelas SS, García-Pérez FO. Effectiveness of radiolabelled somatostatin analogues ((90)Y-DOTATOC and (177)Lu-DOTATATE) in patients with metastatic neuroendocrine tumours: a single centre experience in Mexico. *Rev Esp Med Nucl Imagen Mol* 2017;**36**(3):166-74.

Non-separable data between adults and pediatrics (n=1)

1. Parghane RV, Talole S, Prabhash K, Basu S. Clinical response profile of metastatic/advanced pulmonary neuroendocrine tumors to peptide receptor radionuclide therapy with ^{177}Lu-DOTATATE. *Clin Nucl Med* 2017;**42**:428-35.

Duplicated record (n=1)

1. Thang SP, Lung MS, Kong G, et al. Correction to: Peptide receptor radionuclide therapy (PRRT) in European Neuroendocrine Tumour Society (ENETS) grade 3 (G3) neuroendocrine neoplasia (NEN) - a single-institution retrospective analysis. *Eur J Nucl Med Mol Imaging* 2018;**45**(2):323.
